# Visible-light-mediated synthesis of oxime esters *via* multicomponent reactions of aldehydes, aryl amines, and *N*-hydroxyphthalimide esters†

**DOI:** 10.1039/d3ra06737h

**Published:** 2023-10-26

**Authors:** Anh Thu Nguyen, Hee-Kwon Kim

**Affiliations:** a Department of Nuclear Medicine, Jeonbuk National University Medical School and Hospital Jeonju 54907 Republic of Korea hkkim717@jbnu.ac.kr; b Research Institute of Clinical Medicine of Jeonbuk National University-Biomedical Research Institute of Jeonbuk National University Hospital Jeonju 54907 Republic of Korea

## Abstract

Oxime esters are useful scaffolds in many organic chemistry transformations. Herein, a novel visible-light-mediated three-component reaction for synthesis of oxime esters is reported. Aldehydes, aniline, and *N*-hydroxyphthalimide (NHPI) esters were used as substrates in this three-component reaction, and eosin Y was used as a crucial photocatalyst for the reaction. Wide ranges of aldehydes and NHPI esters were well tolerated in this reaction method, generating various oxime esters with high efficiency under mild reaction conditions. This visible-light-mediated methodology will be a promising approach to synthesize useful oxime esters in a single step.

## Abstract 2

Practical and efficient visible-light-induced synthesis of oxime esters has been achieved *via* multicomponent reactions in the presence of catalytic eosin Y.

## Graphical abstract

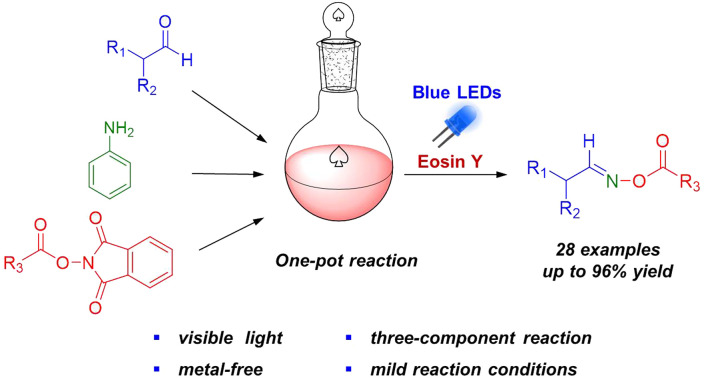

## Introduction

**Scheme 1:**
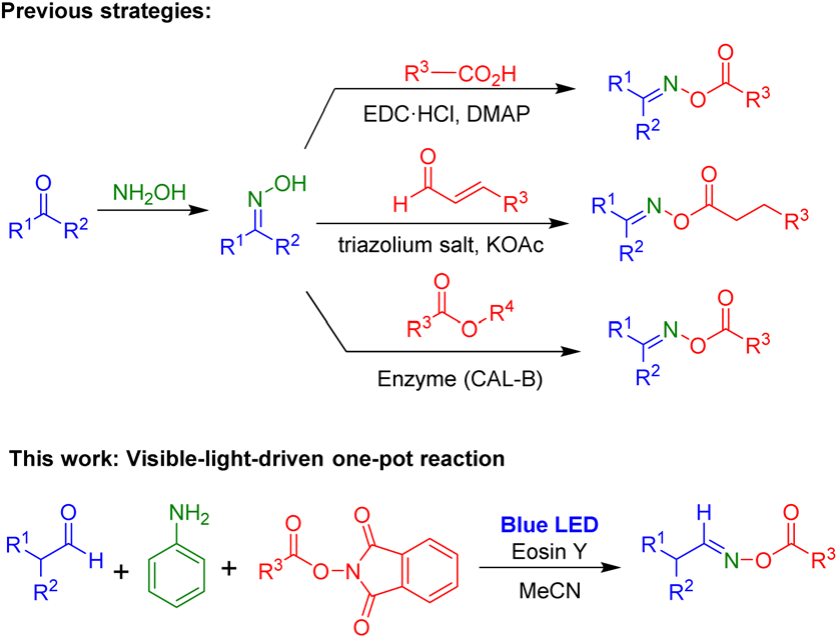
Synthetic procedures affording oxime esters.

Oxime esters have been found in various areas. For example, a wide range of biologically active compounds with anticancer,^1^ anti-inflammatory,^2^ antifungal,^3^ antioxidant and antimicrobial^4^ activities contain oxime ester moieties. Additionally, oxime esters are an important scaffold for many organic synthesis processes such as C–C bond cleavage,^5^ cyanoalkylation,^6^ hydroesterification,^7^ cross coupling,^8^ sulfonylamination,^9^ and cyclization.^10^ Recently, oxime esters were used as photoinitiators in photoreactions for various transformations.^11^ Due to their utility, several methods have been reported for the synthesis of oxime esters (Scheme 1). Oxime esters can be prepared using two-step procedures: the formation of aldoximes and ketoximes *via* reactions of aldehydes or ketones with NH_2_OH, followed by esterification of oximes with carboxylic acids,^12^ aldehydes,^13^ or esters.^14^

Light has been employed for numerous chemical transformations.^15^ Photochemical reactions are initiated by the absorption of light as the source of energy, and they have become useful methods with many applications in organic chemistry and materials.^16^ Additionally, photochemical reactions are usually carried out at room temperature and can tolerate a wide range of transformations.^17^

*N*-Hydroxyphthalimide (NHPI) esters also are useful substrates in a variety of reactions because they can be prepared with ease and at low cost from carboxylic acids and *N*-hydroxyphthalimides. NHPI esters have been used in many reactions for the formation of C–C bonds.^18^ Reactions of NHPI esters to generate C–B and C–Si bonds have been developed.^19^ Various photoredox reactions employing NHPI esters also have been reported. These reactions included formation of C–C bonds such as alkylation of heterocycles^20^ and alkenylation,^21^ formation of C–O and C–S bonds,^22^ and remote C–H oxidation.^23^ Additionally, NHPI esters were successfully employed in annulation reactions under visible-light irradiation to produce saturated heterocycles.^24^

We were interested in developing practical and useful synthetic methods for oxime ester compounds. Specifically, utilization of a visible-light-mediated reaction to yield oxime esters can be a promising strategy. Herein, we describe a visible-light-induced one-pot reaction *via* three-component reaction of aldehyde, aniline, and NHPI esters for the preparation of oxime ester compounds (Scheme 1).

## Results and discussion

One-pot reactions are useful approaches to produce organic compounds with many advantages including minimal waste and reduced cost and effort.^25^ Thus, we tried to use multicomponent reactions for the preparation of oxime esters. For the present study, we assumed that reaction of aldehydes with amines could also produce the radical acceptors imines, and, under irradiation by visible light, NHPI esters could be converted into carboxylic radicals. Then, target oxime esters could be formed through reaction between imines and carboxylic radicals.

To assess our idea, novel visible-light-induced multicomponent reactions using aldehydes, aryl amine, and NHPI ester in the presence of a photocatalyst were examined. In the initial experiments, benzaldehyde, aniline, and 1,3-dioxoisoindolin-2-yl butyrate (NHPI ester of butyric acid) were selected as the model substrates, and eosin Y was employed as a photocatalyst for optimization of the reaction condition (Table 1) because eosin Y has been used for visible-light induced reactions.^26^ The reaction using eosin Y in MeCN was conducted under irradiation of blue LEDs at room temperature. Under standard conditions, (*E*)-benzaldehyde *O*-butyryl oxime, the target product, was obtained in 92% yield in 16 hours (Table 1, entry 1). The other photocatalysts were tested (Table 1, entries 2–8). The reaction using Ru(bpy)_3_Cl_2_·6H_2_O did not produce the desired product (Table 1, entry 2), and the use of 2,2-bipyridine or benzophenone resulted in preparation of the target product in less than 30% yield (Table 1, entries 3 and 4). Photocatalysts 5,5-dimethyl-2,2-bipyridine, salicylaldehyde, sodium anthraquinone-2-sulfonate (SAQS), and eosin B afforded the corresponding products in yields ranging from 64% to 90% (Table 1, entries 5–8).

**Table 1:** Screening of reaction conditions for synthesis of ethyl (*E*)-2-phenyldiazene-1-carboxylate^a^

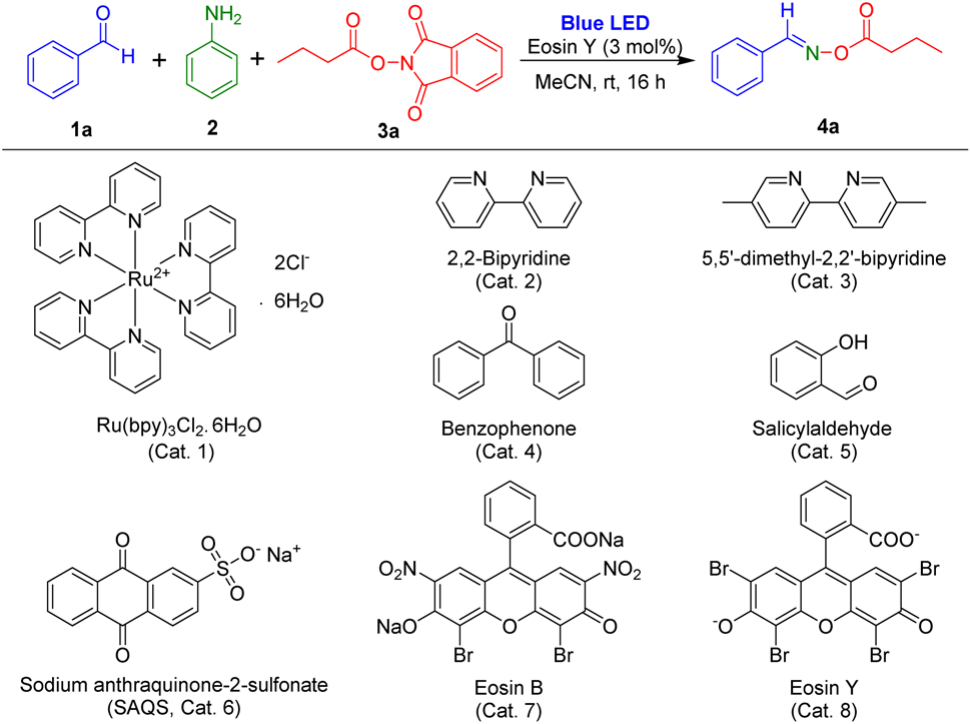		
Entry	Deviation from the standard conditions	Yield^b^ (%)
1	Standard conditions, blue LEDs	92
2	Cat. 1 instead of Cat. 8	NR^c^
3	Cat. 2 instead of Cat. 8	27
4	Cat. 3 instead of Cat. 8	28
5	Cat. 4 instead of Cat. 8	64
6	Cat. 5 instead of Cat. 8	76
7	Cat. 6 instead of Cat. 8	77
8	Cat. 7 instead of Cat. 8	90
9	CH_2_Cl_2_ instead of MeCN	15
10	DCE instead of MeCN	16
11	Toluene instead of MeCN	18
12	THF instead of MeCN	58
13	DMF instead of MeCN	79
14	1,4-Dioxane instead of MeCN	81
15	Ethyl acetate instead of MeCN	85
16	Benzylamine instead of 2	3
17	Propylamine instead of 2	Trace
18	*tert*-Butylamine instead of 2	NR
19	No photocatalyst	NR
20	No light	14
21	No photocatalyst, no light	NR

a Reaction conditions: aldehyde 1a (1.0 mmol), aniline 2 (1.0 mmol), *N*-hydroxyphthalimide ester (NHPI ester) 3a (1.2 mmol), photocatalyst (0.03 mmol), solvent (2 mL), room temperature, blue LEDs (5 W × 2 bulbs), 16 h.

b Isolated yield after purification by flash column chromatography.

c No reaction.

Several solvents including CH_2_Cl_2_, DCE, and toluene were also tested in photoreactions, but the reactions in these solvents produced oxime ester in less than 20% yield (Table 1, entries 9–11). Other solvents of THF, DMF, 1,4-dioxane, and ethyl acetate provided improved reaction efficiencies (Table 1, entries 12–15). However, MeCN was the most suitable solvent for this multi-component reaction. In addition, photoreactions using other nitrogen sources were also conducted. However, these reactions were not successful (Table 1, entries 16–18). Notably, using these basic aliphatic amines led to the hydrolysis of NHPI ester substrates and benzaldehyde substrate remained unreacted, indicating the vital role of aniline in the reaction. The influences of catalyst and light on the reaction were examined. The desired product was not prepared and only imine intermediate was generated in the reaction in the absence of photocatalyst (Table 1, entries 19 and 21). Reaction in the absence of light was performed, and less than 15% of product was obtained (Table 1, entry 20).

Next, several different light types were tested to examine their influence on the reaction (Table S1†). Irradiation with white LEDs, green LEDs, and compact fluorescent lights (CFLs) led to generation of products with yields of 71%, 87%, and 88%, respectively. However, no other additional improvements of reaction were observed compared to the reaction using blue LEDs. Different amounts of photocatalyst were investigated to determine optimal reaction conditions (Table S2†). Use of 1 mol% of eosin Y as a catalyst yielded the desired oxime ester in 76% yield, and a reaction using 3 mol% of eosin Y boosted the yield to 92%. However, greater than 3 mol% of eosin Y did not show an additional increase. Amounts of 1,3-dioxoisoindolin-2-yl butyrate in the reaction were also examined, and reaction using 1.2 equiv. of 1,3-dioxoisoindolin-2-yl butyrate afforded the target product in high yield (Table S3†).

**Scheme 2:**
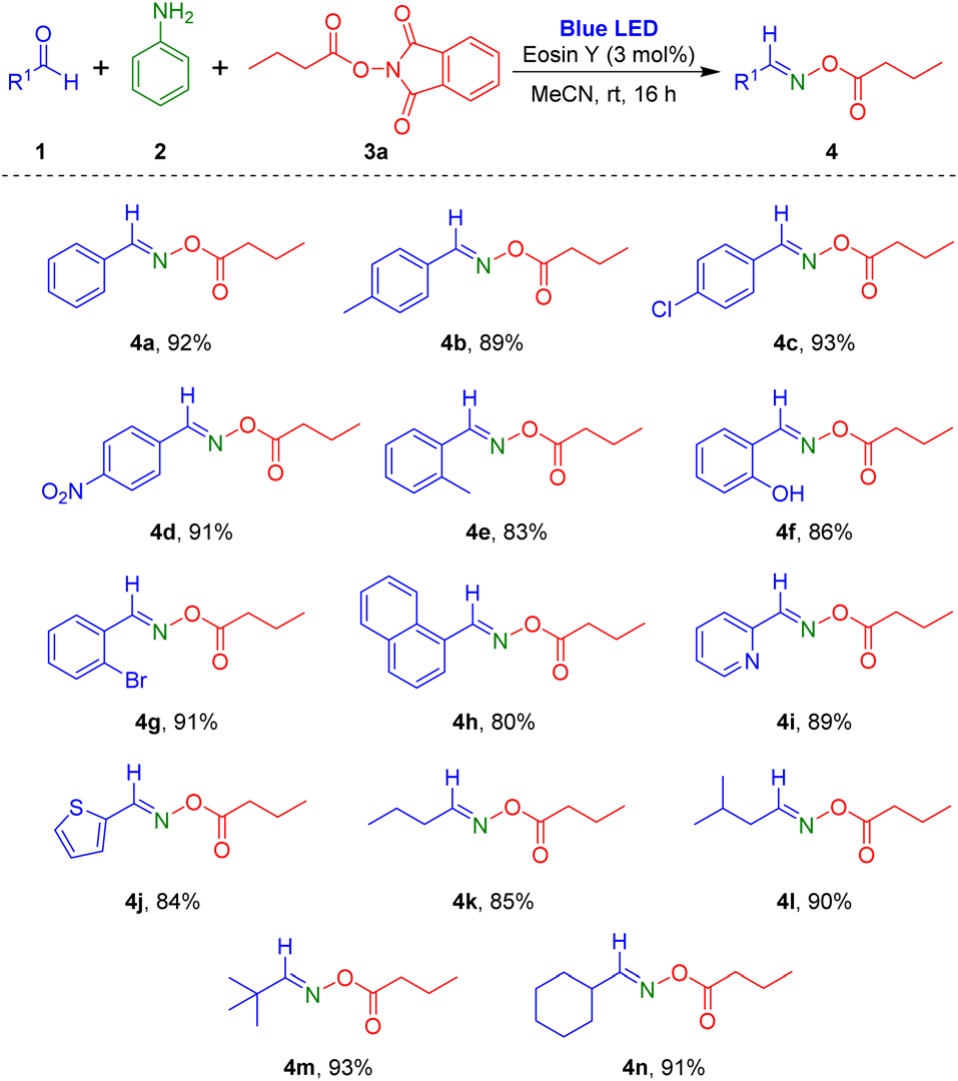
Substrate scope of aldehydes. ^*a*^Reaction conditions: aldehyde 1 (1.0 mmol), aniline 2 (1.0 mmol), *N*-hydroxyphthalimide ester (NHPI ester) 3a (1.2 mmol), eosin Y (0.03 mmol), MeCN (2 mL), blue LEDs (5 W × 2 bulbs), 16 h. ^*b*^Isolated yield after purification by flash column chromatography.

Based on the optimized conditions, we explored the scope of the visible-light-induced reaction. First, various aldehydes were reacted with aniline and 1,3-dioxoisoindolin-2-yl butyrate (Scheme 2). Aryl aldehydes containing electron-donating (methyl) or electron-withdrawing substituents (chloro and nitro) in the *para*-position on the phenyl ring produced the desired products 4a–4d in high yields (89–93%). Reaction of aryl aldehydes with electron-donating or electron-withdrawing groups at the *ortho*-position of the aromatic ring also yielded the corresponding products 4e–4g at 83–91%. Notably, reaction of heterocyclic aromatic substrates 2-pyridinecarboxaldehyde and 2-thiophenecarboxaldehyde provided the target oxime esters (4i and 4j) in yields of 89% and 84%, respectively. Additionally, several alkyl aldehydes were tested for this reaction protocol, and they were tolerated in the reaction to afford the target oxime esters (4k–4n) in high yields.

**Scheme 3:**
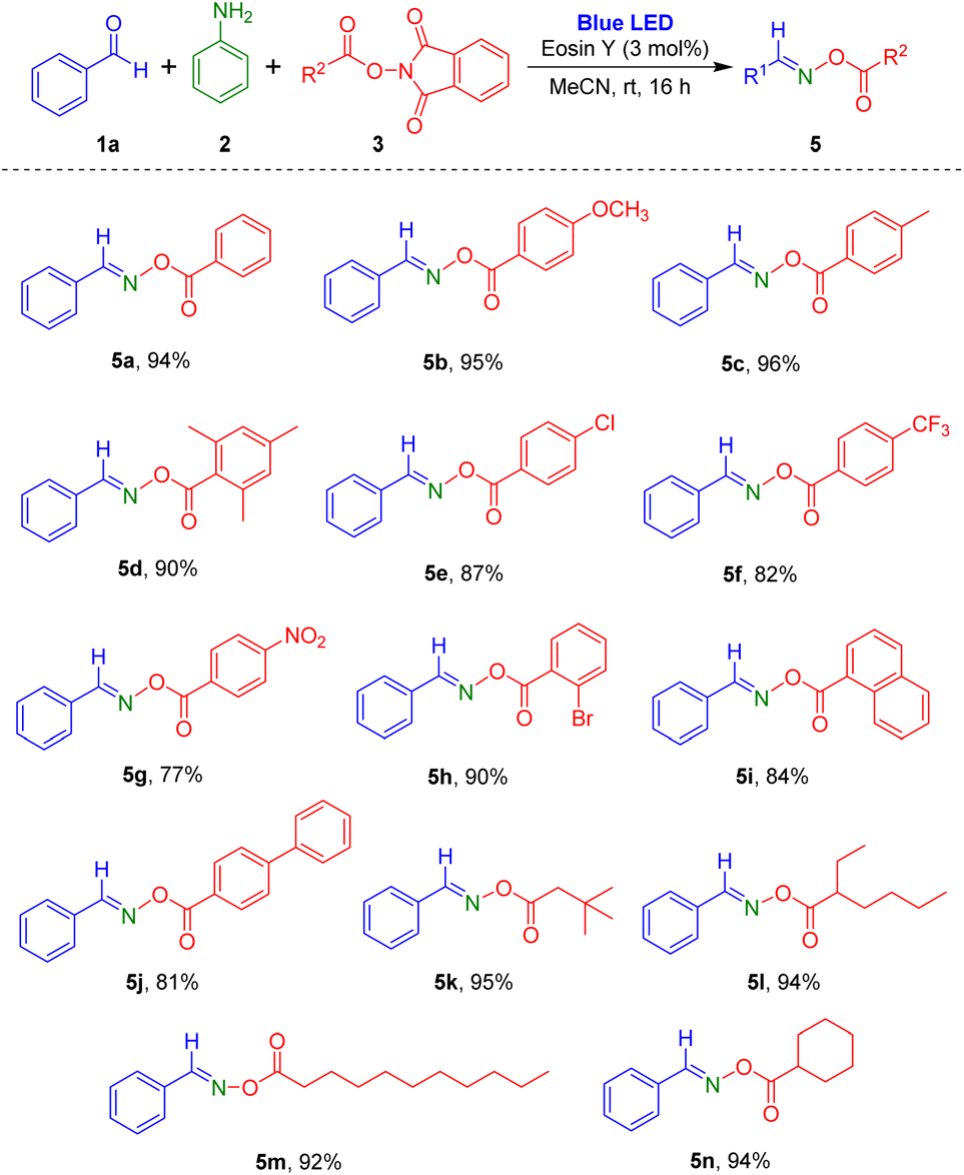
Substrate scope of NHPI esters. ^*a*^Reaction conditions: aldehyde 1a (1.0 mmol), aniline 2 (1.0 mmol), *N*-hydroxyphthalimide ester (NHPI ester) 3 (1.2 mmol), eosin Y (0.03 mmol), MeCN (2 mL), blue LEDs (5 W × 2 bulbs), 16 h. ^*b*^Isolated yield after purification by flash column chromatography.

Subsequently, reactions of a variety of NHPI esters with benzaldehyde and aniline were carried out (Scheme 3). Aromatic carboxylic acid-derived NHPI esters with electron-donating (methyl and methoxy) or electron-withdrawing substituents (chloro, bromo, trifluoromethyl, and nitro) readily produced the desired oxime esters (5a–5h). Particularly, reaction of NHPI ester of 2,4,6-trimethylbenzoic acid, which has steric hindrance, gave the target product 5d in 90% yield. Using the process, NHPI esters of the two aromatic carboxylic acids 1-naphthoic acid and biphenyl-4-carboxylic acid were also transformed to the corresponding oxime ester products (5i and 5j). Additionally, various alkyl carboxylic acid derived NHPI esters were employed in the reaction, and they were tolerated in this reaction method to give the desired products (5k–5n) in high yields. Notably, the reaction of NHPI esters prepared from undecanoic acid, which contains a long carbon chain, generated the target oxime ester compound 5m in 92% yield.

**Scheme 4:**
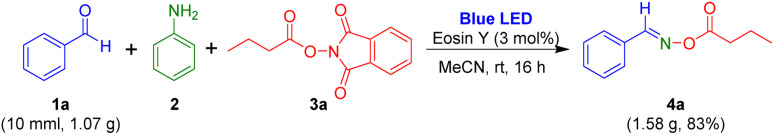
Gram-scale reaction of three components substrate scope of aldehydes.

Next, gram-scale synthesis of oxime esters from aldehyde, aniline, and 1,3-dioxoisoindolin-2-yl butyrate was performed to evaluate the visible-light-mediated multi-component reaction (Scheme 4). Benzaldehyde 1a (10.0 mmol, 1.07 g) readily reacted with aniline and 1,3-dioxoisoindolin-2-yl butyrate to generate the target oxime ester 4a in 83% yield.

**Scheme 5:**
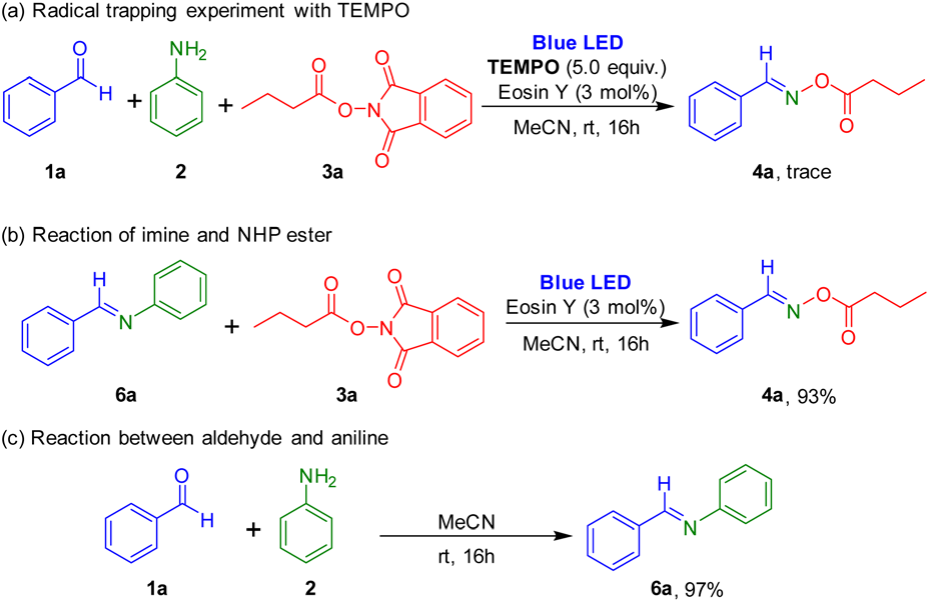
Control experiments.

Several control experiments were carried out to understand the reaction mechanism (Scheme 5). When 5.0 equiv. of TEMPO (2,2,6,6-tetramethyl-1-piperidinyloxy), a radical scavenger, was added to this reaction, formation of the target oxime ester was prevented, which suggested that the synthesis used a radical pathway. Reaction of imine and 1,3-dioxoisoindolin-2-yl butyrate in the presence of photocatalyst was performed under the irradiation of blue LEDs, and the target product was successfully obtained. Also, the reaction between aldehyde and aniline readily afforded the imine.

**Fig. 1:**
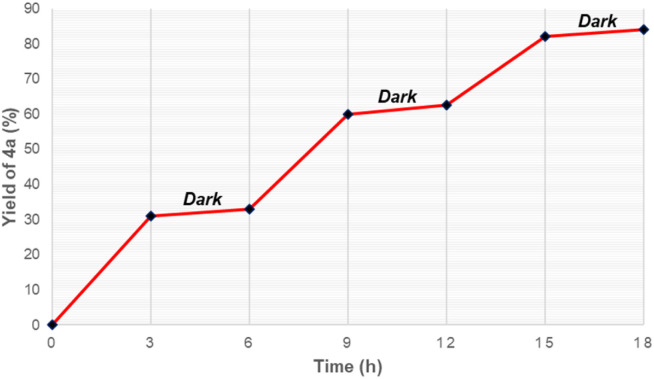
Light on/off experiments.

Moreover, light on/off experiments were conducted to assess the effect of visible light on this synthesis (Fig. 1). Synthesis of oxime ester was remarkably slow in the dark, while irradiation by blue LEDs readily generated the desired product 4a from starting substrates. This suggests that irradiation of visible light played a crucial role in this reaction.

**Scheme 6:**
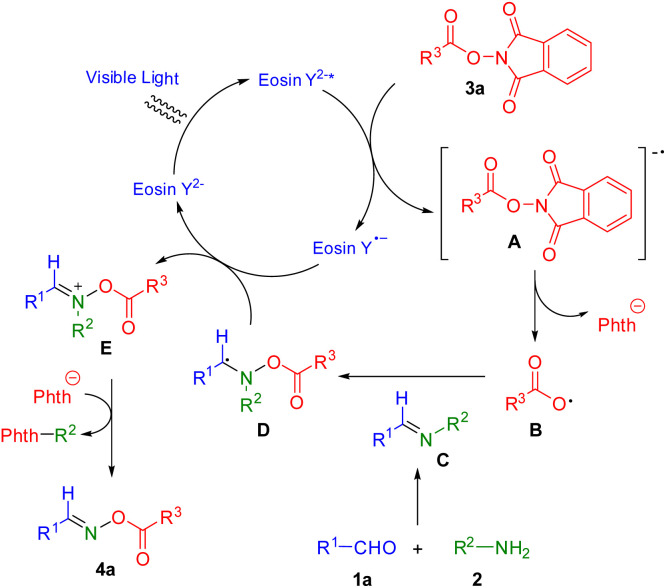
A plausible mechanism.

We proposed a plausible catalytic cycle mechanism for this process (Scheme 6) based on the results of control experiments and previously published reports.^27^ Photocatalyst eosin Y^2−^ forms eosin Y^2−^* under irradiation of blue LEDs. NHPI ester 3a is transformed to radical anion A*via* reaction of eosin Y^2−^*. Then, radical anion A yields carboxylic radical B by release of a phthalimide anion. Carboxylic radical B reacts with imine C, which is prepared by treatment of aldehyde 1a and amine 2, to give C-centered radical intermediate D. Intermediate D provides the imine-like cation E*via* single electron transfer with eosin Y˙^−^ while photocatalyst eosin Y^2−^ is recovered to close the catalytic cycle. The desired product 4a is finally produced by the elimination of the phenyl group with phthalimide anion.

## Experimental

### General procedure of the synthesis of oxime esters (4a–4n, 5a–5n)

Benzaldehyde 1a (0.106 g, 1.0 mmol, 1.0 equiv.), aniline 2 (0.093 g, 1.0 mmol), 1,3-dioxoisoindolin-2-yl butyrate (NHPI ester) 3a (0.233 g, 1.2 mmol), and eosin Y (0.021 g, 0.03 mmol) were added to acetonitrile (2 mL). The reaction mixture was stirred under irradiation of 5 W blue LEDs (×2) at room temperature for 16 hours. The reaction product was extracted with EtOAc (50 mL) and washed with aqueous NaHCO_3_ (50 mL), followed by water (50 mL). The organic layer was dried with anhydrous sodium sulfate and was concentrated under reduced pressure. The residue was purified by flash column chromatography on silica gel eluting with hexane–EtOAc as eluent to afford the target product (4a) as a yellow oil (0.175 g, 92%).

## Conclusions

In summary, efficient visible-light-mediated method for the preparation of oxime esters through one-pot multicomponent reaction employing aldehydes, aniline, and NHPI esters was described. Eosin Y was used as a suitable photocatalyst for the synthesis of oxime esters. A wide range of oxime esters bearing aliphatic and aromatic groups was successfully obtained by novel visible-light-mediated reaction under mild conditions. The method has advantages such as use of a wide scope of substrates, simple operation, and mild reaction conditions. Thus, this method allows easy and effective preparation of oxime esters in one step.

## Author contributions

H.-K. Kim: conceptualization, supervision, data interpretation, writing – original draft, review, and editing. A. T. Nguyen: investigation, methodology, data curation, data interpretation, writing – original draft, and editing.

## Conflicts of interest

There are no conflicts to declare.

## Supplementary Material

RA-013-D3RA06737H-s001

## References

[cit1] Chen H.-P., Zhao Z.-Z., Li Z.-H., Dong Z.-J., Wei K., Bai X., Zhang L., Wen C.-N., Feng T., Liu J.-K. (2016). Novel natural oximes and oxime esters with a vibralactone backbone from the basidiomycete Boreostereum vibrans. ChemistryOpen.

[cit2] Li Q., Zhang J., Chen L. Z., Wang J. Q., Zhou H. P., Tang W. J., Xue W., Liu X. H. (2018). New pentadienone oxime ester derivatives: synthesis and anti-inflammatory activity. J. Enzyme Inhib. Med. Chem..

[cit3] Fan Z., Duan W., Lin G., Chen M., Huang M. (2021). Synthesis and biological activities of novel (*Z*)-/(*E*)-anisaldehyde-based oxime ester compounds. Chem. Biodiversity.

[cit4] Harini S. T., Kumar H. V., Rangaswamy J., Naik N. (2012). Synthesis, antioxidant and antimicrobial activity of novel vanillin derived piperidin-4-one oxime esters: preponderant role of the phenyl ester substituents on the piperidin-4-one oxime core. Bioorg. Med. Chem. Lett..

[cit5] He Y., Lou J., Wu K., Wang H., Yu Z. (2019). Copper-catalyzed radical C–C bond cleavage and [4+1] annulation cascade of cycloketone oxime esters with enaminothiones. J. Org. Chem..

[cit6] Yang L., Gao P., Duan X.-H., Gu Y.-R., Guo L.-N. (2018). Direct C–H cyanoalkylation of quinoxalin-2(1*H*)-ones via radical C–C bond cleavage. Org. Lett..

[cit7] Sang T., Zhou C., Li J., Liu X., Yuan Y., Bao X., Zhao X., Huo C. (2023). Photosensitized anti-Markovnikov hydroesterification of unactivated alkenes. Org. Lett..

[cit8] Li G.-Q., Meng F.-R., Xiao W.-J., Chen J.-R. (2023). Photoinduced copper-catalyzed asymmetric radical three-component cross-coupling of 1,3-enynes with oxime esters and carboxylic acids. Org. Chem. Front..

[cit9] Yuan C.-P., Zheng Y., Xie Z.-Z., Deng K.-Y., Chen H.-B., Xiang H.-Y., Chen K., Yang H. (2023). Photosensitized vicinal sulfonylamination of alkenes with oxime ester and DABCO·(SO_2_)_2_. Org. Lett..

[cit10] Wang S.-C., Shen Y.-T., Zhang T.-S., Hao W.-J., Tu S.-J., Jiang B. (2021). Cyclic oxime esters as deconstructive bifunctional reagents for cyanoalkyl esterification of 1,6-enynes. J. Org. Chem..

[cit11] Hammoud F., Hijazi A., Schmitt M., Dumur F., Lalevée J. (2023). A review on recently proposed oxime ester photoinitiators. Eur. Polym. J..

[cit12] Kumar C. S., Kumar N. V., Srinivas P., Bettadaiah B. K. (2014). A convenient practical synthesis of alkyl and aryl oxime esters. Synthesis.

[cit13] Enders D., Grossmann A., Craen D. V. (2013). *N*-Heterocyclic carbene catalyzed synthesis of oxime esters. Org. Biomol. Chem..

[cit14] Lal S., Snape T. J. (2012). Exploitation of a *Candida antarctica* lipase B-catalysed *in situ* carboxylic acid activation method for the synthesis of acetanilides. J. Mol. Catal. B: Enzym..

[cit15] Patil D. V., Hong Y. T., Kim H. Y., Oh K. (2022). Visible-light-induced three-component selenofunctionalization of alkenes: an aerobic selenol oxidation approach. Org. Lett..

[cit16] Kumar G. S., Lin Q. (2021). Light-triggered click chemistry. Chem. Rev..

[cit17] Liu Y., Pang T., Yao W., Zhong F., Wu G. (2023). Visible-light-induced radical *gem*-iodoallylation of 2,2,2-trifluorodiazoethane. Org. Lett..

[cit18] Kang K., Weix D. J. (2022). Nickel-catalyzed C(sp^3^)–C(sp^3^) cross-electrophile coupling of in situ generated NHP esters with unactivated alkyl bromides. Org. Lett..

[cit19] Li C., Wang J., Barton L. M., Yu S., Tian M., Peters D. S., Kumar M., Yu A. W., Johnson K. A., Chatterjee A. K., Yan M., Baran P. S. (2017). Decarboxylative borylation. Science.

[cit20] Kammer L. M., Rahman A., Opatz T. (2018). A visible light-driven Minisci-type reaction with n-hydroxyphthalimide esters. Molecules.

[cit21] Wang H.-Y., Zhong L.-J., Lv G.-F., Li Y., Li J.-H. (2020). Photocatalytic dual decarboxylative alkenylation mediated by triphenylphosphine and sodium iodide. Org. Biomol. Chem..

[cit22] Shibutani S., Kodo T., Takeda M., Nagao K., Tokunaga N., Sasaki Y., Ohmiya H. (2020). Organophotoredox-catalyzed decarboxylative C(sp^3^)–O bond formation. J. Am. Chem. Soc..

[cit23] Luo M., Zhu S., Shi C., Du Y., Yang C., Guo L., Xia W. (2022). Photoinduced remote C(sp^3^)–H cyanation and oxidation enabled by a vinyl radical-mediated 1,5-HAT strategy. Org. Lett..

[cit24] Murray P. R. D., Leibler I. N.-M., Hell S. M., Villalona E., Doyle A. G., Knowles R. R. (2022). Radical redox annulations: a general light-driven method for the synthesis of saturated heterocycles. ACS Catal..

[cit25] Hayashi Y. (2016). Pot economy and one-pot synthesis. Chem. Sci..

[cit26] Srivastava V., Singh P. K., Singh P. P. (2022). Recent advances of visible-light photocatalysis in the functionalization of organic compounds. J. Photochem. Photobiol., C.

[cit27] Cai Y., Zhang R., Sun D., Xu S., Zhou Q. (2017). Eosin Y-sensitized photocatalytic reaction of tertiary aliphatic amines with arenesulfonyl chlorides under visible-light irradiation. Synlett.

