# Supplementary material for: Visible-light-mediated synthesis of oxime esters via multicomponent reactions of aldehydes, aryl amines, and N-hydroxyphthalimide esters
Source: RSC Adv. 2023 Oct 26;13(45):31346–52. doi: 10.1039/d3ra06737h (PMC10600831; doi:10.1039/d3ra06737h)
Supplement: RA-013-D3RA06737H-s001 [file RA-013-D3RA06737H-s001.pdf]

Electronic Supplementary Material (ESI) for RSC Advances.
This journal is © The Royal Society of Chemistry 2023

Supplementary Information

Visible-Light-Mediated Synthesis of Oxime Esters via Multicomponent Reactions of Aldehydes, Aryl Amines, and N-Hydroxyphthalimide Esters

Anh Thu Nguyen[1] and Hee-Kwon Kim[1,2]*

[1] Department of Nuclear Medicine, Jeonbuk National University Medical School and Hospital, Jeonju, 54907, Republic of Korea

[2] Research Institute of Clinical Medicine of Jeonbuk National University-Biomedical Research Institute of Jeonbuk National University Hospital, Jeonju, 54907, Republic of Korea

* Corresponding author.

E-mail address: hkkim717@jbnu.ac.kr (H. Kim).

Table of Contents

Table of Contents S1

General Information S2

Screening of reaction conditions for the synthesis of oxime esters..S3

General procedure of the synthesis of oxime esters S6

Chracterization of oxime esters S7

^{1}H and ^{13}C NMR Spectra..S20

1. General Information

Commercial chemicals and solvents were used without any purification. Reaction progress was analyzed by thin-layer chromatography (TLC) using silica gel 60 F_{254} pre-coated aluminum plate from Merck and TLC spots were observed under UV light (254nm) exposure. Flash chromatography was carried out using 230–400 mesh silica gel and analytical grade solvents. Stuart SMP10 Melting Point Apparatus was used to record melting points of products. Structure elucidation by NMR (^{1}H and ^{13}C NMR) was performed on Bruker Avance 400 MHz spectrometer. The chemical shifts were reported in δ units (ppm) relative to the residual protonated solvent resonance, the coupling constants (*J*) quoted in Hz, and multiplicity of signals was abbreviated as follows: singlet (s); doublet (d); doublet of doublet (dd); triplet (t); multiplet (m).

2. Screening of reaction conditions for the synthesis of oxime esters

Table S1. Screening of reaction condition in the synthesis of oxime esters[a]

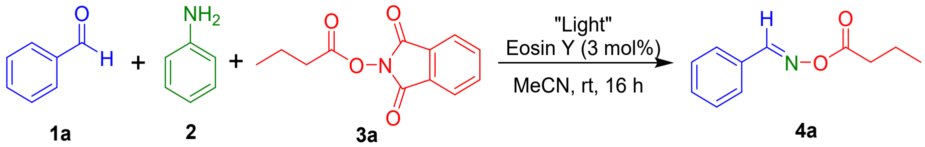

1a **2** **3a** **4a**

Entry	Light source	Yield[b] (%)
1	CFL	71
2	White LED	87
3	Green LED	88
4	Blue LED	92

[a] Reaction conditions: aldehyde 1a (1.0 mmol), aniline 2 (1.0 mmol), N-hydroxyphthalimide ester 3a (1.2 mmol), eosin Y (0.03 mmol), MeCN (2 mL), room temperature, blue LEDs (5 W x 2 bulbs), 16 h. [b] Isolated yield after purification by flash column chromatography.

Table S2. Amount of eosin Y in the preparation of oxime esters.

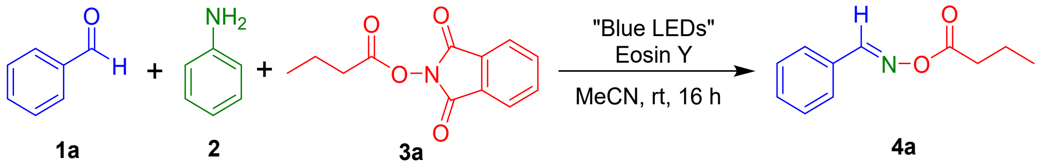

1a 2 3a 4a

Entry	Eosin Y	Yield[b] (%)
1	Eosin Y (0.00 equiv.)	trace
2	Eosin Y (0.01 equiv.)	76
3	Eosin Y (0.03 equiv.)	92
4	Eosin Y (0.05 equiv.)	92
5	Eosin Y (0.1 equiv.)	92
6	Eosin Y (0.2 equiv.)	92

[a] Reaction conditions: aldehyde 1a (1.0 mmol), aniline 2a (1.0 mmol), N-hydroxyphthalimide ester 3a (1.2 mmol), eosin Y, MeCN (2 mL), blue LEDs (5 W x 2 bulbs), 16 h. [b] Isolated yield after purification by flash column chromatography.

Table S3. Amount of 1,3-dioxoisoindolin-2-yl butyrate in the preparation of oxime esters.

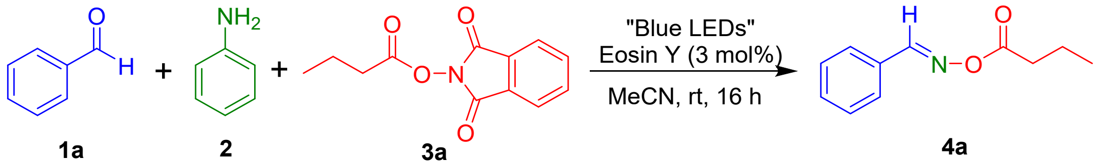

Entry	1,3-dioxoisoindolin-2-yl butyrate (3a)	Yield[b] (%)
1	1,3-dioxoisoindolin-2-yl butyrate (2.0 equiv)	92
2	1,3-dioxoisoindolin-2-yl butyrate (1.5 equiv)	92
3	1,3-dioxoisoindolin-2-yl butyrate (1.2 equiv)	92
4	1,3-dioxoisoindolin-2-yl butyrate (1.0 equiv.)	84
5	1,3-dioxoisoindolin-2-yl butyrate (0.8 equiv.)	73

[a] Reaction conditions: aldehyde 1a (1.0 mmol), aniline 2 (1.0 mmol), N-hydroxyphthalimide ester 3a (1.2 mmol), eosin Y (0.03 mmol), MeCN (2 mL), blue LEDs (5 W x 2 bulbs), 16 h. [b] Isolated yield after purification by flash column chromatography.

3. General procedure of the synthesis of oxime esters (4a-4n, 5a-5n)

Benzaldehyde **1a** (0.106 g, 1.0 mmol, 1.0 equiv.), aniline **2a** (0.093 g, 1.0 mmol), 1,3-dioxoisoindolin-2-yl butyrate (NHP ester) **3a** (0.233 g, 1.2 mmol), and eosin Y (0.021 g, 0.03 mmol) were added to acetonitrile (2 mL). The reaction mixture was stirred under irradiation of 5 W blue LEDs (×2) at room temperature for 16 hours. The reaction product was extracted with EtOAc (50 mL) and washed with aqueous $NaHCO_3$ (50 mL), followed by water (50 mL). The organic layer was dried with anhydrous sodium sulfate and was concentrated under reduced pressure. The residue was purified by flash column chromatography on silica gel eluting with hexane-EtOAc as eluent to afford the target product (**4a**) as a yellow oil (0.175 g, 92%).

4. Characterization data for oxime esters

(*E*)-Benzaldehyde O-butyryl oxime (4a)

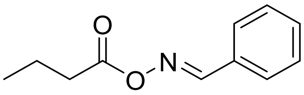

4a was obtained in 92% yield (175.7 mg) according to the general procedure (Hexan/EtOAc, 10:1); yellow oil; ^{1}H NMR (400 MHz, $CDCl_3$) δ 8.56 (s, 1H), 7.75-7.72 (m, 2H), 7.49-7.39 (m, 3H), 2.46 (t, J = 7.2 Hz, 2H), 1.82-1.72 (m, 2H), 1.02 (t, J = 7.6 Hz, 3H); ^{13}C NMR (100 MHz, $CDCl_3$) δ 171.2, 155.9, 131.7, 130.2, 128.9 (2C), 128.4 (2C), 34.7, 18.4, 13.7; HRMS (ESI) m/z $(M+H)^+$ calcd for $C_{11}H_{14}NO_2^+$ = 192.1025; found 192.1022.

(*E*)-4-Methylbenzaldehyde O-butyryl oxime (4b)

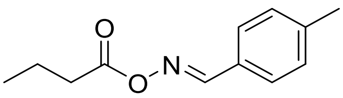

4b was obtained in 89% yield (182.4 mg) according to the general procedure (Hexan/EtOAc, 10:1); yellow oil; ^{1}H NMR (400 MHz, $CDCl_3$) δ 8.32 (s, 1H), 7.64 (d, J = 8.0 Hz, 2H), 7.24 (d, J = 8.0 Hz, 2H), 2.47-2.39 (m, 5H), 1.81-1.72 (m, 2H), 1.02 (t, J = 7.2 Hz, 3H); ^{13}C NMR (100 MHz, $CDCl_3$) δ 171.2, 155.9, 142.2, 129.6 (2C), 128.4 (2C), 127.4, 34.7, 21.6, 18.4, 13.7; HRMS (ESI) m/z $(M+H)^+$ calcd for $C_{12}H_{16}NO_2^+$ = 206.1181; found 206.1180.

(*E*)-4-Chlorobenzaldehyde O-butyryl oxime (4c)

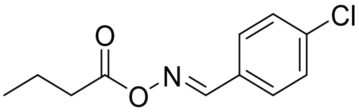

4c was obtained in 93% yield (209.2 mg) according to the general procedure (Hexan/EtOAc, 10:1); yellow oil; ^{1}H NMR (400 MHz, $CDCl_3$) δ 8.32 (s, 1H), 7.69-7.66 (m, 2H), 7.41-7.39 (m, 2H), 2.45 (t, J = 7.6 Hz, 2H), 1.81-1.71 (m, 2H), 1.02 (t, J = 7.6 Hz, 3H); ^{13}C NMR (100 MHz, $CDCl_3$) δ 170.9, 154.7, 137.8, 129.5 (2C), 129.3 (2C), 128.7, 34.6, 18.4, 13.7; HRMS (ESI) m/z $(M+H)^+$ calcd for $C_{11}H_{13}ClNO_2^+$ = 226.0635; found 226.0636.

(*E*)-4-Nitrobenzaldehyde O-butyryl oxime (4d)

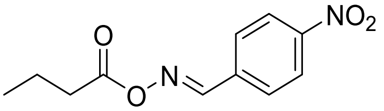

4d was obtained in 91% yield (214.7 mg) according to the general procedure (Hexan/EtOAc, 10:1); yellow oil; ^{1}H NMR (400 MHz, $CDCl_3$) δ 8.43 (s, 1H), 8.29-8.27 (m, 2H), 7.94-7.91 (m, 2H), 2.48 (t, J = 7.2 Hz, 2H), 1.80-1.75 (m, 2H), 1.03 (t, J = 7.2 Hz, 3H); ^{13}C NMR (100 MHz, $CDCl_3$) δ 170.6, 153.5, 149.5, 136.3, 129.1 (2C), 124.1 (2C), 34.5, 18.3, 13.6; HRMS (ESI) m/z $(M+H)^+$ calcd for $C_{11}H_{13}N_2O_4^+$ = 237.0875; found 237.0873.

(*E*)-2-Methylbenzaldehyde O-butyryl oxime (4e)

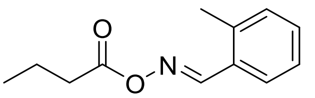

4e was obtained in 83% yield (170.2 mg) according to the general procedure (Hexan/EtOAc, 10:1); yellow oil; ^{1}H NMR (400 MHz, $CDCl_3$) δ 8.62 (s, 1H), 7.86 (d, J = 8.0 Hz, 1H), 7.35 (m, 1H), 7.24-7.22 (m, 2H), 2.49-2.45 (m, 5H), 1.82-1.73 (m, 2H), 1.03 (t, J = 7.2 Hz, 3H); ^{13}C NMR (100 MHz, $CDCl_3$) δ 171.3, 154.8, 138.1, 131.3, 130.9, 128.5, 128.2, 126.4, 34.7, 19.9, 18.4, 13.7; HRMS (ESI) m/z $(M+H)^+$ calcd for $C_{12}H_{16}NO_2^+$ = 206.1181; found 206.1180.

(*E*)-2-Hydroxybenzaldehyde O-butyryl oxime (4f)

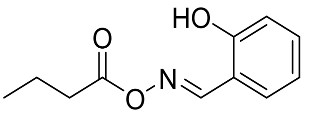

4f was obtained in 86% yield (178.0 mg) according to the general procedure (Hexan/EtOAc, 10:1); yellow oil; ^{1}H NMR (400 MHz, $CDCl_3$) δ 9.83 (br s, 1H), 8.42 (s, 1H), 7.40-7.35 (m, 1H), 7.24-7.22 (m, 1H), 7.06 (d, J = 8.0 Hz, 1H), 6.96-6.93 (m, 1H), 2.45 (t, J = 7.2 Hz, 2H), 1.82-1.72 (m, 2H), 1.03 (t, J = 7.2 Hz, 3H); ^{13}C NMR (100 MHz, $CDCl_3$) δ 169.7, 158.4, 157.3, 133.1, 131.9, 119.8, 117.5, 114.9, 34.3, 18.3, 13.7; HRMS (ESI) m/z $(M+H)^+$ calcd for $C_{11}H_{14}NO_3^+$ = 208.0974; found 208.0971.

(*E*)-2-Bromobenzaldehyde O-butyryl oxime (4g)

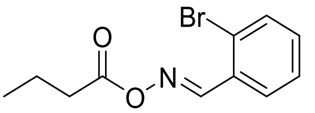

4g was obtained in 91% yield (245.7 mg) according to the general procedure (Hexan/EtOAc, 10:1); yellow oil; ^{1}H NMR (400 MHz, $CDCl_3$) δ 8.77 (s, 1H), 8.08-8.06 (m, 1H), 7.62-7.59 (m, 1H), 7.36-7.30 (m, 2H), 2.47 (t, J = 7.6 Hz, 2H), 1.83-1.73 (m, 2H), 1.03 (t, J = 7.2 Hz, 3H); ^{13}C NMR (100 MHz, $CDCl_3$) δ 170.9, 155.2, 133.2, 132.8, 129.8, 128.8, 127.8, 124.9, 34.6, 18.4, 13.7; HRMS (ESI) m/z $(M+H)^+$ calcd for $C_{11}H_{13}BrNO_2^+$ = 270.0130; found 270.0128.

(*E*)-1-Naphthaldehyde O-butyryl oxime (4h)

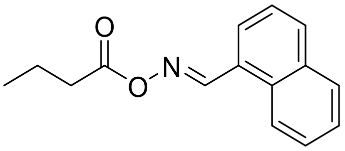

4h was obtained in 80% yield (192.8 mg) according to the general procedure (Hexan/EtOAc, 10:1); yellow oil; ^{1}H NMR (400 MHz, $CDCl_3$) δ 8.98 (s, 1H), 8.63-8.60 (m, 1H), 7.98-7.90 (m, 3H), 7.66-7.62 (m, 1H), 7.58-7.50 (m, 2H), 2.54-2.51 (m, 2H), 1.85-1.79 (m, 2H), 1.06 (t, J = 7.2 Hz, 3H); ^{13}C NMR (100 MHz, $CDCl_3$) δ 171.2, 155.8, 133.8, 132.3, 130.9, 129.6, 128.9, 127.8, 126.5, 126.3, 125.2, 124.6, 34.8, 18.4, 13.8; HRMS (ESI) m/z $(M+H)^+$ calcd for $C_{15}H_{16}NO_2^+$ = 242.1181; found 242.1179.

(*E*)-Picolinaldehyde O-butyryl oxime (4i)

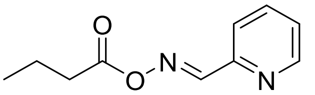

4i was obtained in 89% yield (170.8 mg) according to the general procedure (Hexan/EtOAc, 3:1); yellow oil; ^{1}H NMR (400 MHz, $CDCl_3$) δ 8.66 (d, J = 4.8 Hz, 1H), 8.45 (s, 1H), 8.10 (d, J = 8.0 Hz, 1H), 7.76 (td, J = 7.6, 1.6 Hz, 1H), 7.37-7.34 (m, 1H), 2.47 (t, J = 7.6 Hz, 2H), 1.81-1.72 (m, 2H), 1.01 (t, J = 7.2 Hz, 3H); ^{13}C NMR (100 MHz, $CDCl_3$) δ 170.8, 156.5, 150.0, 149.8, 136.8, 125.5, 122.1, 34.6, 18.3, 13.7; HRMS (ESI) m/z $(M+H)^+$ calcd for $C_{10}H_{13}N_2O_2^+$ = 193.0977; found 193.0975.

(*E*)-Thiophene-2-carbaldehyde O-butyryl oxime (4j)

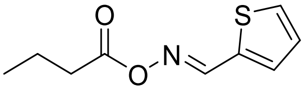

4j was obtained in 84% yield (165.4 mg) according to the general procedure (Hexan/EtOAc, 7:1); white solid; ^{1}H NMR (400 MHz, $CDCl_3$) δ 8.49 (s, 1H), 7.49-7.47 (m, 1H), 7.39 (d, J = 3.6 Hz, 1H), 7.09-7.08 (m, 1H), 2.43 (t, J = 7.2 Hz, 2H), 1.79-1.70 (m, 2H), 1.00 (t, J = 7.2 Hz, 3H); ^{13}C NMR (100 MHz, $CDCl_3$) δ 170.9, 150.3, 133.2, 132.8, 130.4, 127.6, 34.6, 18.4, 13.7; HRMS (ESI) m/z $(M+H)^+$ calcd for $C_9H_{12}NO_2S^+$ = 198.0589; found 198.0586.

(*E*)-Butyraldehyde O-butyryl oxime (4k)

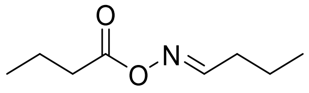

4k was obtained in 85% yield (133.4 mg) according to the general procedure (Hexan/EtOAc, 10:1); yellow oil; ^{1}H NMR (400 MHz, $CDCl_3$) δ 7.69 (t, J = 6.4 Hz, 1H), 2.44-2.32 (m, 4H), 1.76-1.67 (m, 2H), 1.64-1.54 (m, 2H), 1.03-0.97 (m, 6H); ^{13}C NMR (100 MHz, $CDCl_3$) δ 171.2, 159.1, 34.7, 31.3, 28.7, 19.7, 18.4, 13.7; HRMS (ESI) m/z $(M+H)^+$ calcd for $C_8H_{16}NO_2^+$ = 158.1181; found 158.1178.

(*E*)-3-Methylbutanal O-butyryl oxime (4l)

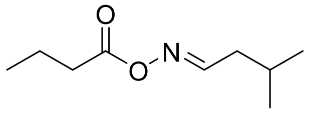

4l was obtained in 90% yield (153.9 mg) according to the general procedure (Hexan/EtOAc, 10:1); yellow oil; ^{1}H NMR (400 MHz, $CDCl_3$) δ 7.69 (t, J = 6.8 Hz, 1H), 2.34 (t, J = 7.6 Hz, 2H), 2.25 (t, J = 6.8 Hz, 2H), 1.94-1.84 (m, 1H), 1.76-1.67 (m, 2H), 1.00-0.96 (m, 9H); ^{13}C NMR (100 MHz, $CDCl_3$) δ 171.1, 158.6, 37.9, 34.6, 26.6, 22.3 (2C), 18.4, 13.7; HRMS (ESI) m/z $(M+H)^+$ calcd for $C_9H_{18}NO_2^+$ = 172.1338; found 172.1336.

(*E*)-Pivalaldehyde O-butyryl oxime (4m)

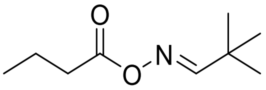

4m was obtained in 93% yield (159.0 mg) according to the general procedure (Hexan/EtOAc, 10:1); yellow oil; ^{1}H NMR (400 MHz, $CDCl_3$) δ 7.58 (s, 1H), 2.33 (t, J = 7.6 Hz, 2H), 1.73-1.64 (m, 2H), 1.16 (s, 9H), 0.96 (t, J = 7.2 Hz, 3H); ^{13}C NMR (100 MHz, $CDCl_3$) δ 171.3, 166.0, 34.7, 34.4, 27.1 (3C), 18.3, 13.7; HRMS (ESI) m/z $(M+H)^+$ calcd for $C_9H_{18}NO_2{}^+$ = 172.1338; found 172.1335.

(*E*)-Cyclohexanecarbaldehyde O-butyryl oxime (4n)

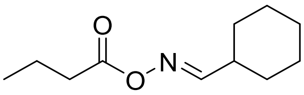

4n was obtained in 91% yield (179.2 mg) according to the general procedure (Hexan/EtOAc, 10:1); yellow oil; ^{1}H NMR (400 MHz, $CDCl_3$) δ 7.55 (d, J = 7.2 Hz, 1H), 2.45-2.32 (m, 3H), 1.84-1.68 (m, 7H), 1.36-1.19 (m, 5H), 1.01-0.96 (m, 3H); ^{13}C NMR (100 MHz, $CDCl_3$) δ 171.2, 162.6, 38.6, 34.7, 29.9 (2C), 25.6, 25.2 (2C), 18.4, 13.7; HRMS (ESI) m/z $(M+H)^+$ calcd for $C_{11}H_{20}NO_2{}^+$ = 198.1494; found 198.1492.

(*E*)-Benzaldehyde O-benzoyl oxime (5a)

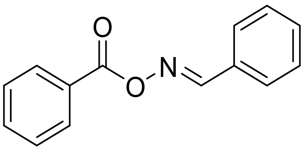

5a was obtained in 94% yield (211.5 mg) according to the general procedure (Hexan/EtOAc, 10:1); white solid; ^{1}H NMR (400 MHz, $CDCl_3$) δ 8.56 (s, 1H), 8.15-8.13 (m, 2H), 7.83 (d, J = 6.8 Hz, 2H), 7.61 (t, J = 7.2 Hz, 1H), 7.51-7.44 (m, 5H); ^{13}C NMR (100 MHz, $CDCl_3$) δ 163.9, 156.8, 133.4, 131.8, 130.1, 129.7, 128.9 (2C), 128.7 (2C),128.6 (2C), 128.5 (2C); HRMS (ESI) m/z $(M+H)^+$ calcd for $C_{14}H_{12}NO_2{}^+$ = 226.0868; found 226.0865.

(*E*)-Benzaldehyde O-(4-methoxybenzoyl) oxime (5b)

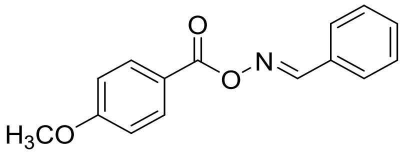

5b was obtained in 95% yield (242.2 mg) according to the general procedure (Hexan/EtOAc, 5:1); white solid; ^{1}H NMR (400 MHz, $CDCl_3$) δ 8.54 (s, 1H), 8.11-8.07 (m, 2H), 7.83-7.79 (m, 2H), 7.51-7.42 (m, 3H), 6.99-6.95 (m, 2H), 3.88 (s, 3H); ^{13}C NMR (100 MHz, $CDCl_3$) δ 163.8, 163.7, 156.4, 131.8 (2C), 131.7, 130.3, 128.9 (2C), 128.5 (2C), 120.9, 113.9 (2C), 55.5; HRMS (ESI) m/z $(M+H)^+$ calcd for $C_{15}H_{14}NO_3{}^+$ = 256.0974; found 256.0971.

(*E*)-Benzaldehyde O-(4-methylbenzoyl) oxime (5c)

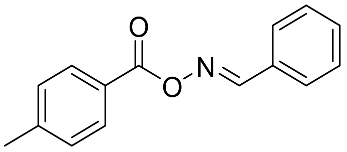

5c was obtained in 96% yield (229.4 mg) according to the general procedure (Hexan/EtOAc, 10:1); white solid; ^{1}H NMR (400 MHz, $CDCl_3$) δ 8.55 (s, 1H), 8.04 (d, J = 8.4 Hz, 2H), 7.82-7.80 (m, 2H), 7.51-7.43 (m, 3H), 7.30 (d, J = 8.0 Hz, 2H), 2.44 (s, 3H); ^{13}C NMR (100 MHz,

CDCl$_3$) δ 164.0, 156.6, 144.2, 131.7, 130.3, 129.8 (2C), 129.3 (2C), 128.9 (2C), 128.5 (2C), 125.9, 21.7; HRMS (ESI) m/z (M+H)$^+$ calcd for $C_{15}H_{14}NO_2{}^+$ = 240.1025; found 240.1023.

(*E*)-Benzaldehyde O-(2,4,6-trimethylbenzoyl) oxime (5d)

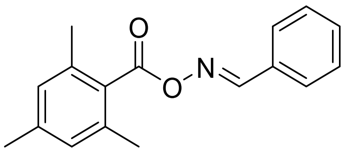

5d was obtained in 90% yield (240.3 mg) according to the general procedure (Hexan/EtOAc, 10:1); yellow oil; ^{1}H NMR (400 MHz, CDCl$_3$) δ 8.43 (s, 1H), 7.80-7.76 (m, 2H), 7.52-7.43 (m, 3H), 6.90 (s, 2H), 2.38 (s, 6H), 2.31 (s, 3H); ^{13}C NMR (100 MHz, CDCl$_3$) δ 167.4, 156.6, 140.0, 135.9 (2C), 131.8 (2C), 130.1, 128.9, 128.8, 128.5 (2C), 128.4 (2C), 21.2, 19.8 (2C); HRMS (ESI) m/z (M+H)$^+$ calcd for $C_{17}H_{18}NO_2{}^+$ = 268.1338; found 268.1335.

(*E*)-Benzaldehyde O-(4-chlorobenzoyl) oxime (5e)

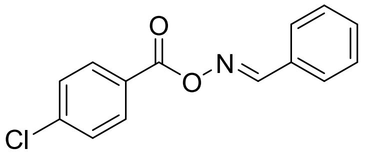

5e was obtained in 87% yield (225.3 mg) according to the general procedure (Hexan/EtOAc, 10:1); white solid; ^{1}H NMR (400 MHz, CDCl$_3$) δ 8.55 (s, 1H), 8.09-8.05 (m, 2H), 7.81-7.79 (m, 2H), 7.53-7.43 (m, 5H); ^{13}C NMR (100 MHz, CDCl$_3$) δ 163.1, 157.0, 139.9, 131.9, 131.1 (2C), 129.9, 128.9 (4C), 128.5 (2C), 127.1; HRMS (ESI) m/z (M+H)$^+$ calcd for $C_{14}H_{11}ClNO_2{}^+$ = 260.0478; found 260.0477.

(*E*)-Benzaldehyde O-(4-(trifluoromethyl)benzoyl) oxime (5f)

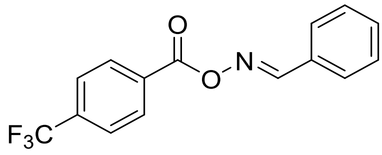

5f was obtained in 82% yield (240.2 mg) according to the general procedure (Hexan/EtOAc, 10:1); white solid; ^{1}H NMR (400 MHz, $CDCl_3$) δ 8.59 (s, 1H), 8.38-8.32 (m, 2H), 7.88-7.81 (m, 3H), 7.65 (t, J = 7.6 Hz, 1H), 7.53-7.44 (m, 3H); ^{13}C NMR (100 MHz, $CDCl_3$) δ 162.7, 157.4, 132.9, 132.0, 131.5 (q, J = 32.8 Hz), 129.9 (q, J = 3.6 Hz), 129.8, 129.6, 129.3, 129.0 (2C), 128.6 (2C), 126.6 (q, J = 3.7 Hz), 125.0 (q, J = 270.4 Hz); HRMS (ESI) m/z $(M+H)^+$ calcd for $C_{15}H_{11}F_3NO_2^+$ = 294.0742; found 294.0740.

(*E*)-Benzaldehyde O-(4-nitrobenzoyl) oxime (5g)

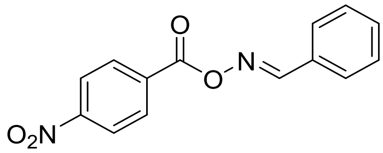

5g was obtained in 77% yield (207.9 mg) according to the general procedure (Hexan/EtOAc, 5:1); white solid; ^{1}H NMR (400 MHz, DMSO-d_6) δ 10.6 (s, 1H), 8.39-8.37 (m, 2H), 8.21-8.18 (m, 2H), 7.80 (d, J = 7.6 Hz, 2H), 7.39 (t, J = 7.6 Hz, 2H), 7.15 (t, J = 7.6 Hz, 1H); ^{13}C NMR (100 MHz, DMSO-d_6) δ 164.4, 149.6, 141.1, 139.2, 129.7 (2C), 129.2 (2C), 124.6, 124.0 (2C), 120.9 (2C); HRMS (ESI) m/z $(M+H)^+$ calcd for $C_{14}H_{11}N_2O_4^+$ = 271.0719; found 271.0718.

(*E*)-Benzaldehyde O-(3-bromobenzoyl) oxime (5h)

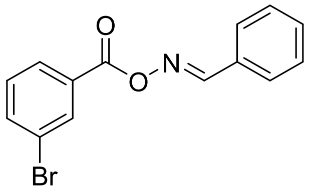

5h was obtained in 90% yield (273.6 mg) according to the general procedure (Hexan/EtOAc, 10:1); white solid; ^{1}H NMR (400 MHz, CDCl$_3$) δ 8.56 (s, 1H), 8.27 (t, J = 1.6 Hz, 1H), 8.08-8.06 (m, 1H), 7.82-7.80 (m, 2H), 7.76-7.73 (m, 1H), 7.51-7.44 (m, 3H), 7.38 (t, J = 8.0 Hz, 1H); ^{13}C NMR (100 MHz, CDCl$_3$) δ 162.6, 157.2, 136.4, 132.6, 132.0, 130.6, 130.2, 129.9, 129.0 (2C), 128.6 (2C), 128.3, 122.7; HRMS (ESI) m/z (M+H)$^+$ calcd for $C_{14}H_{11}BrNO_2{}^+$ = 303.9973; found 303.9971.

(*E*)-Benzaldehyde O-(1-naphthoyl) oxime (5i)

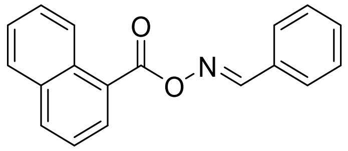

5i was obtained in 84% yield (231.0 mg) according to the general procedure (Hexan/EtOAc, 10:1); yellow oil; ^{1}H NMR (400 MHz, CDCl$_3$) δ 8.90 (d, J = 8.4 Hz, 1H), 8.58 (s, 1H), 8.24 (dd, J = 7.2, 1.6 Hz, 1H), 8.08 (d, J = 8.4 Hz, 1H), 7.92 (d, J = 8.0 Hz, 1H), 7.85-7.83 (m, 2H), 7.67-7.64 (m, 1H), 7.59-7.45 (m, 5H); ^{13}C NMR (100 MHz, CDCl$_3$) δ 164.9, 156.9, 133.9, 133.7, 131.8, 131.4, 130.2, 129.9, 128.9 (2C), 128.6, 128.5 (2C), 128.0, 126.5, 125.7, 124.5 (2C); HRMS (ESI) m/z (M+H)$^+$ calcd for $C_{18}H_{14}NO_2{}^+$ = 276.1025; found 276.1022.

(*E*)-Benzaldehyde O-([1,1'-biphenyl]-4-carbonyl) oxime (5j)

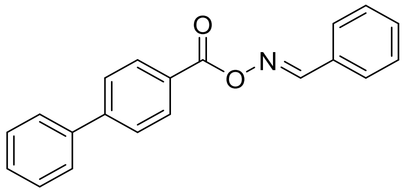

5j was obtained in 81% yield (243.8 mg) according to the general procedure (Hexan/EtOAc, 10:1); white solid; ^{1}H NMR (400 MHz, $CDCl_3$) δ 8.59 (s, 1H), 8.22-8.20 (m, 2H), 7.84-7.82 (m, 2H), 7.73-7.71 (m, 2H), 7.66-7.64 (m, 2H), 7.53-7.40 (m, 6H); ^{13}C NMR (100 MHz, $CDCl_3$) δ 163.9, 156.8, 146.2, 139.9, 131.8, 130.3 (2C), 130.2 (2C), 129.0 (2C), 128.9 (2C), 128.5 (2C), 128.3, 127.3 (2C), 127.2 (2C); HRMS (ESI) m/z $(M+H)^+$ calcd for $C_{20}H_{16}NO_2^+$ = 302.1181; found 302.1179.

(*E*)-Benzaldehyde O-(3,3-dimethylbutanoyl) oxime (5k)

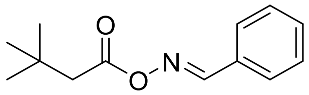

5k was obtained in 95% yield (208.1 mg) according to the general procedure (Hexan/EtOAc, 10:1); yellow oil; ^{1}H NMR (400 MHz, $CDCl_3$) δ 8.35 (s, 1H), 7.75-7.73 (m, 2H), 7.49-7.40 (m, 3H), 2.35 (s, 2H), 1.11 (s, 9H); ^{13}C NMR (100 MHz, $CDCl_3$) δ 169.4, 155.9, 131.6, 130.3, 128.9 (2C), 128.4 (2C), 46.3, 31.1, 29.7 (3C); HRMS (ESI) m/z $(M+H)^+$ calcd for $C_{13}H_{18}NO_2^+$ = 220.1338; found 220.1336.

(*E*)-Benzaldehyde O-(2-ethylhexanoyl) oxime (5l)

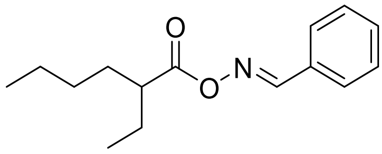

5l was obtained in 94% yield (232.2 mg) according to the general procedure (Hexan/EtOAc, 10:1); yellow oil; ^{1}H NMR (400 MHz, $CDCl_3$) δ 8.38 (s, 1H), 7.76-7.74 (m, 2H), 7.49-7.40 (m, 3H), 2.46-2.39 (m, 1H), 1.79-1.53 (m, 4H), 1.38-1.29 (m, 4H), 1.00-0.88 (m, 6H); ^{13}C NMR (100 MHz, $CDCl_3$) δ 173.6, 156.1, 131.6, 130.3, 128.9 (2C), 128.4 (2C), 46.1, 31.8, 29.6, 25.6, 22.6, 13.9, 11.9; HRMS (ESI) m/z $(M+H)^+$ calcd for $C_{15}H_{22}NO_2^+$ = 248.1651; found 248.1650.

(*E*)-Benzaldehyde O-undecanoyl oxime (5m)

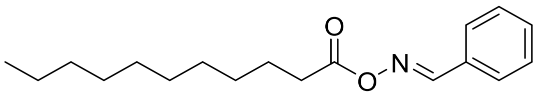

5m was obtained in 92% yield (265.9 mg) according to the general procedure (Hexan/EtOAc, 10:1); white solid; ^{1}H NMR (400 MHz, $CDCl_3$) δ 8.35 (s, 1H), 7.75-7.73 (m, 2H), 7.49-7.40 (m, 3H), 2.47 (t, J = 7.6 Hz, 2H), 1.77-1.70 (m, 2H), 1.41-1.27 (m, 14H), 0.88 (t, J = 6.8 Hz, 3H); ^{13}C NMR (100 MHz, $CDCl_3$) δ 171.3, 155.9, 131.6, 130.3, 128.9 (2C), 128.4 (2C), 32.9, 31.9, 29.5, 29.4, 29.3, 29.2, 29.1, 24.9, 22.7, 14.1; HRMS (ESI) m/z $(M+H)^+$ calcd for $C_{18}H_{28}NO_2^+$ = 290.2120; found 290.2118.

(*E*)-Benzaldehyde O-cyclohexanecarbonyl oxime (5n)

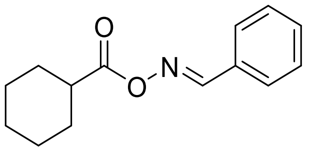

5n was obtained in 94% yield (217.1 mg) according to the general procedure (Hexan/EtOAc, 10:1); white solid; ^{1}H NMR (400 MHz, $CDCl_3$) δ 8.36 (s, 1H), 7.74-7.72 (m, 2H), 7.48-7.39 (m, 3H), 2.51-2.44 (m, 1H), 2.01-1.94 (m, 2H), 1.83-1.79 (m, 2H), 1.69-1.53 (m, 3H), 1.37-

1.27 (m, 3H); ^{13}C NMR (100 MHz, $CDCl_3$) δ 173.2, 156.1, 131.6, 130.3, 128.9 (2C), 128.4 (2C), 42.1, 28.9 (2C), 25.7, 25.4 (2C); HRMS (ESI) m/z $(M+H)^+$ calcd for $C_{14}H_{18}NO_2^+$ = 232.1338; found 232.1337.

(*E*)-N,1-Diphenylmethanimine (6a)

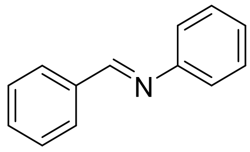

6a was obtained in 97% yield (175.6 mg) according to the general procedure (Hexan/EtOAc, 10:1); yellow solid; ^1H NMR (400 MHz, $CDCl_3$) δ 8.502 (s, 1H), 7.97-7.95 (m, 2H), 7.52-7.43 (m, 5H), 7.30-7.26 (m, 3H); ^{13}C NMR (100 MHz, $CDCl_3$) δ 160.4, 152.1, 136.3, 131.4, 129.2 (2C), 128.9 (2C), 128.8 (2C), 126.0, 120.9 (2C); HRMS (ESI) m/z $(M+H)^+$ calcd for $C_{13}H_{12}N^+$ = 182.0970; found 182.0967.

^{1}H and ^{13}C NMR spectra of oxime esters

(*E*)-Benzaldehyde O-butyryl oxime (4a)

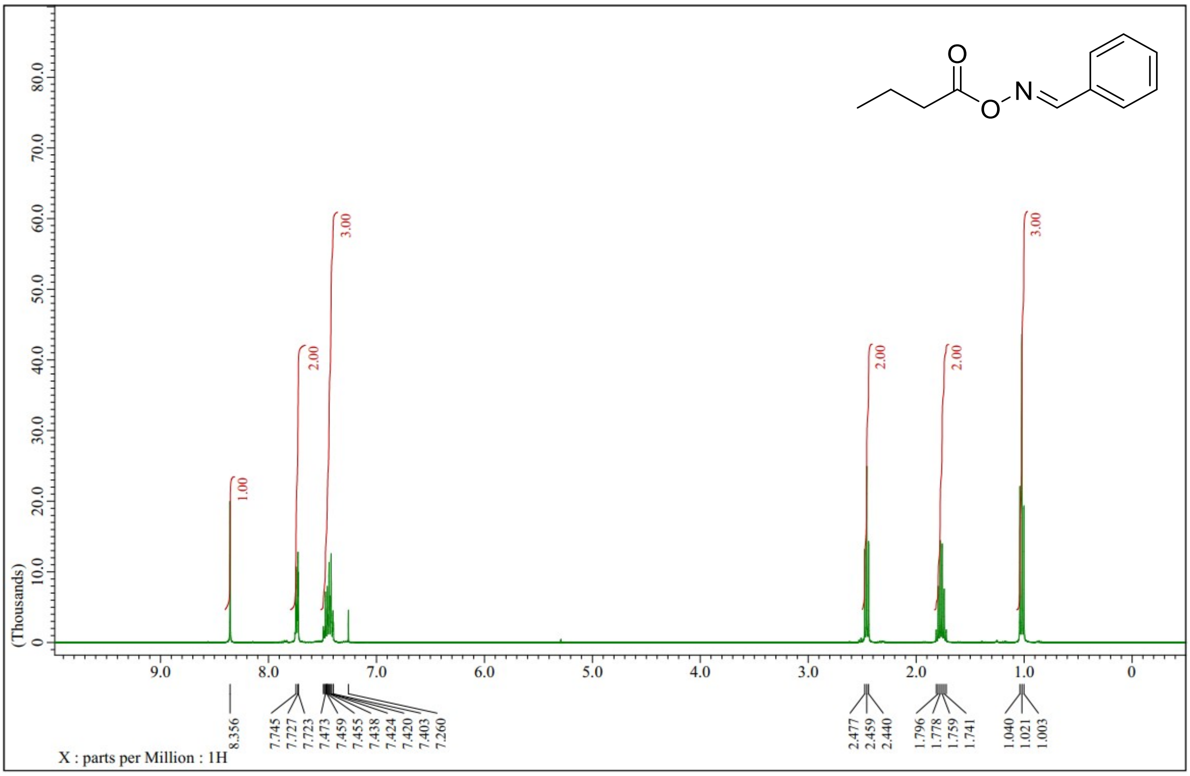

^{1}H NMR spectrum of (*E*)-benzaldehyde O-butyryl oxime (**4a**)

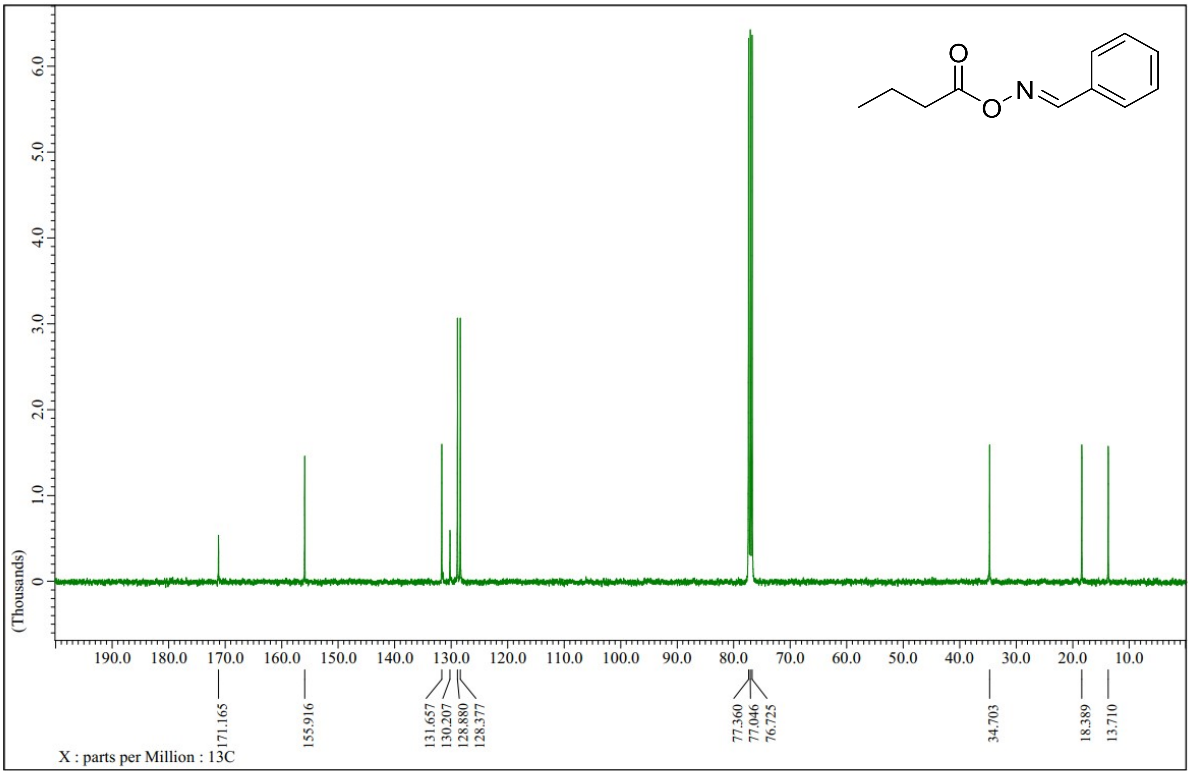

^{13}C NMR spectrum of (*E*)-benzaldehyde O-butyryl oxime (**4a**)

(*E*)-4-Methylbenzaldehyde O-butyryl oxime (4b)

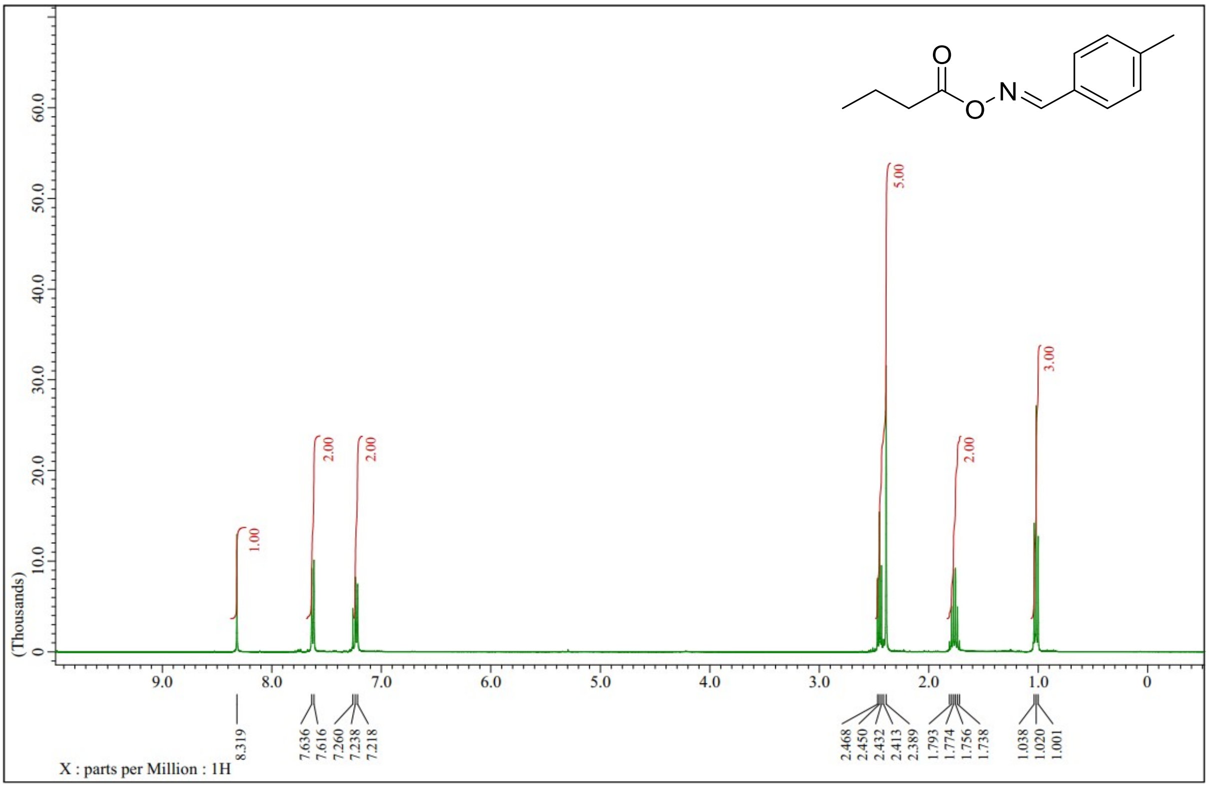

^{1}H NMR spectrum of (*E*)-4-methylbenzaldehyde O-butyryl oxime (**4b**)

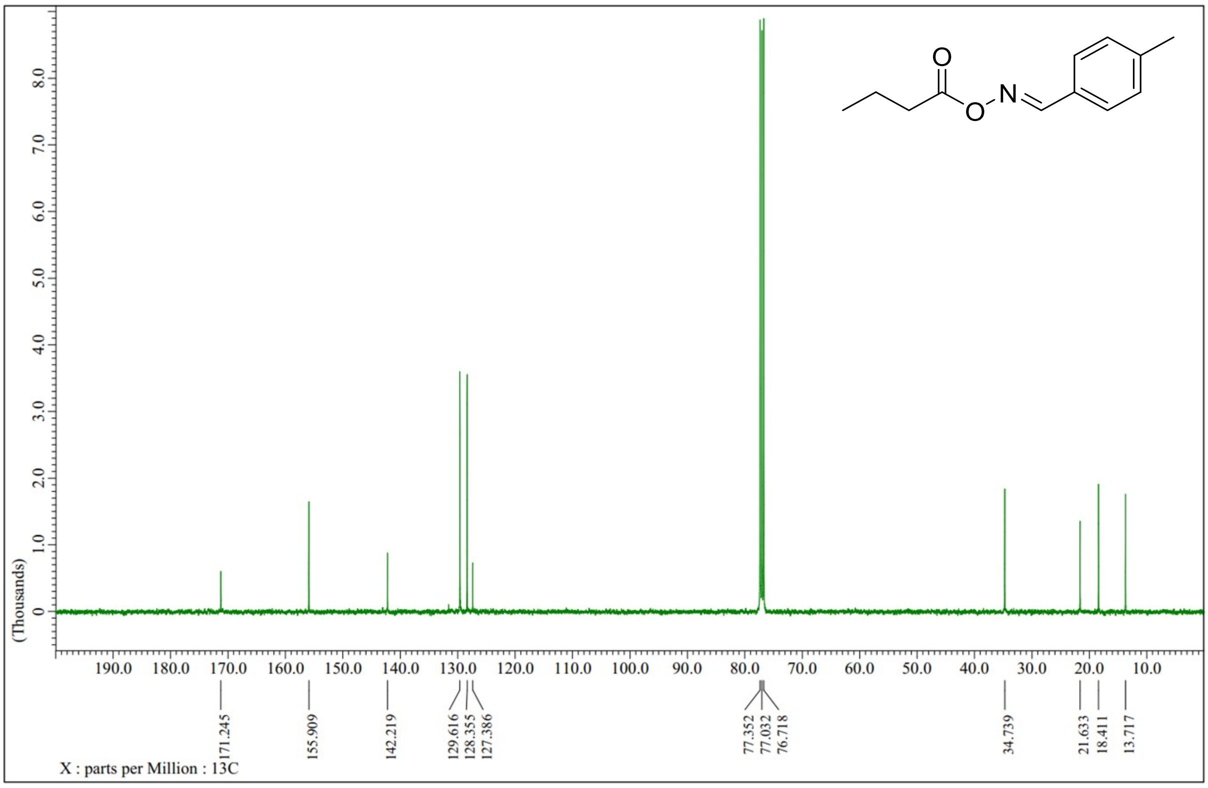

^{13}C NMR spectrum of (*E*)-4-methylbenzaldehyde O-butyryl oxime (**4b**)

(*E*)-2-Chlorobenzaldehyde O-butyryl oxime (4c)

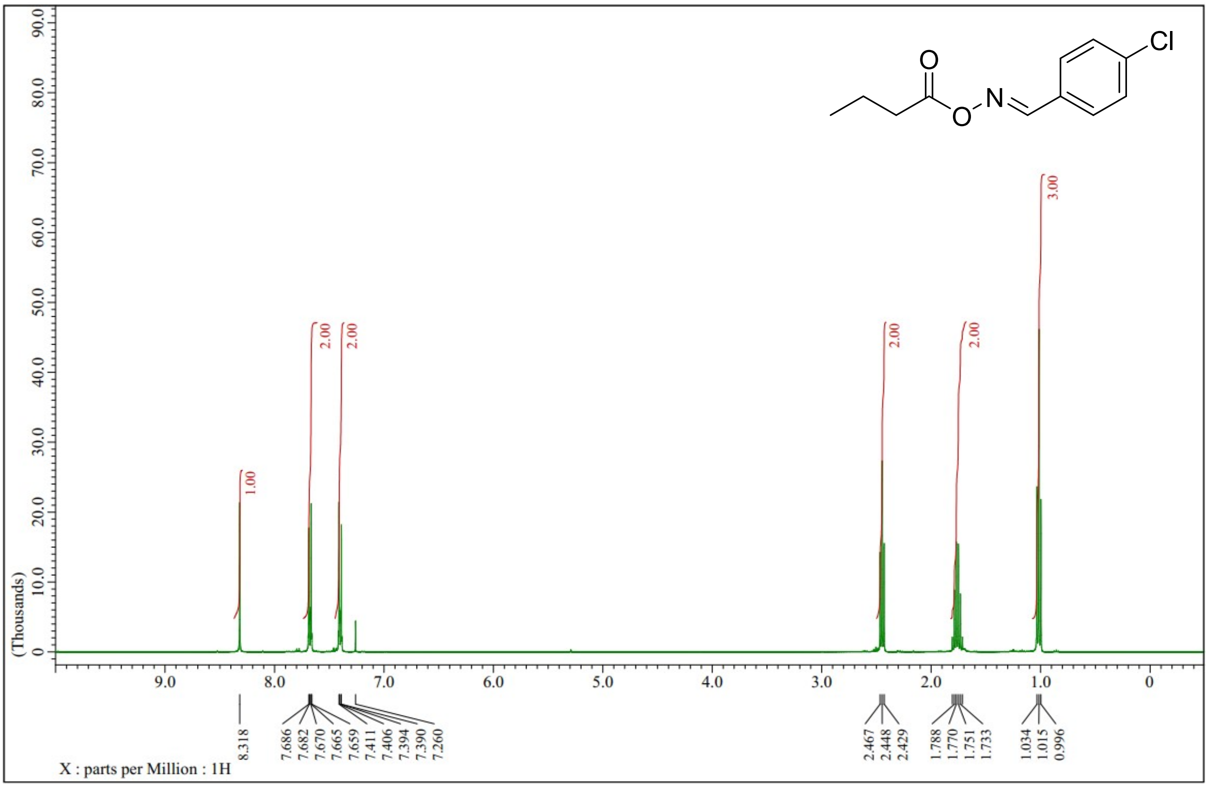

^{1}H NMR spectrum of (*E*)-2-chlorobenzaldehyde O-butyryl oxime (**4c**)

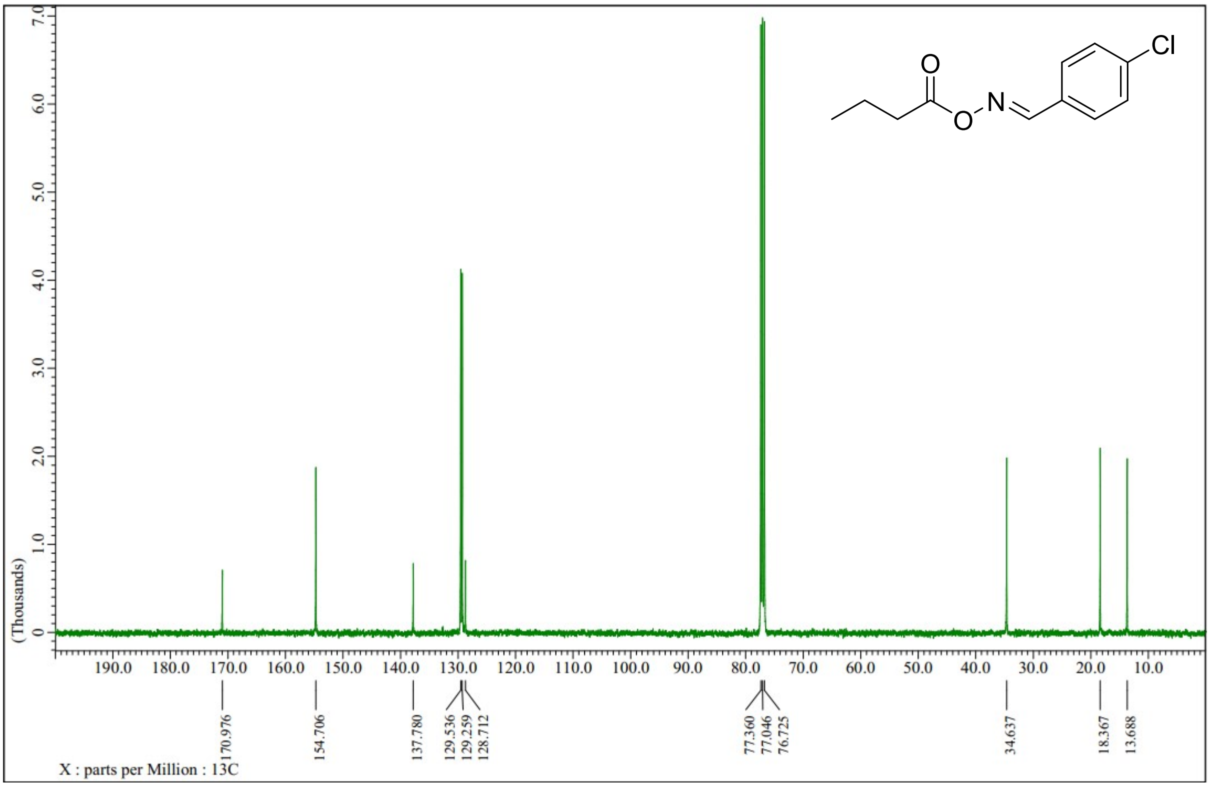

^{13}C NMR spectrum of (*E*)-2-chlorobenzaldehyde O-butyryl oxime (**4c**)

(*E*)-4-Nitrobenzaldehyde O-butyryl oxime (4d)

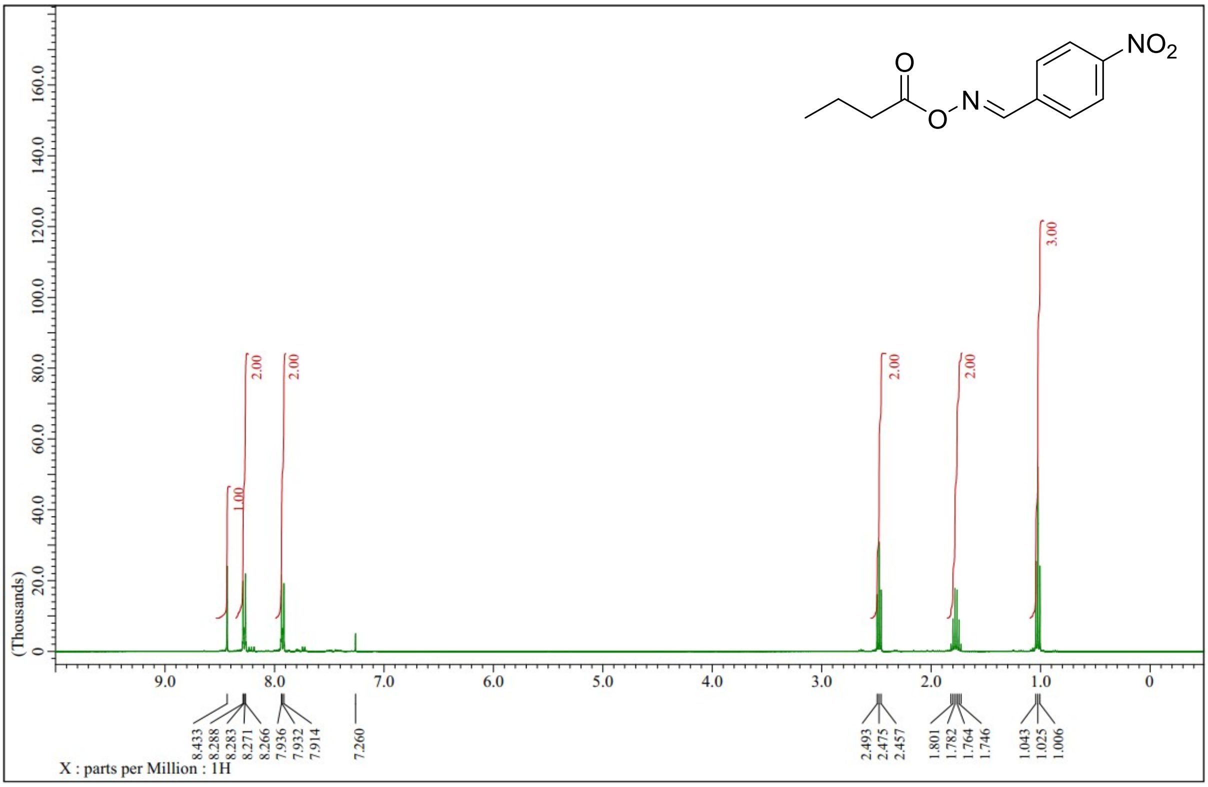

^{1}H NMR spectrum of (*E*)-4-nitrobenzaldehyde O-butyryl oxime (**4d**)

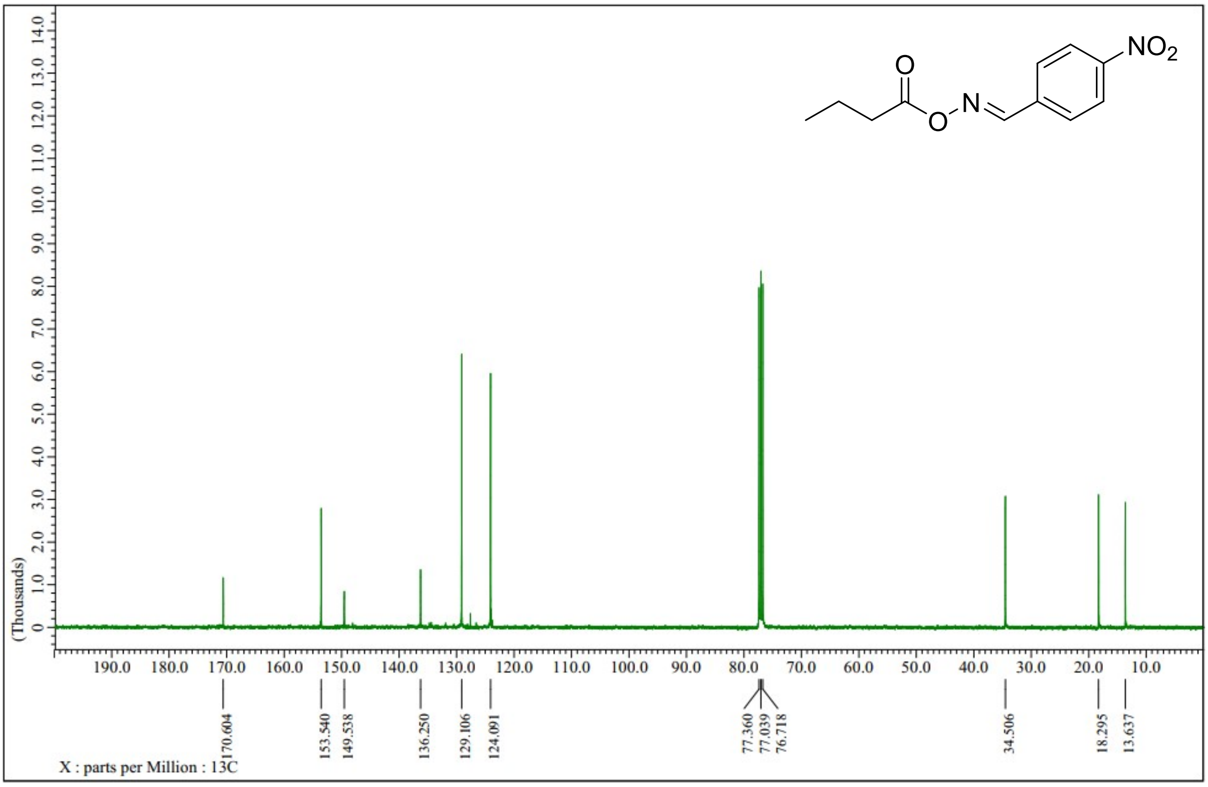

^{13}C NMR spectrum of (*E*)-4-nitrobenzaldehyde O-butyryl oxime (**4d**)

(*E*)-2-Methylbenzaldehyde O-butyryl oxime (4e)

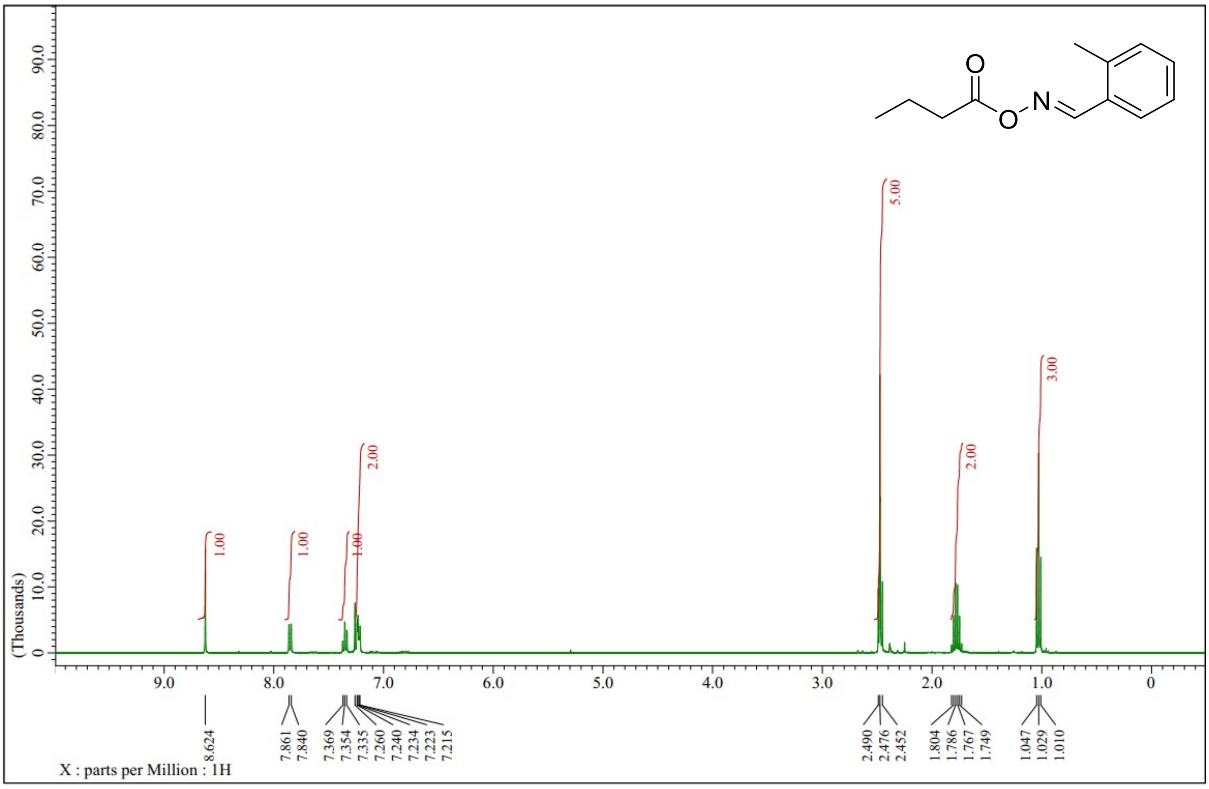

^{1}H NMR spectrum of (*E*)-2-methylbenzaldehyde O-butyryl oxime (**4e**)

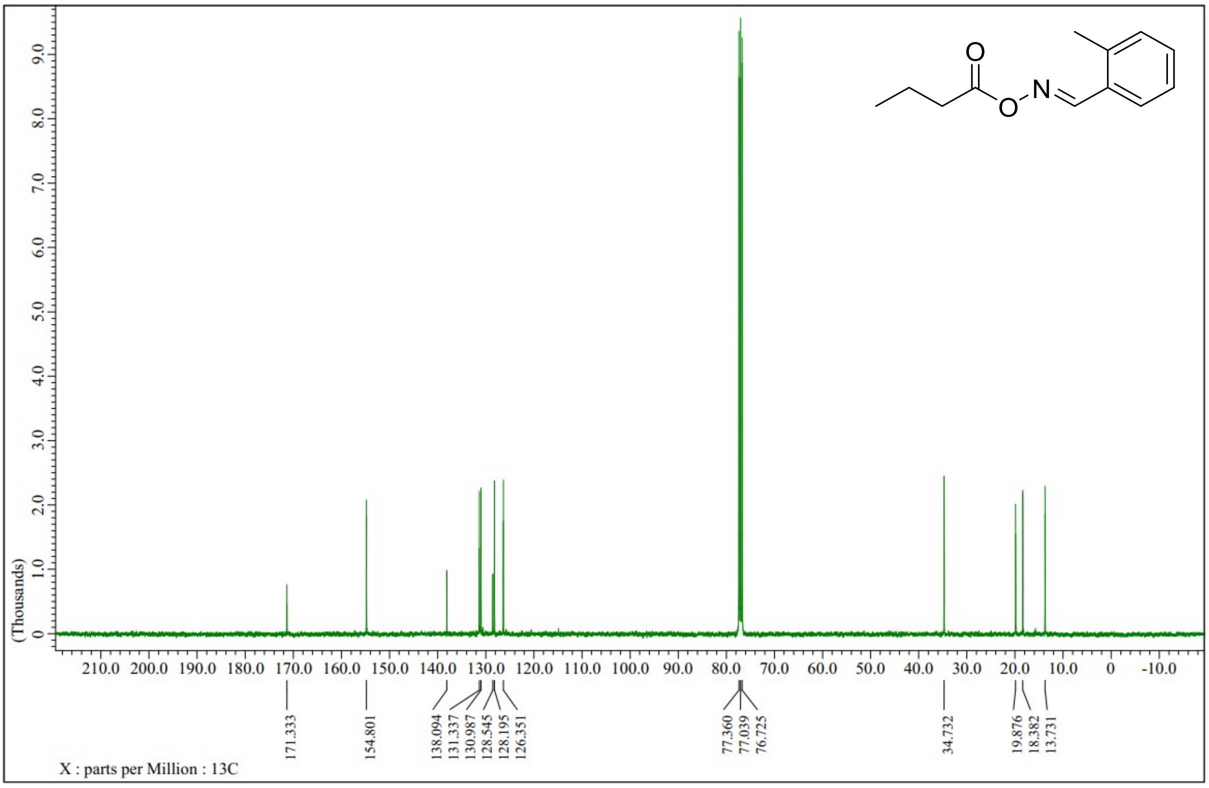

^{13}C NMR spectrum of (*E*)-2-methylbenzaldehyde O-butyryl oxime (**4e**)

(*E*)-2-Hydroxybenzaldehyde O-butyryl oxime (4f)

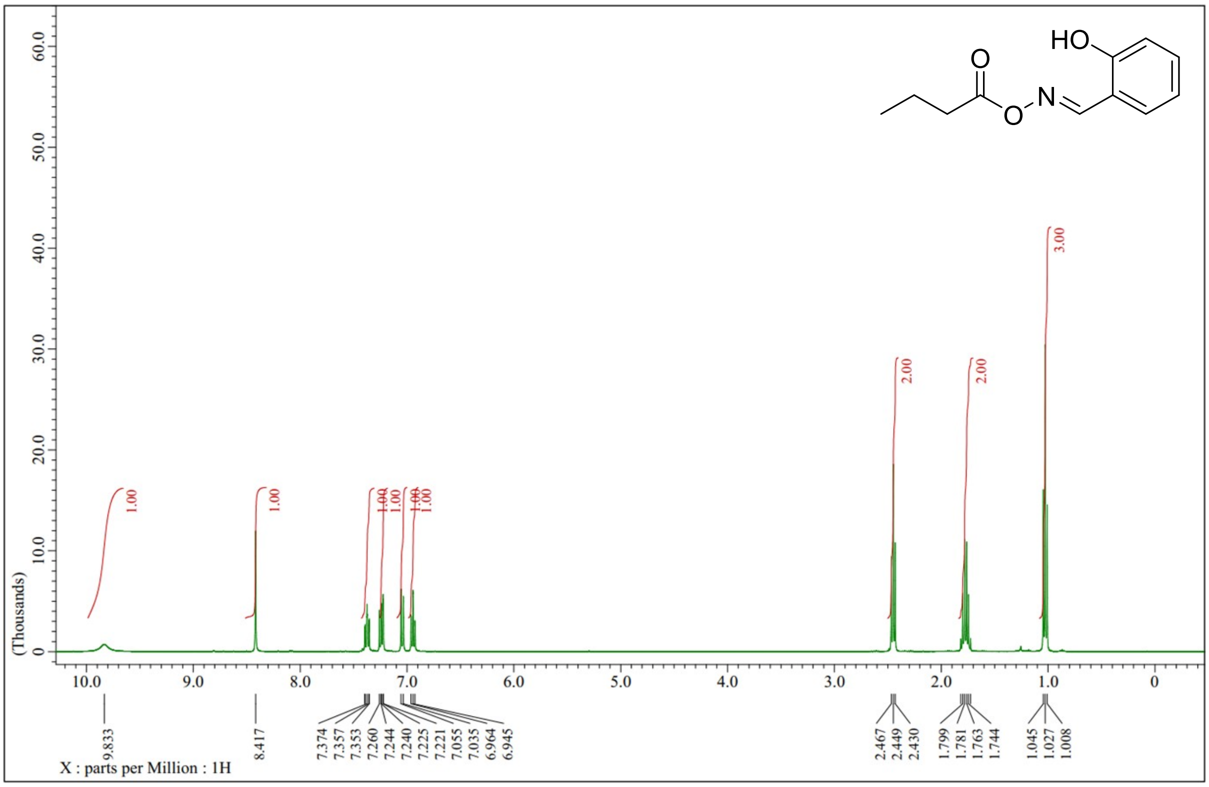

^{1}H NMR spectrum of (*E*)-2-hydroxybenzaldehyde O-butyryl oxime (**4f**)

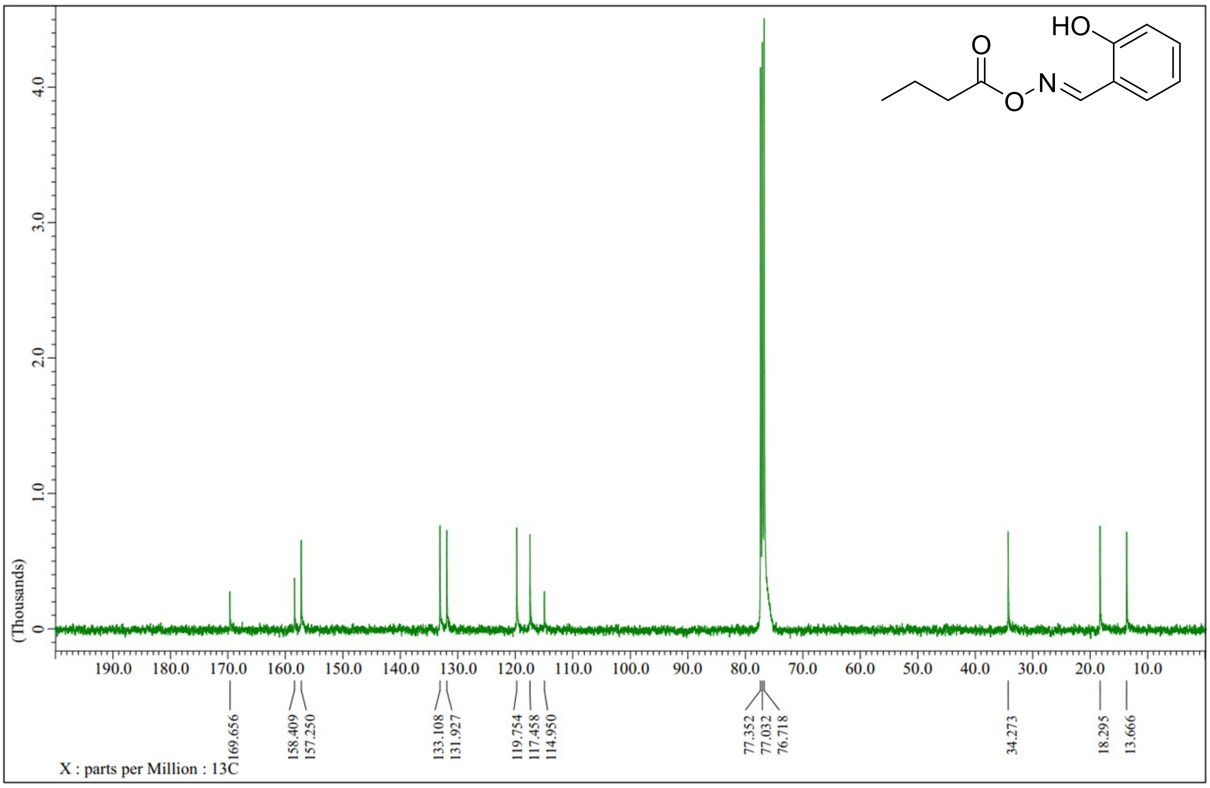

^{13}C NMR spectrum of (*E*)-2-hydroxybenzaldehyde O-butyryl oxime (**4f**)

(*E*)-2-Bromobenzaldehyde O-butyryl oxime (4g)

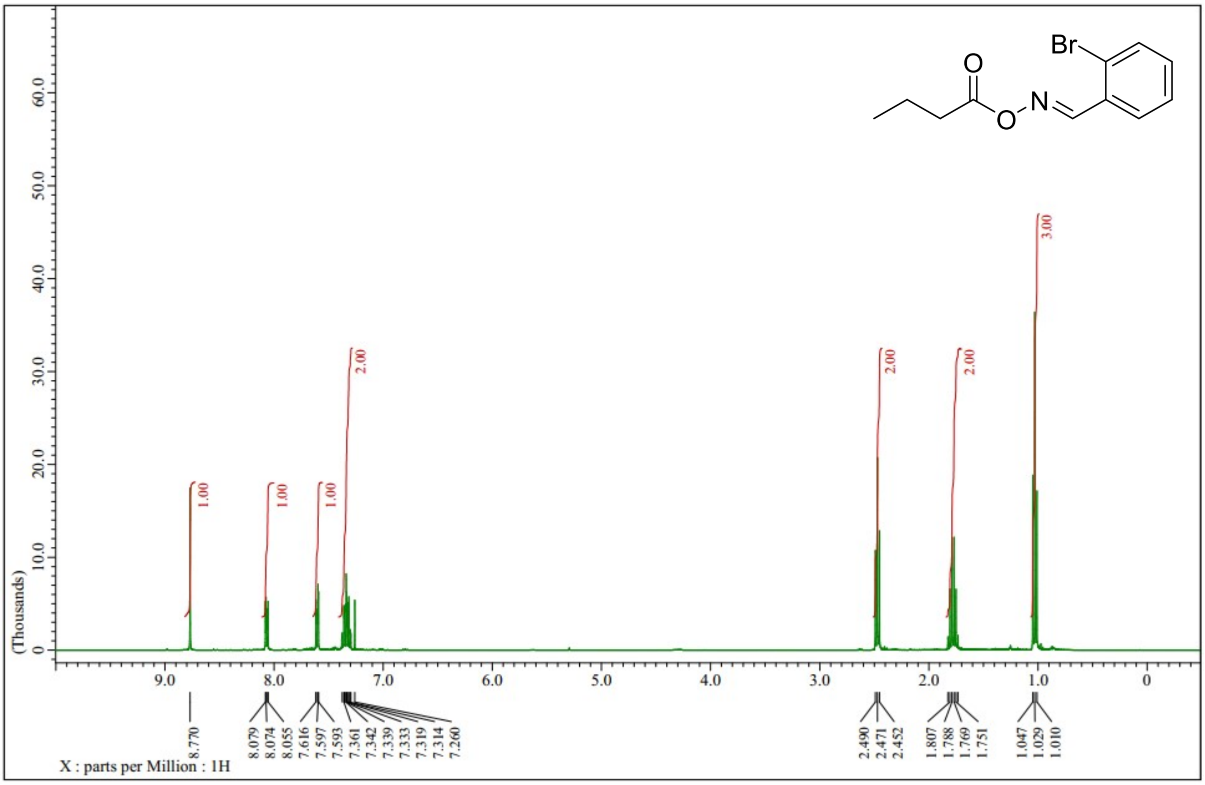

^{1}H NMR spectrum of (*E*)-2-bromobenzaldehyde O-butyryl oxime (**4g**)

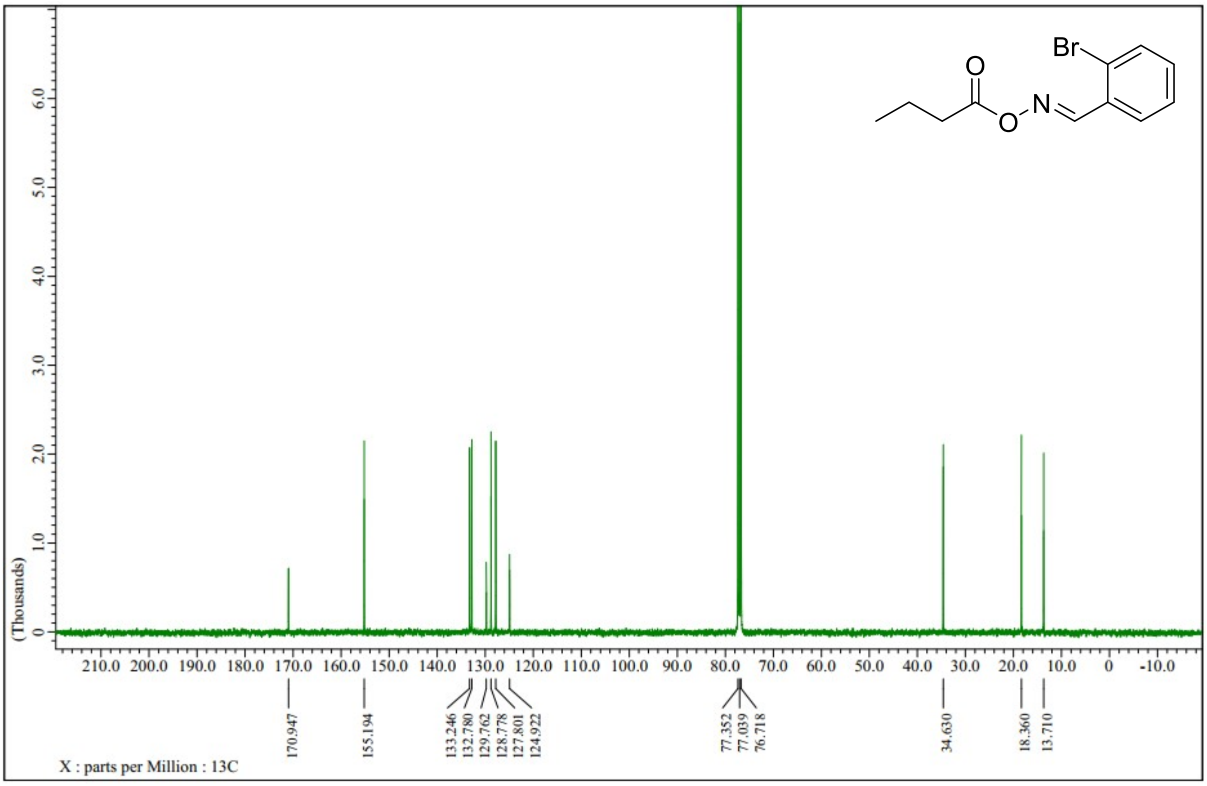

^{13}C NMR spectrum of (*E*)-2-bromobenzaldehyde O-butyryl oxime (**4g**)

(*E*)-1-Naphthaldehyde O-butyryl oxime (4h)

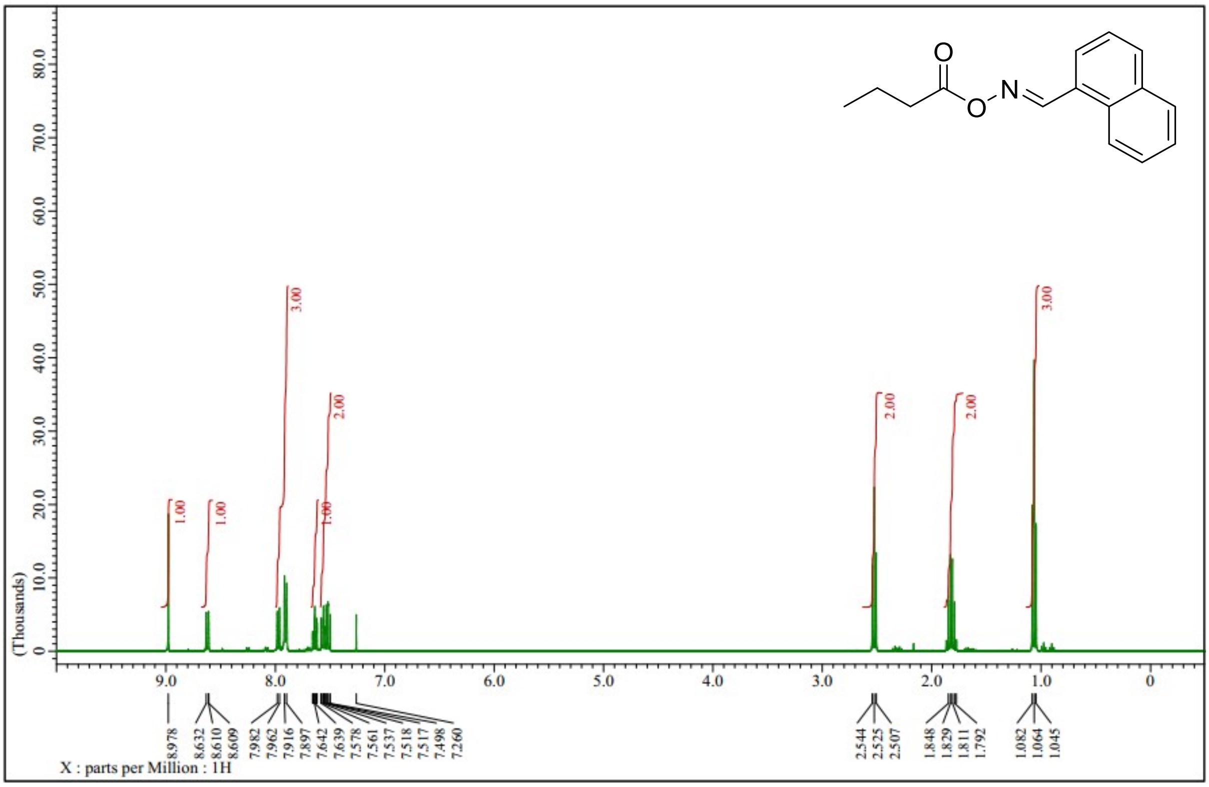

^{1}H NMR spectrum of (*E*)-1-naphthaldehyde O-butyryl oxime (**4h**)

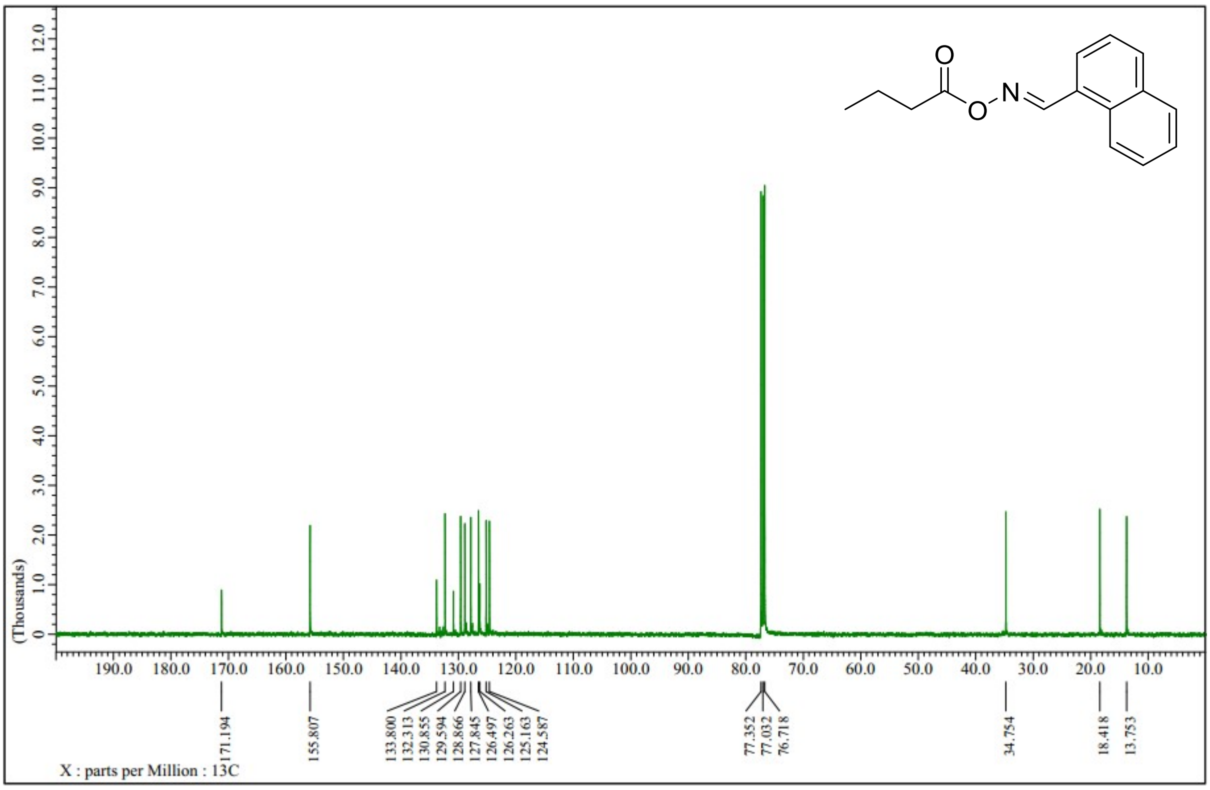

^{13}C NMR spectrum of (*E*)-1-naphthaldehyde O-butyryl oxime (**4h**)

(*E*)-Picolinaldehyde O-butyryl oxime (4i)

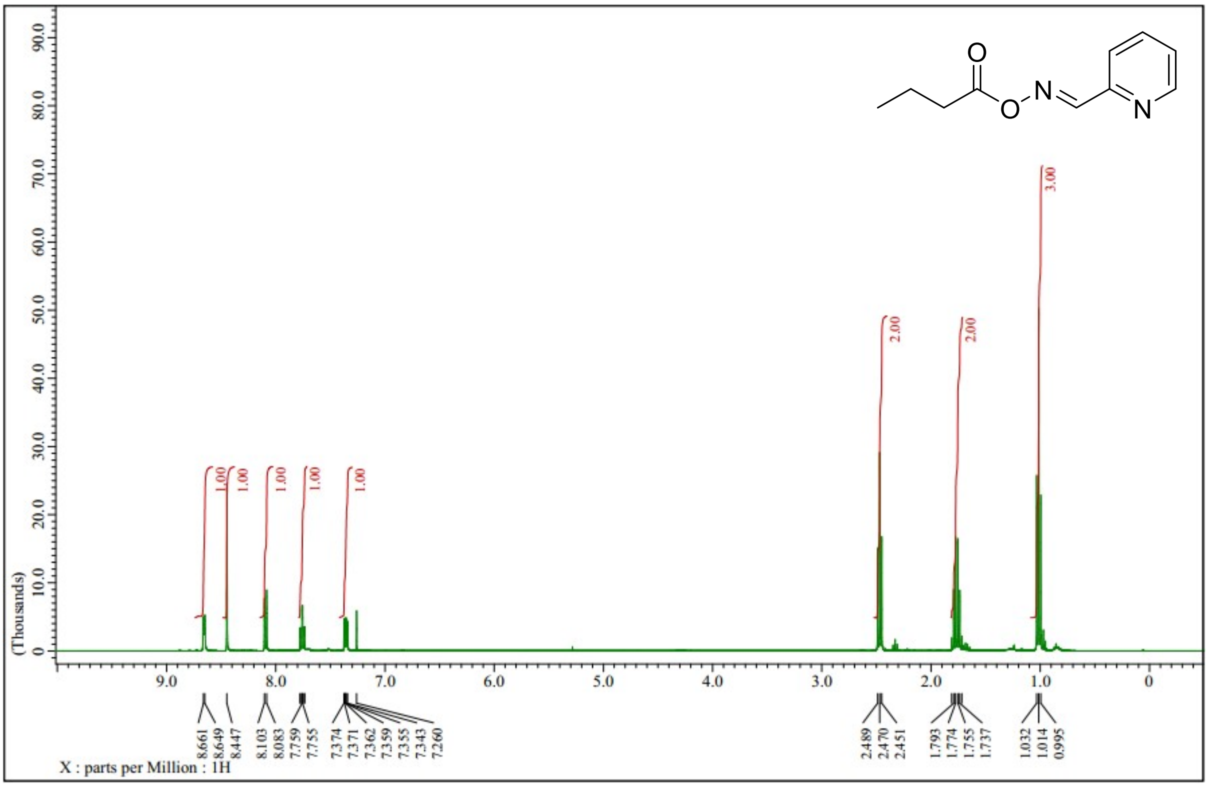

^{1}H NMR spectrum of (*E*)-picolinaldehyde O-butyryl oxime (**4i**)

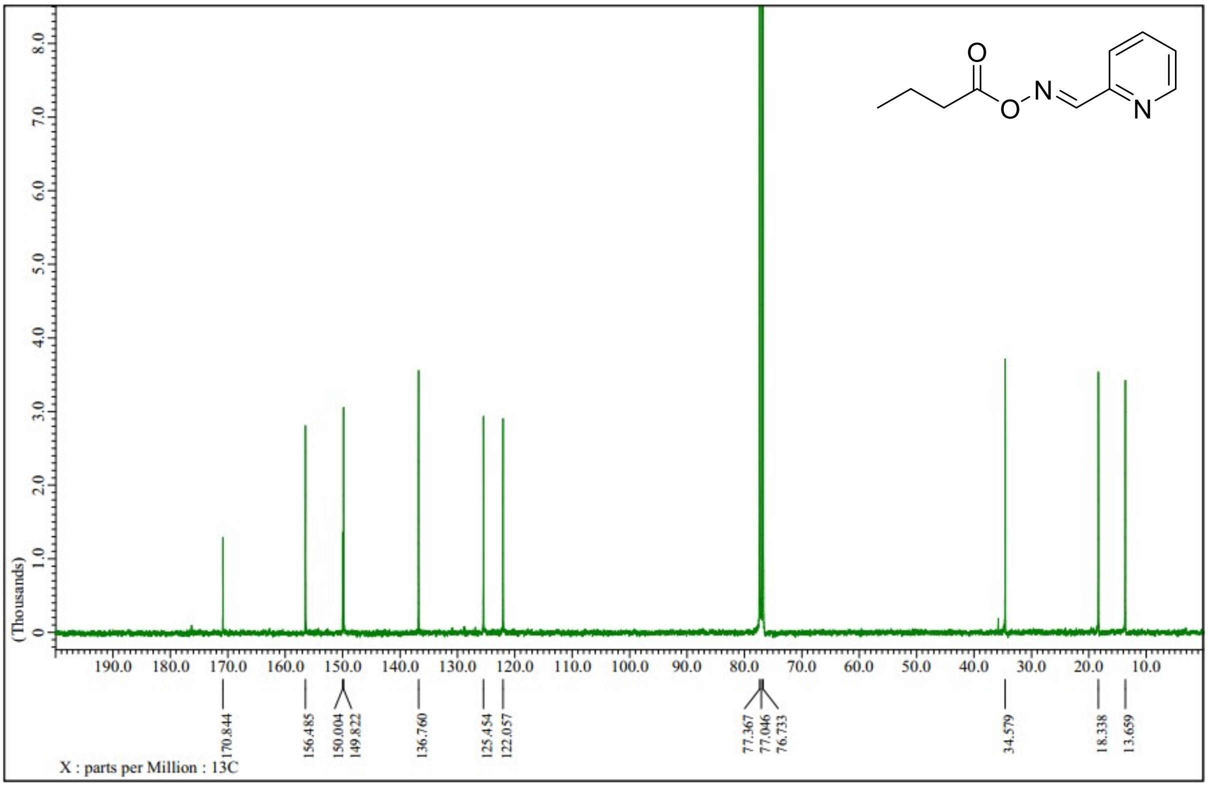

^{13}C NMR spectrum of (*E*)-picolinaldehyde O-butyryl oxime (**4i**)

(*E*)-Thiophene-2-carbaldehyde O-butyryl oxime (4j)

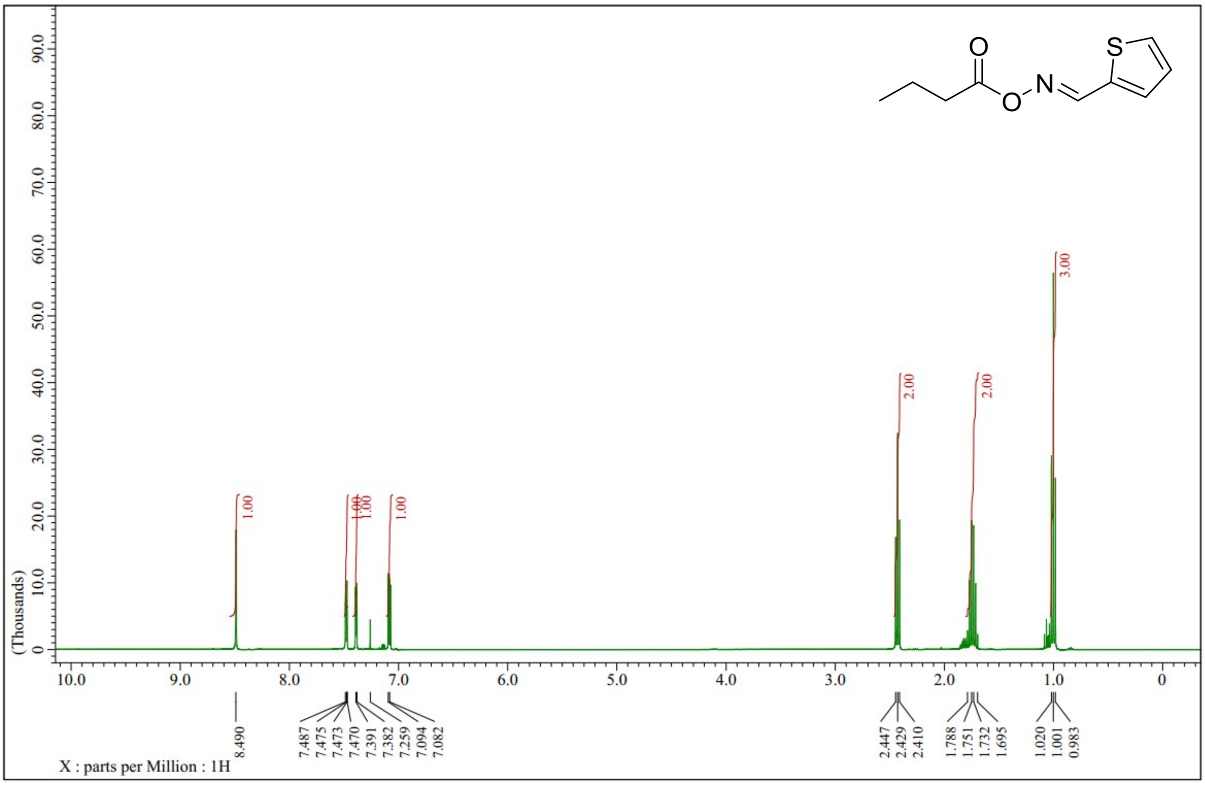

^{1}H NMR spectrum of (*E*)-thiophene-2-carbaldehyde O-butyryl oxime (**4j**)

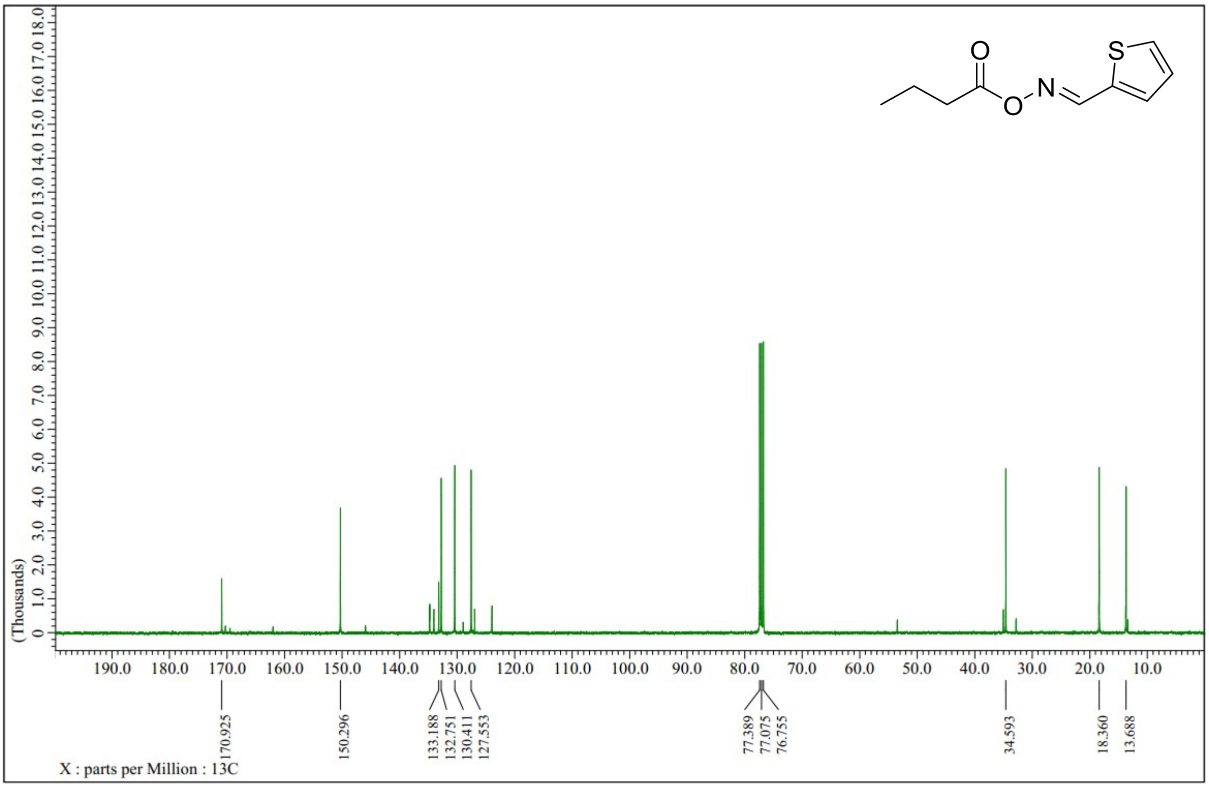

^{13}C NMR spectrum of (*E*)-thiophene-2-carbaldehyde O-butyryl oxime (**4j**)

(*E*)-Butyraldehyde O-butyryl oxime (4k)

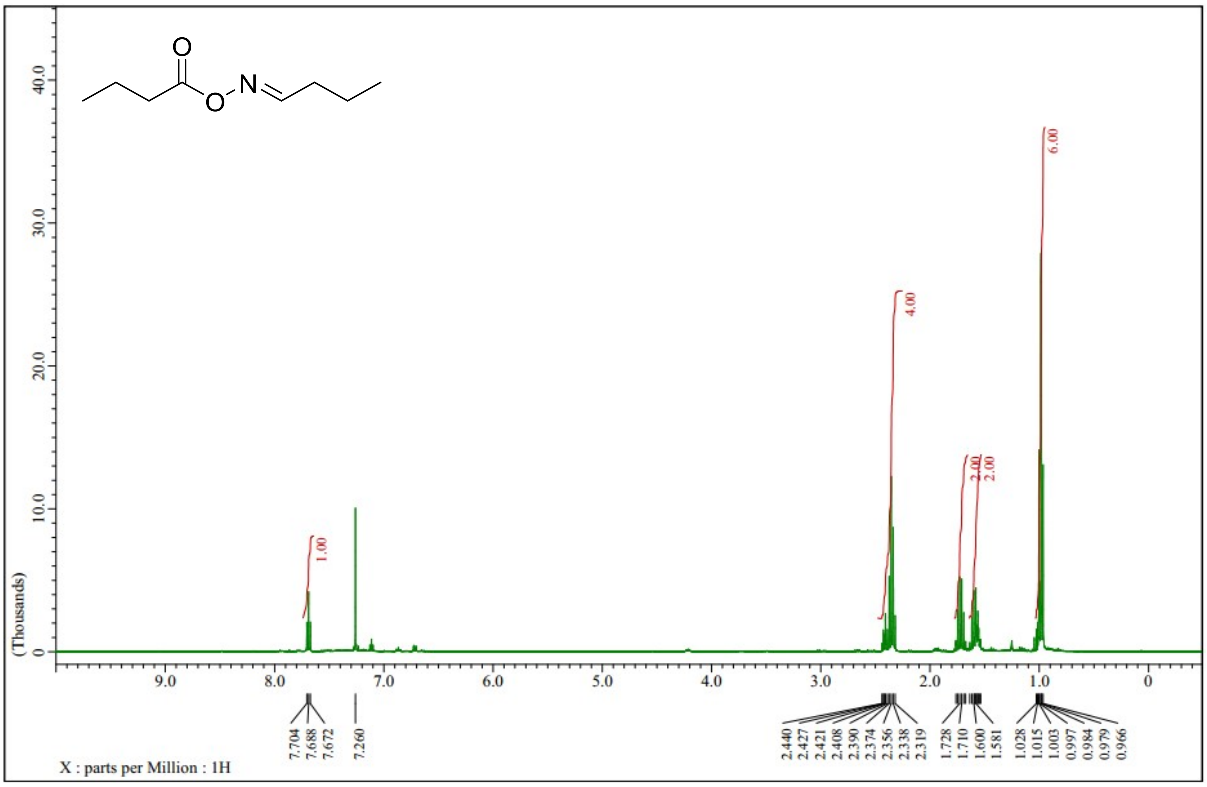

^{1}H NMR spectrum of (*E*)-butyraldehyde O-butyryl oxime (**4k**)

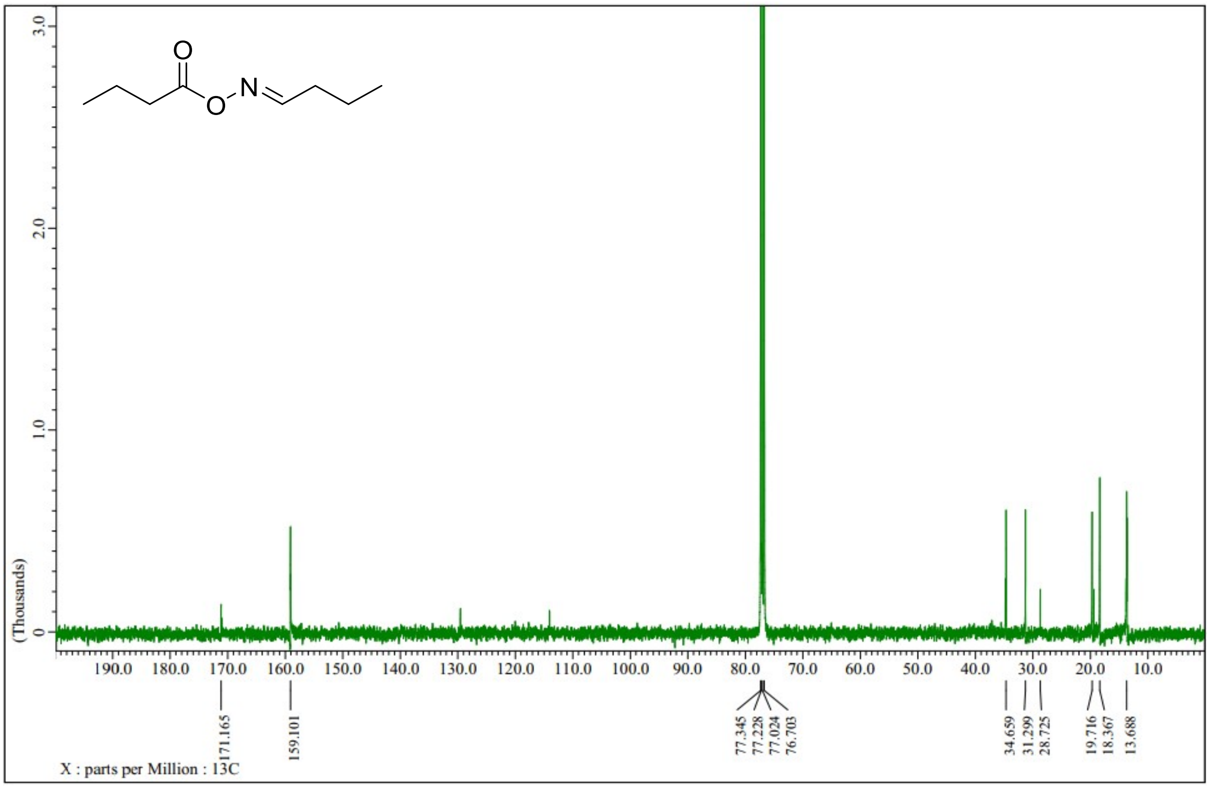

^{13}C NMR spectrum of (*E*)-butyraldehyde O-butyryl oxime (**4k**)

(*E*)-3-Methylbutanal O-butyryl oxime (4l)

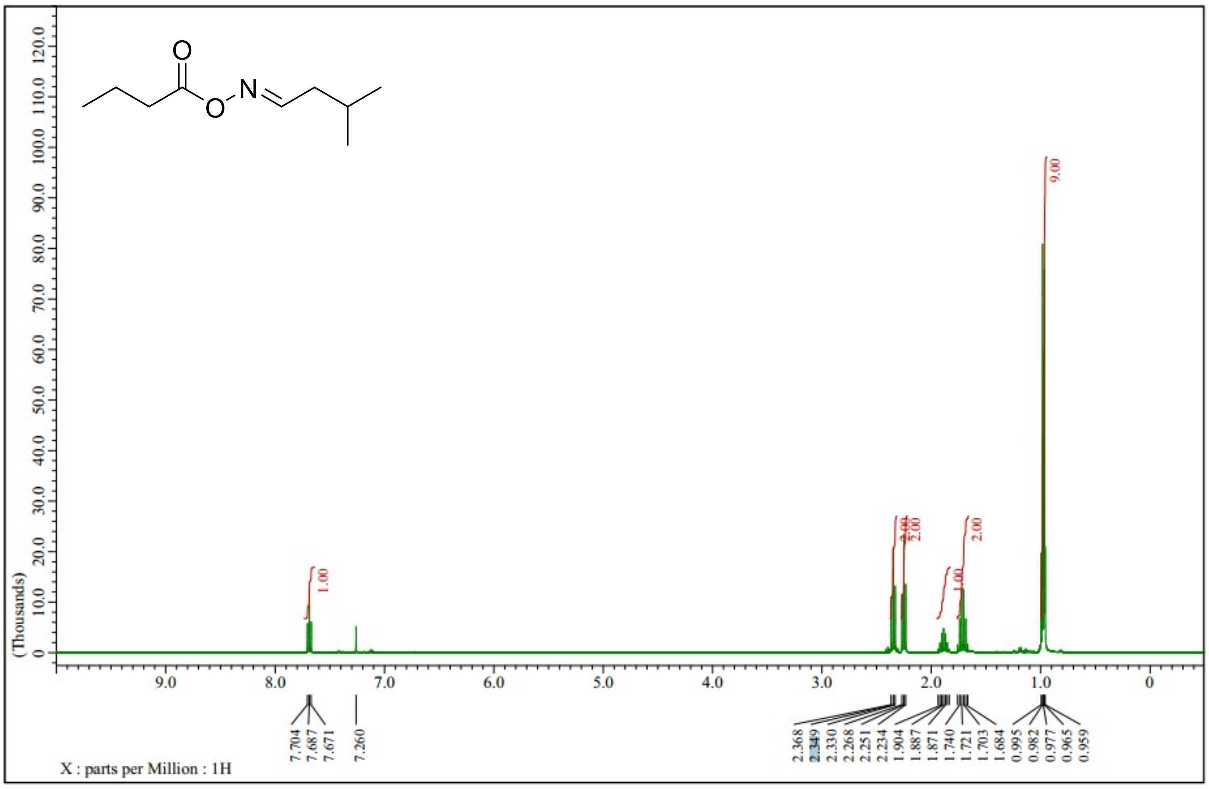

^{1}H NMR spectrum of (*E*)-3-methylbutanal O-butyryl oxime (**4l**)

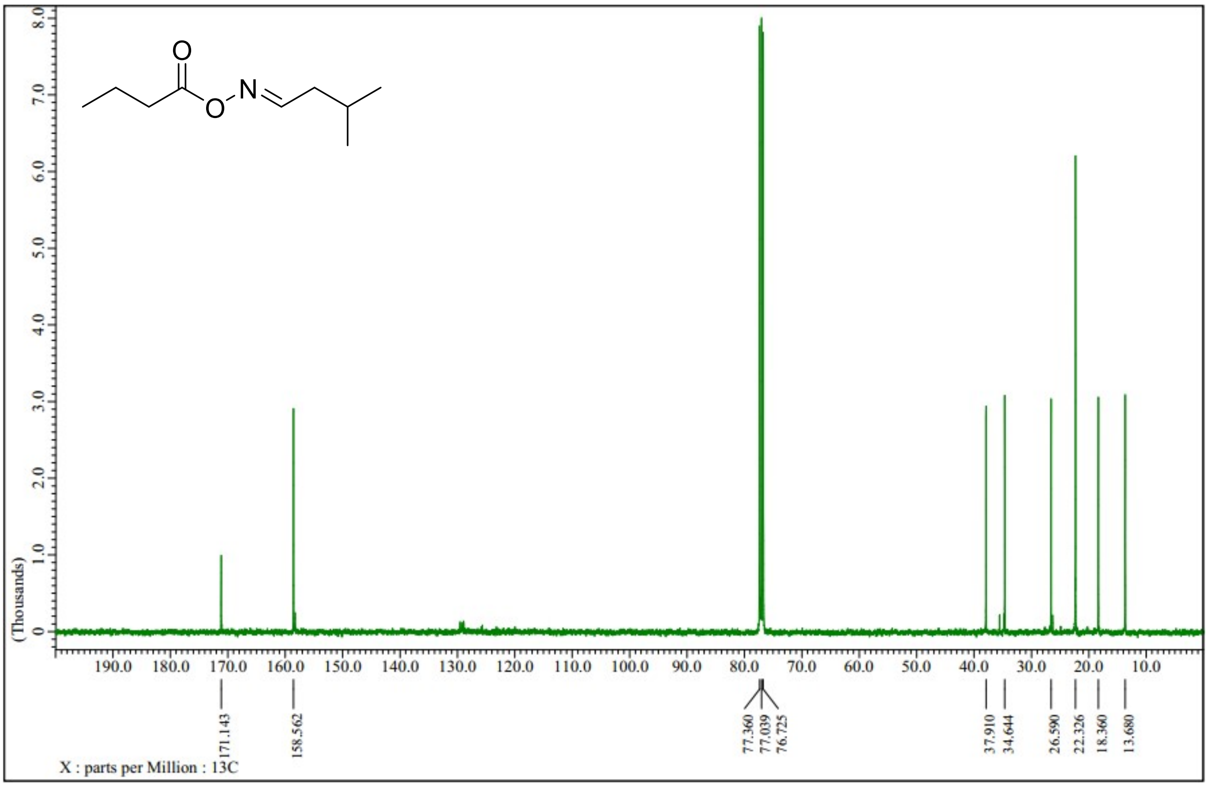

^{13}C NMR spectrum of (*E*)-3-methylbutanal O-butyryl oxime (**4l**)

(*E*)-Pivalaldehyde O-butyryl oxime (4m)

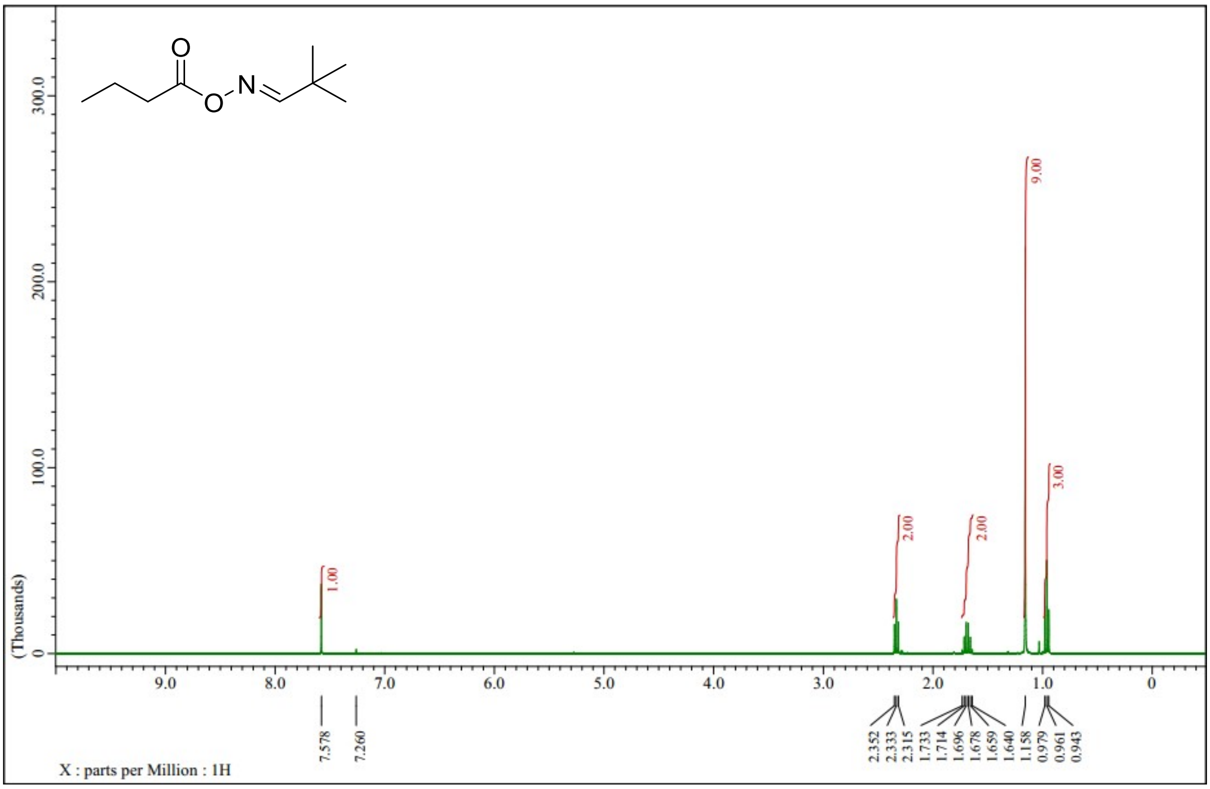

^{1}H NMR spectrum of (*E*)-pivalaldehyde O-butyryl oxime (**4m**)

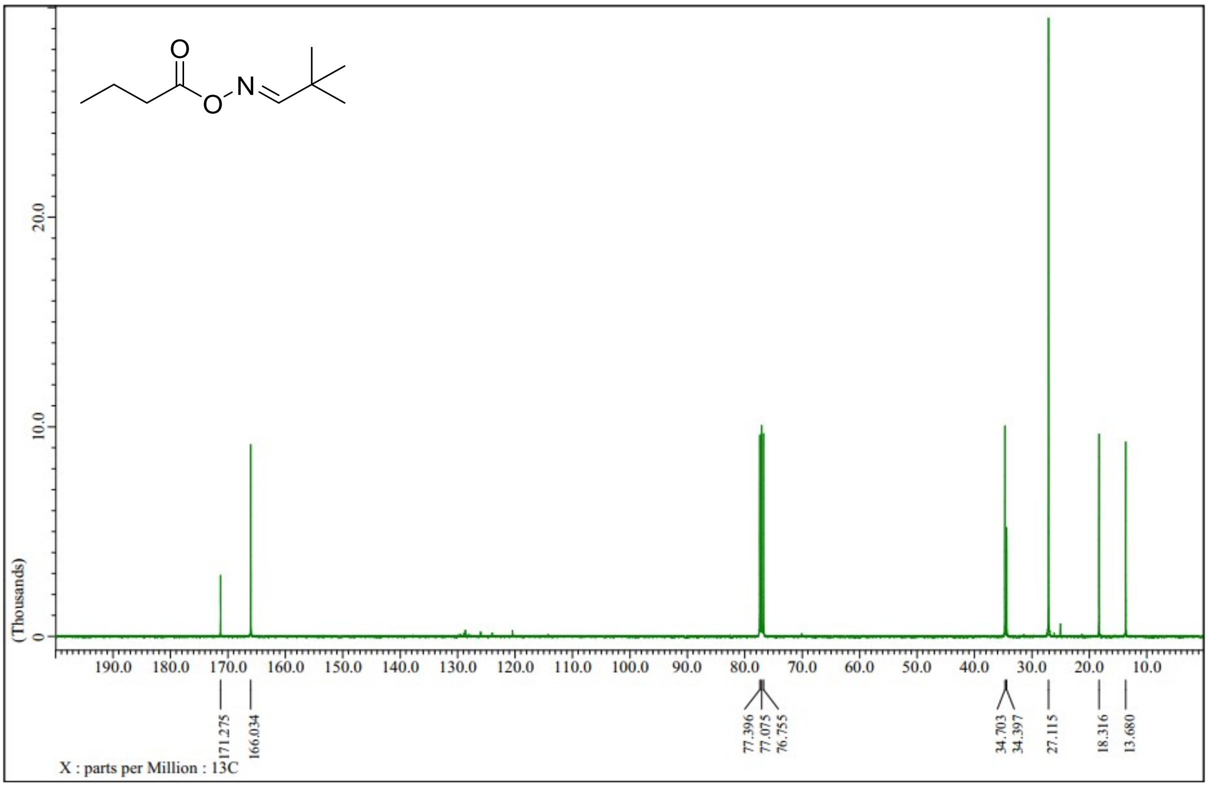

^{13}C NMR spectrum of (*E*)-pivalaldehyde O-butyryl oxime (**4m**)

(*E*)-Cyclohexanecarbaldehyde O-butyryl oxime (4n)

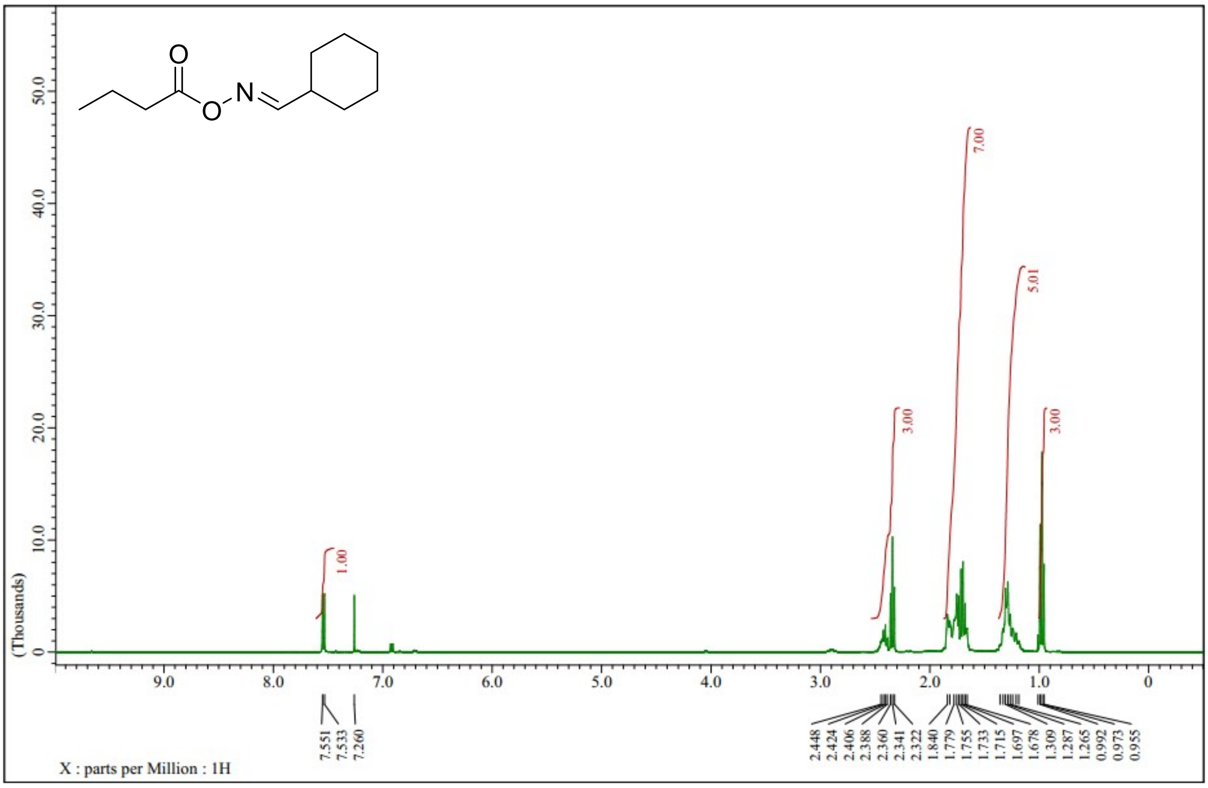

^{1}H NMR spectrum of (*E*)-cyclohexanecarbaldehyde O-butyryl oxime (**4n**)

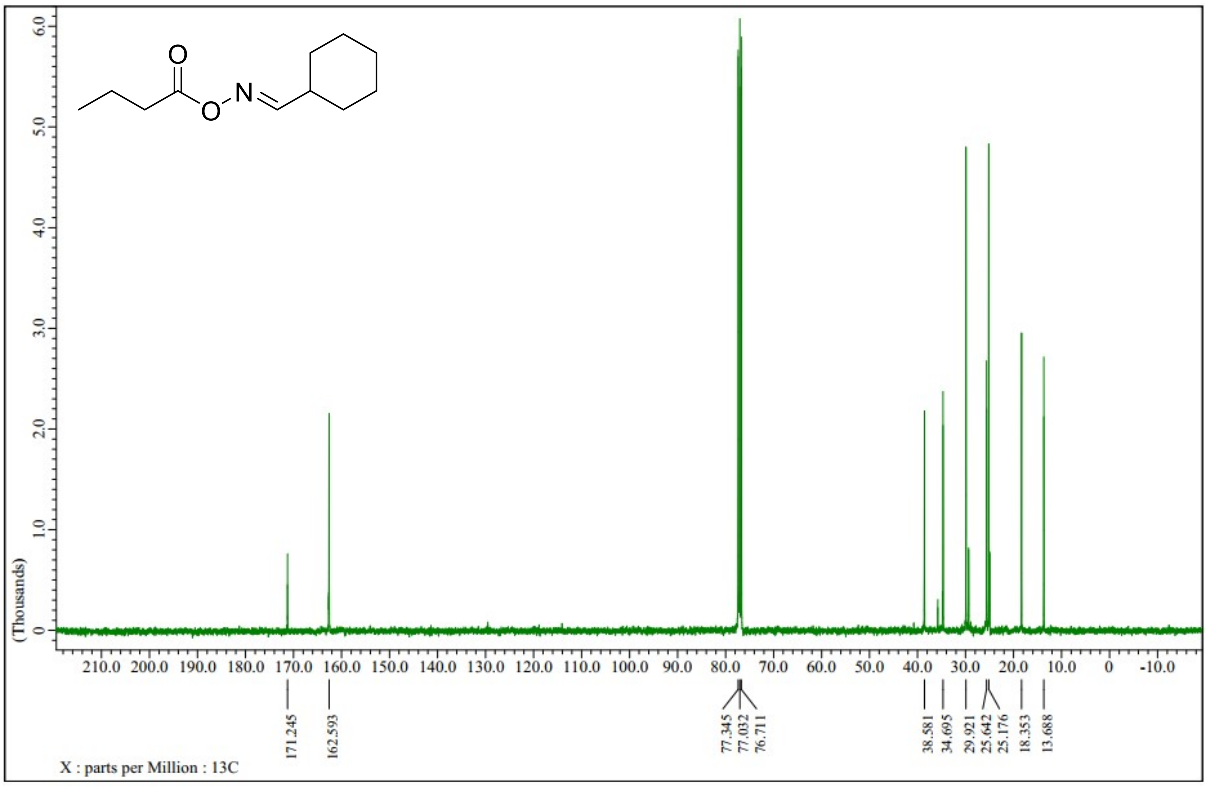

^{13}C NMR spectrum of (*E*)-cyclohexanecarbaldehyde O-butyryl oxime (**4n**)

(*E*)-Benzaldehyde O-benzoyl oxime (5a)

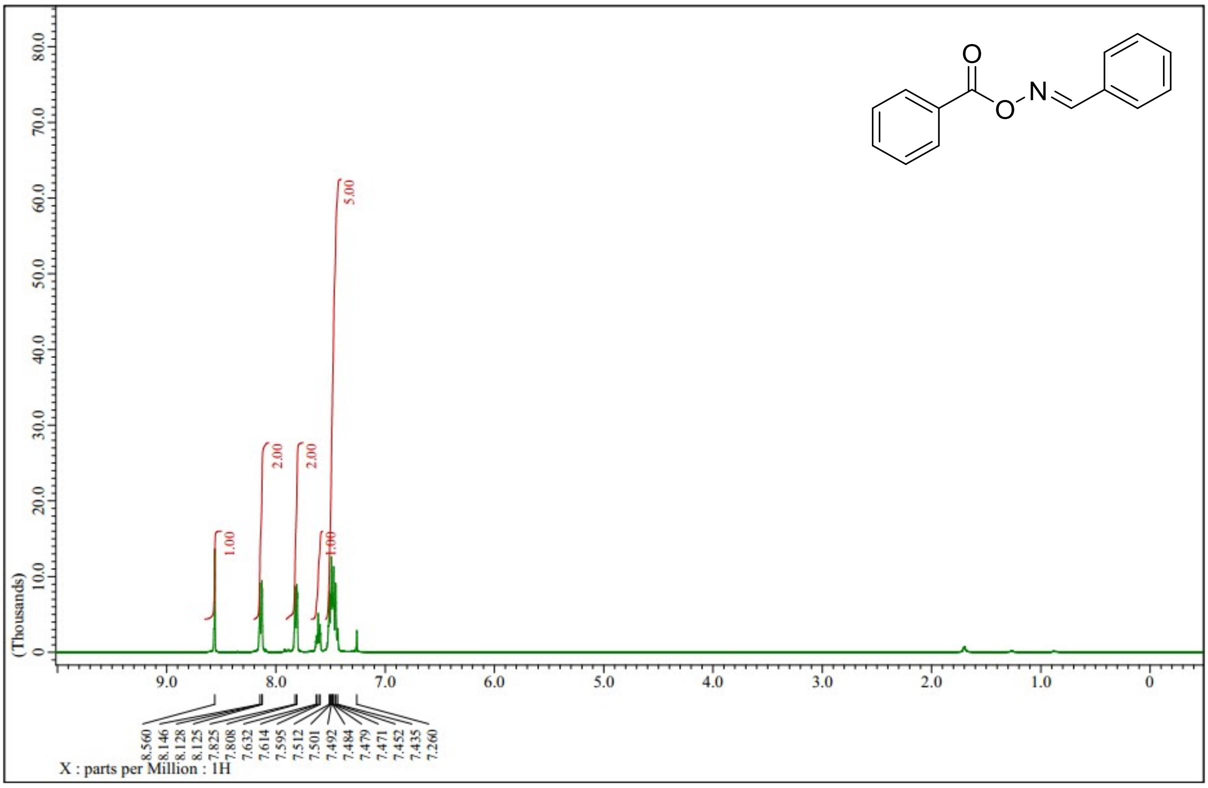

^{1}H NMR spectrum of (*E*)-benzaldehyde O-benzoyl oxime (**5a**)

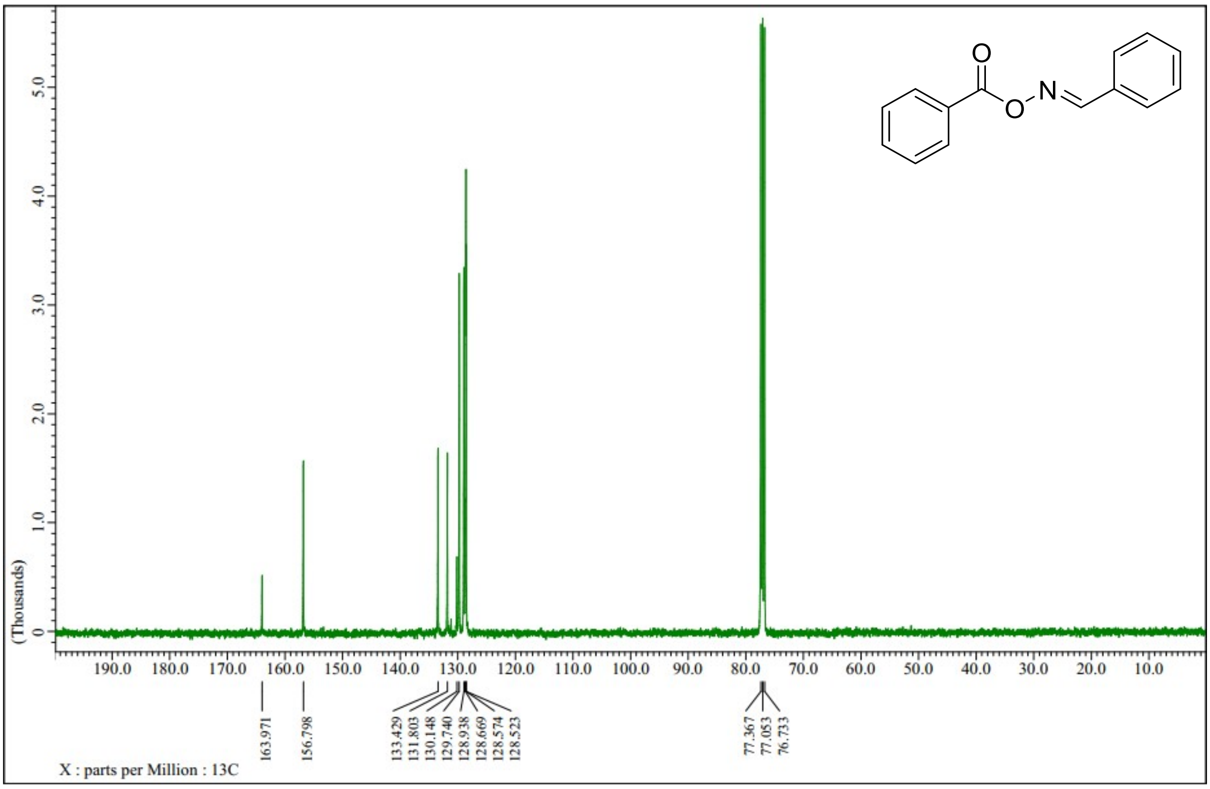

^{13}C NMR spectrum of (*E*)-benzaldehyde O-benzoyl oxime (**5a**)

(*E*)-Benzaldehyde O-(4-methoxybenzoyl) oxime (5b)

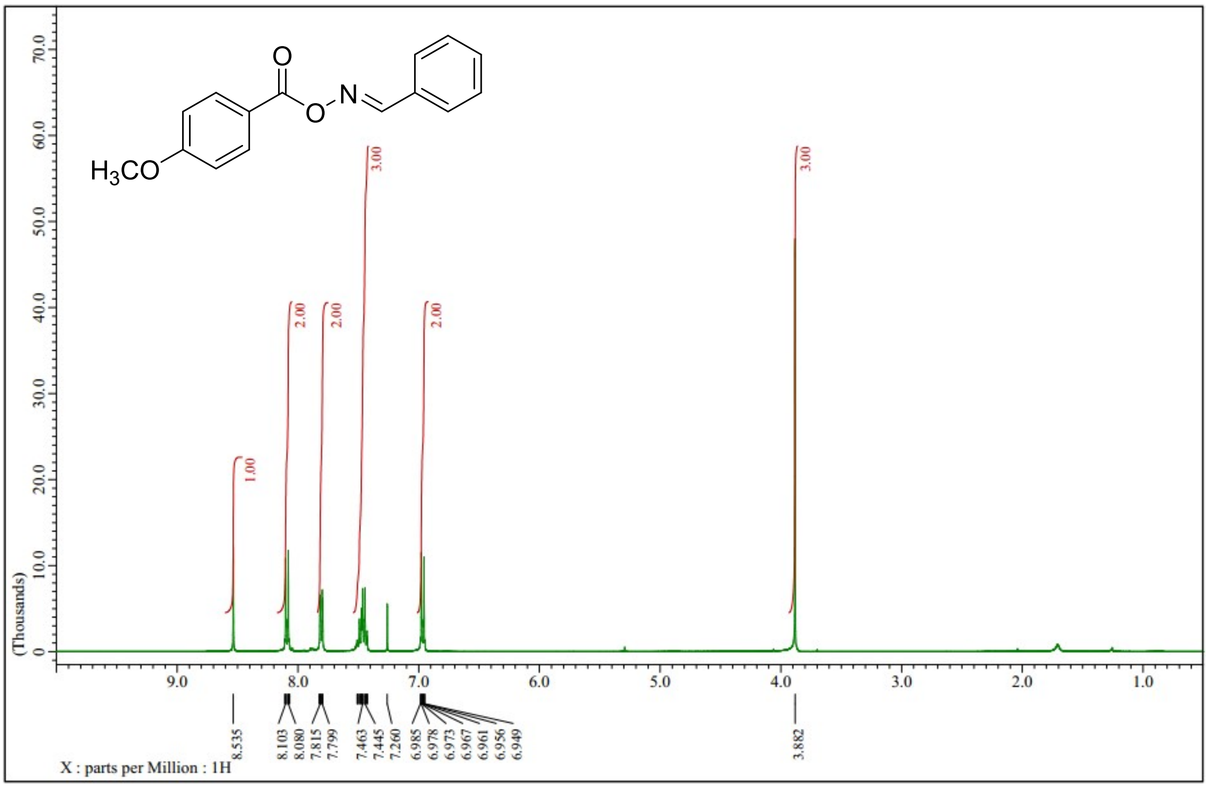

^{1}H NMR spectrum of (*E*)-benzaldehyde O-(4-methoxybenzoyl) oxime (**5b**)

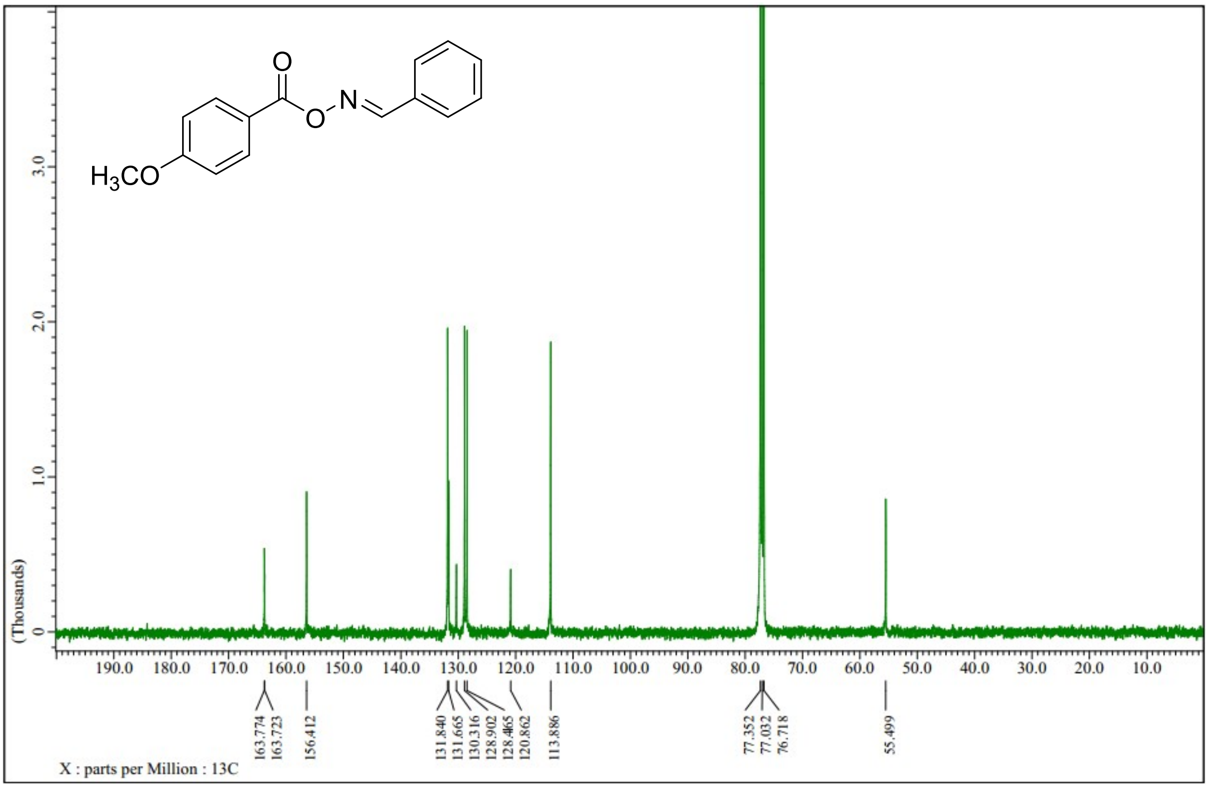

^{13}C NMR spectrum of (*E*)-benzaldehyde O-(4-methoxybenzoyl) oxime (**5b**)

(*E*)-Benzaldehyde O-(4-methylbenzoyl) oxime (5c)

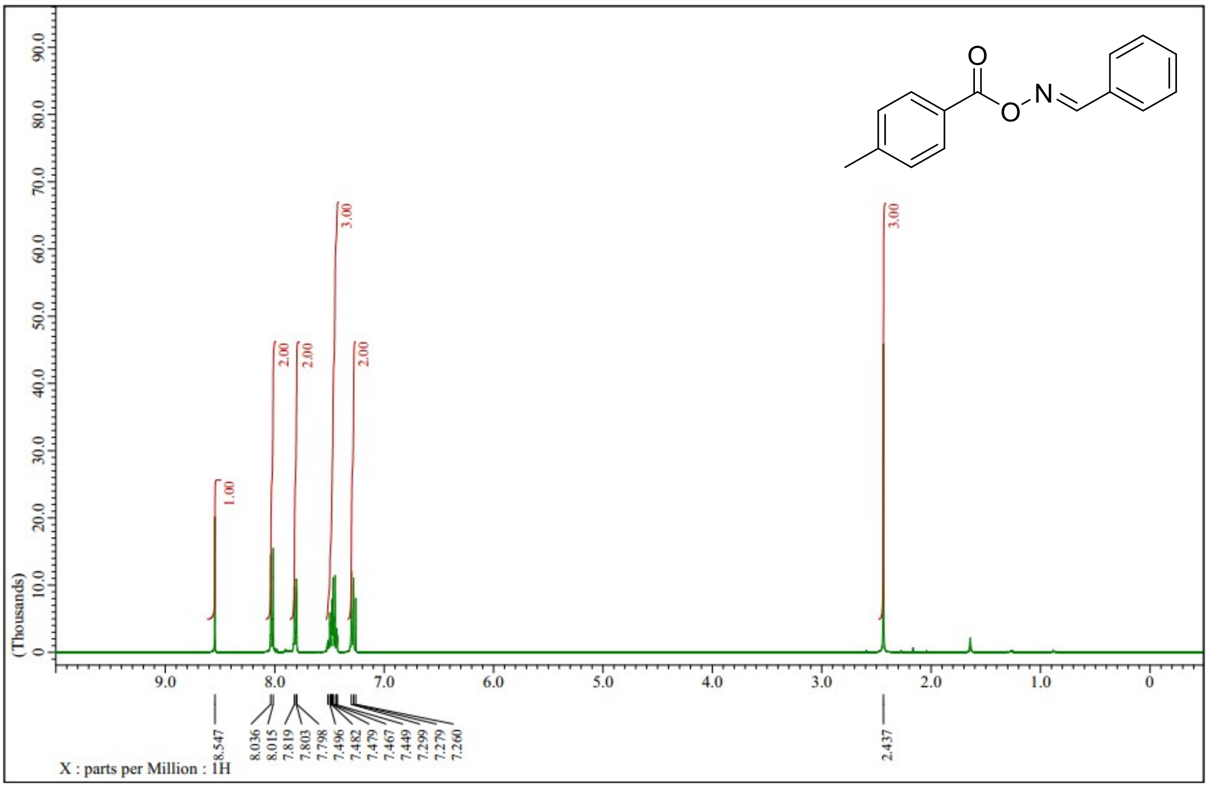

^{1}H NMR spectrum of (*E*)-benzaldehyde O-(4-methylbenzoyl) oxime (**5c**)

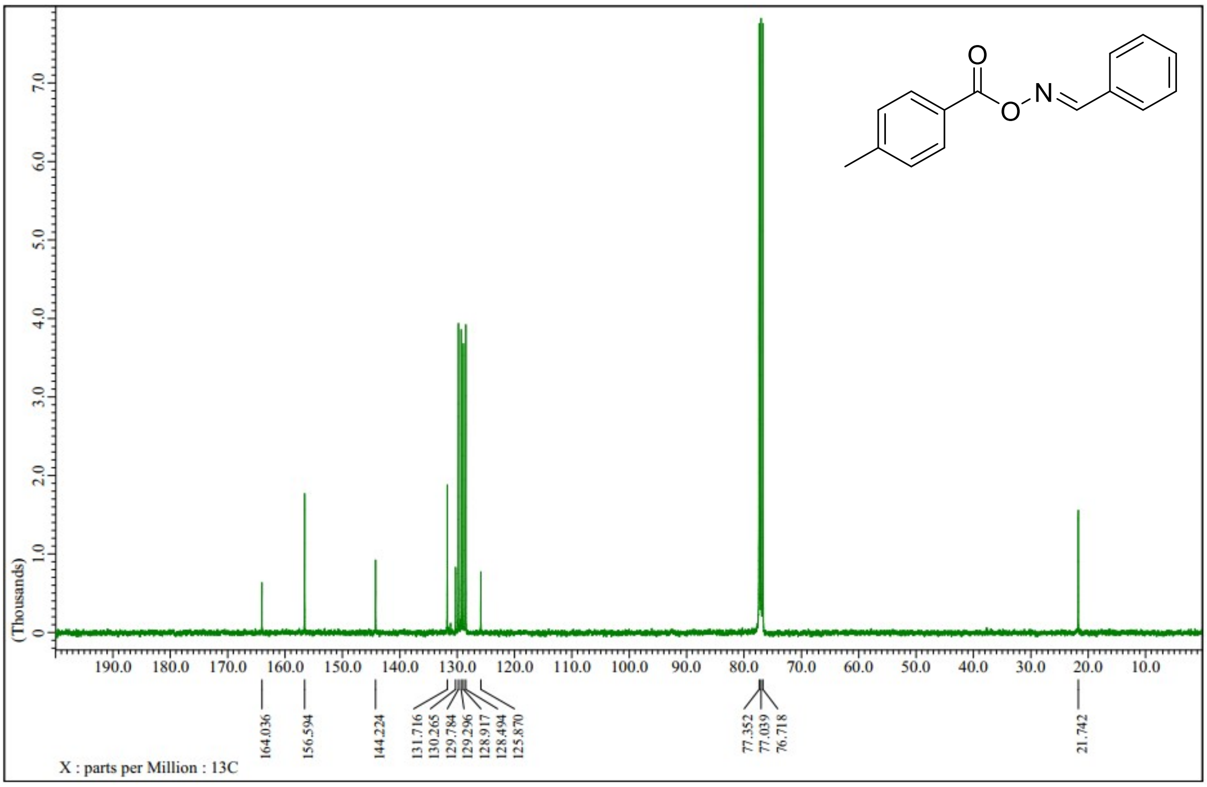

^{13}C NMR spectrum of (*E*)-benzaldehyde O-(4-methylbenzoyl) oxime (**5c**)

(*E*)-Benzaldehyde O-(2,4,6-trimethylbenzoyl) oxime (5d)

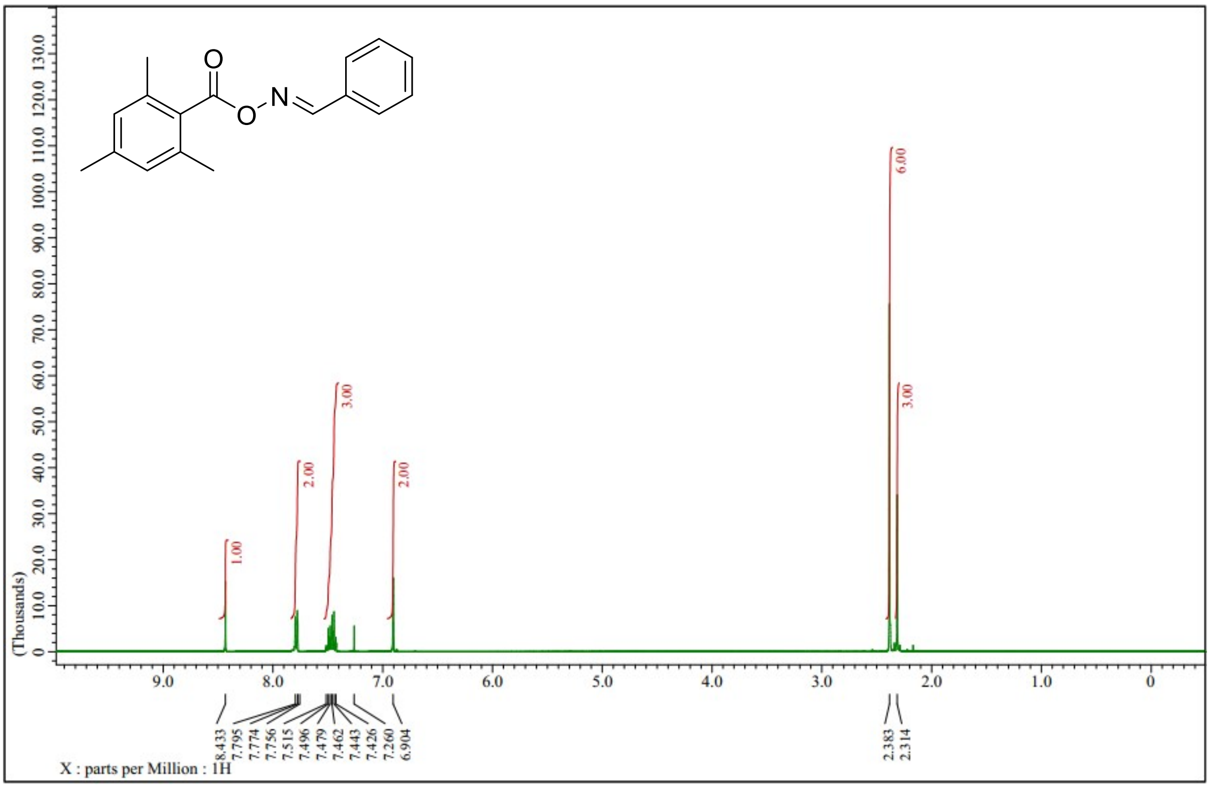

^{1}H NMR spectrum of (*E*)-benzaldehyde O-(2,4,6-trimethylbenzoyl) oxime (**5d**)

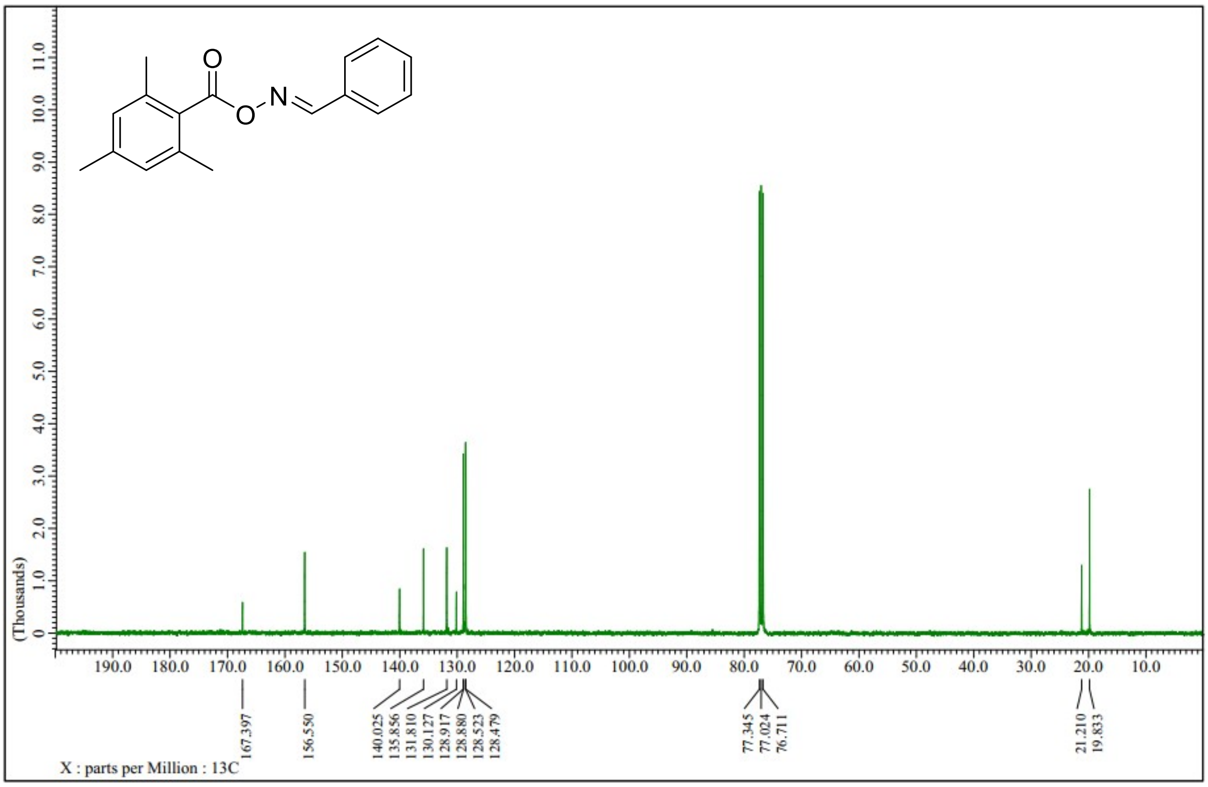

^{13}C NMR spectrum of (*E*)-benzaldehyde O-(2,4,6-trimethylbenzoyl) oxime (**5d**)

(*E*)-Benzaldehyde O-(4-chlorobenzoyl) oxime (5e)

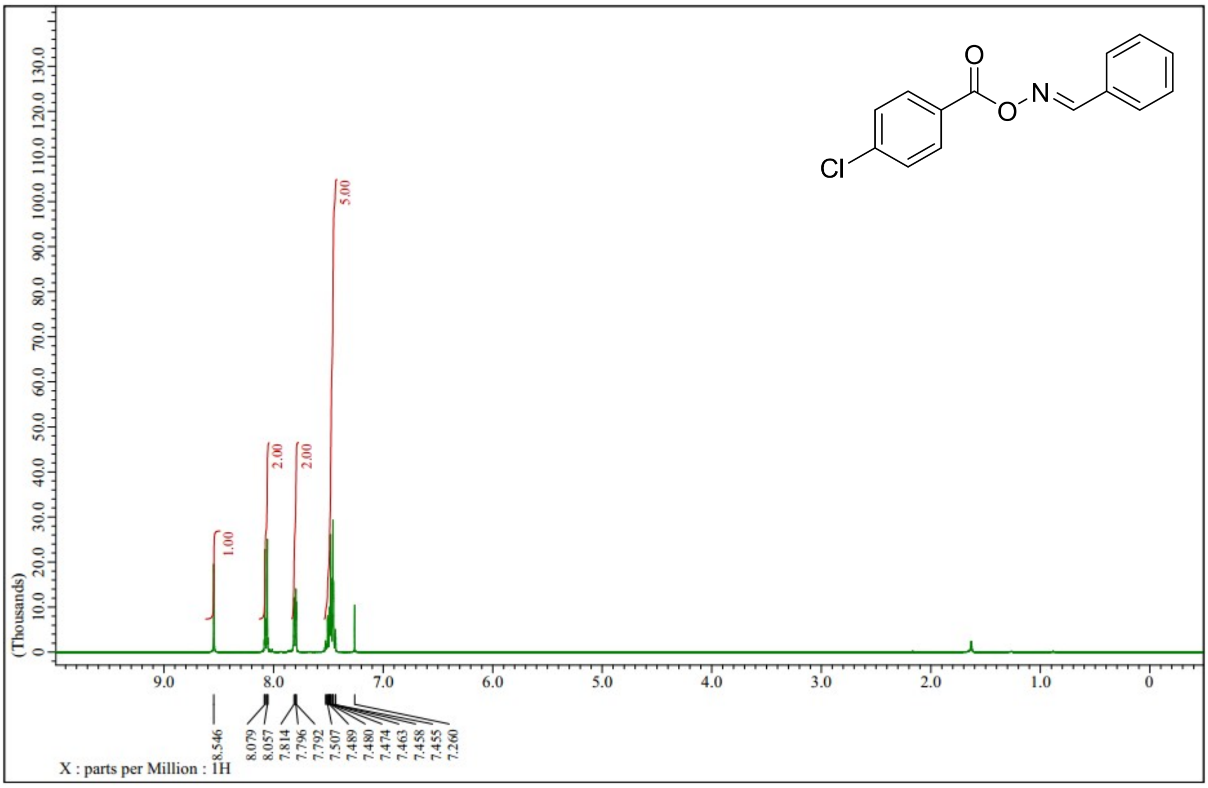

^{1}H NMR spectrum of (*E*)-benzaldehyde O-(4-chlorobenzoyl) oxime (**5e**)

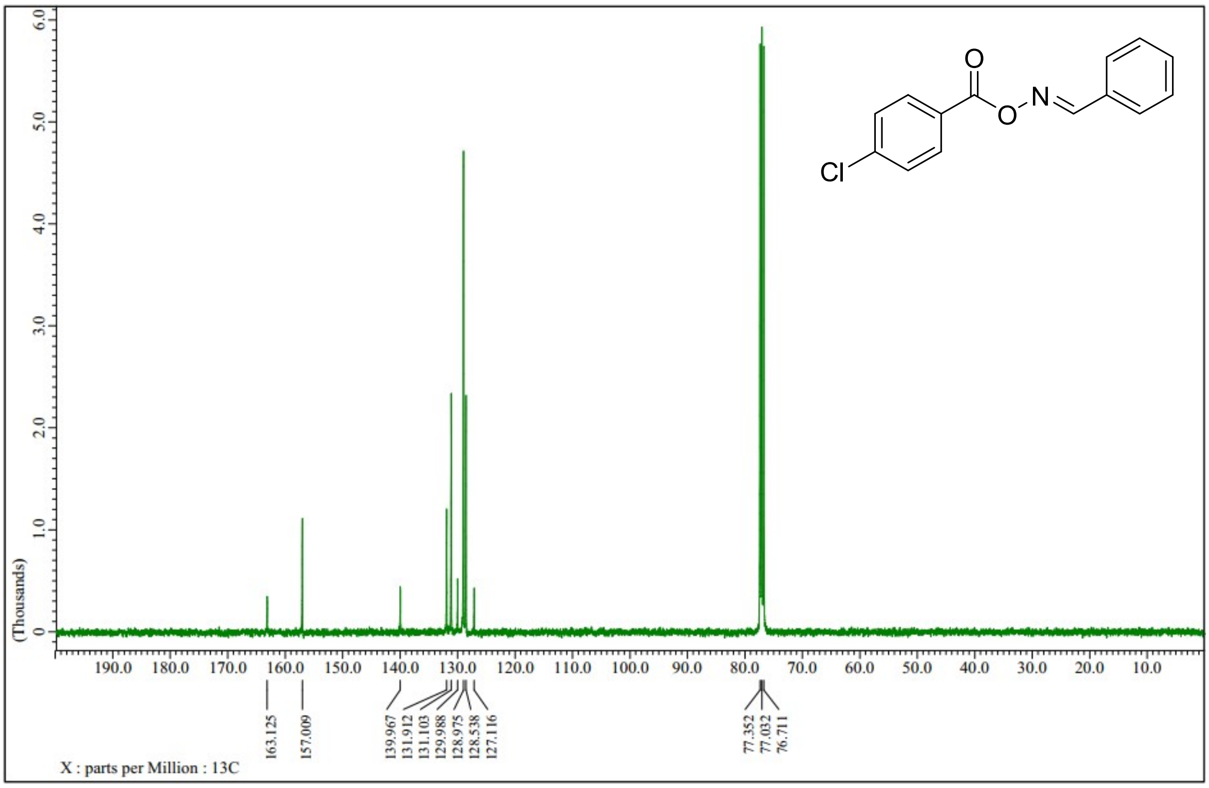

^{13}C NMR spectrum of (*E*)-benzaldehyde O-(4-chlorobenzoyl) oxime (**5e**)

(*E*)-Benzaldehyde O-(4-(trifluoromethyl)benzoyl) oxime (5f)

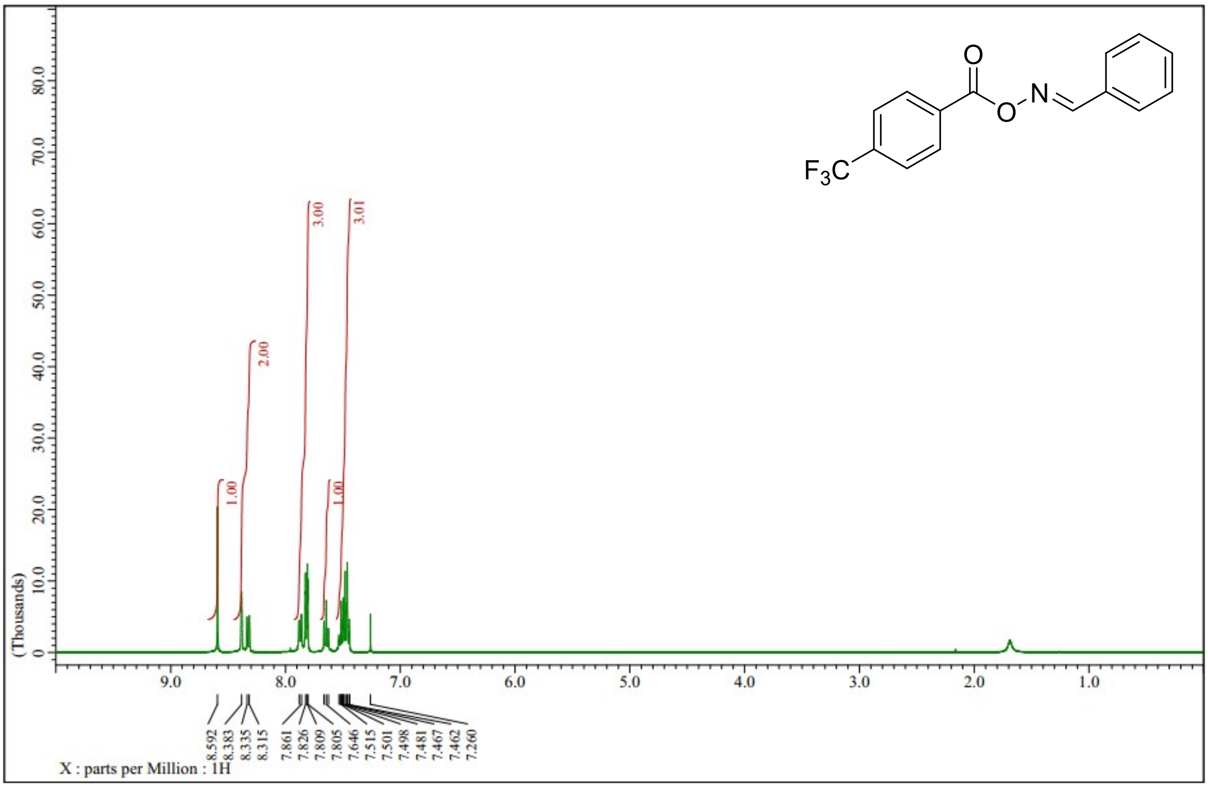

^{1}H NMR spectrum of (*E*)-benzaldehyde O-(4-(trifluoromethyl)benzoyl) oxime (**5f**)

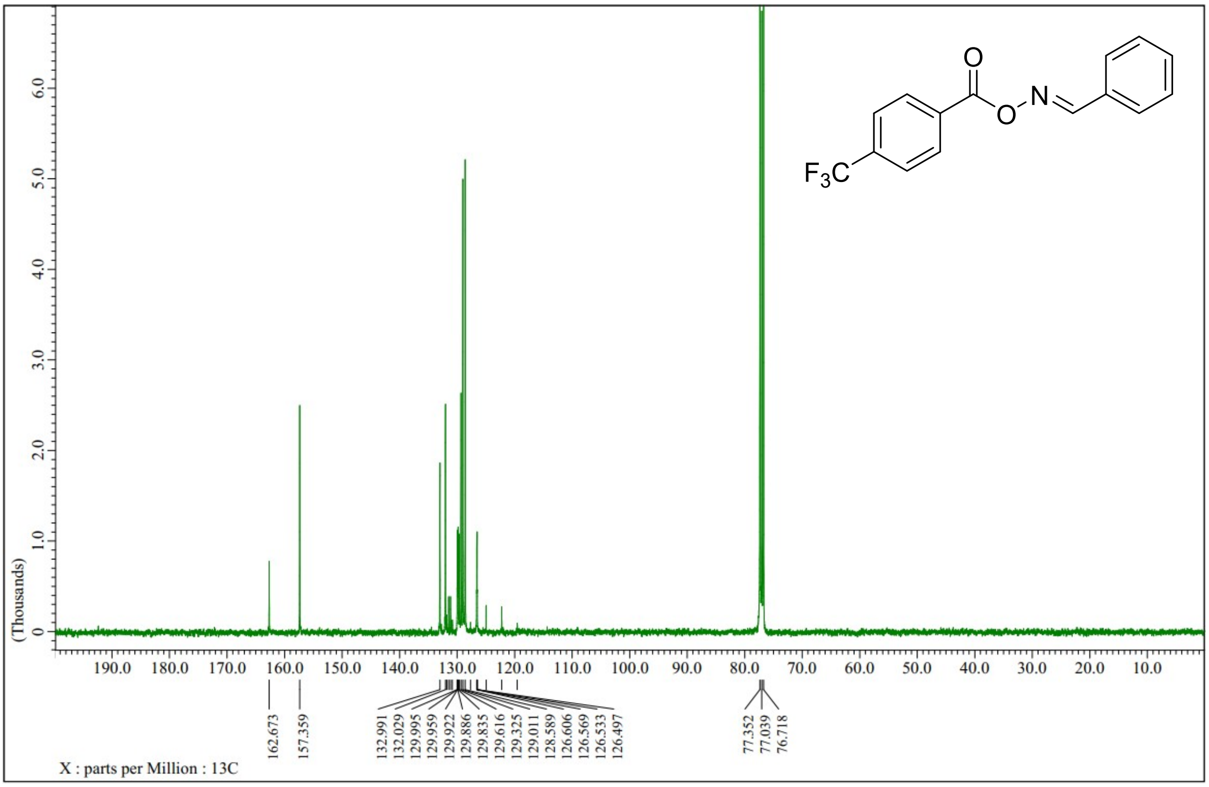

^{13}C NMR spectrum of (*E*)-benzaldehyde O-(4-(trifluoromethyl)benzoyl) oxime (**5f**)

(*E*)-Benzaldehyde O-(4-nitrobenzoyl) oxime (5g)

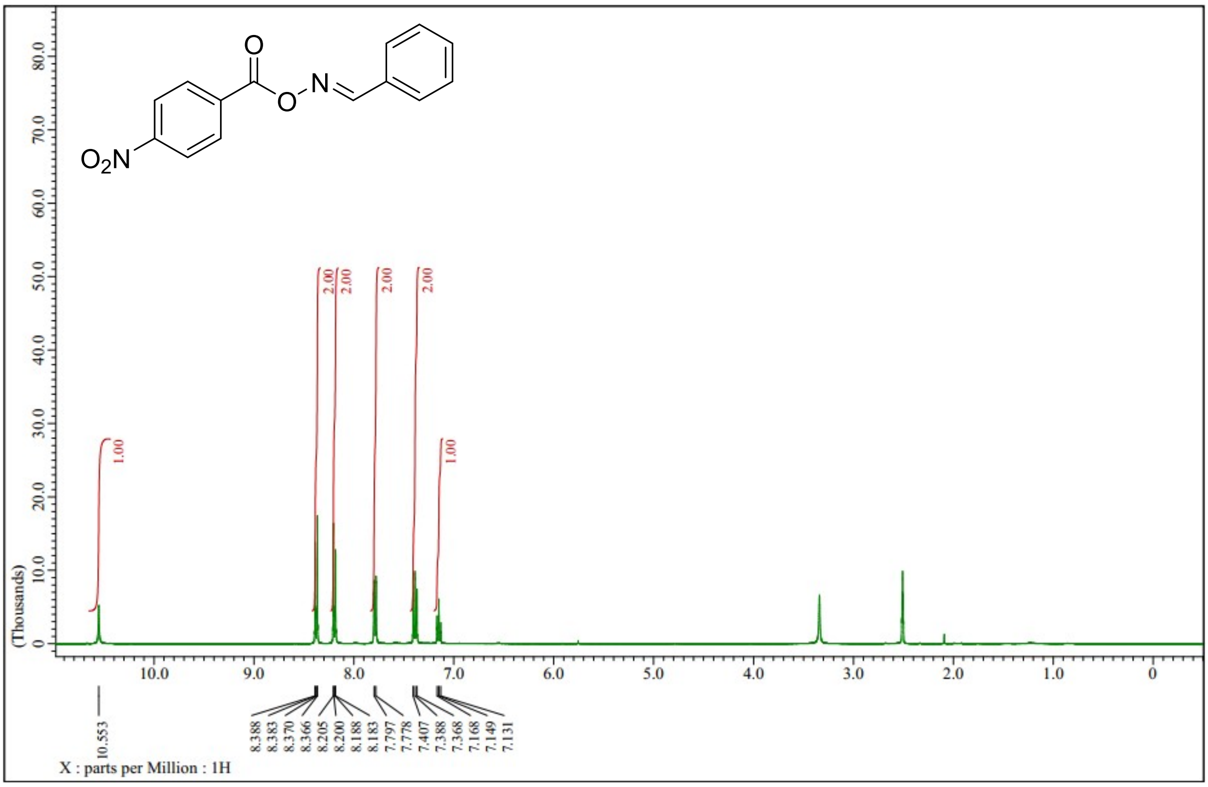

^{1}H NMR spectrum of (*E*)-benzaldehyde O-(4-nitrobenzoyl) oxime (**5g**)

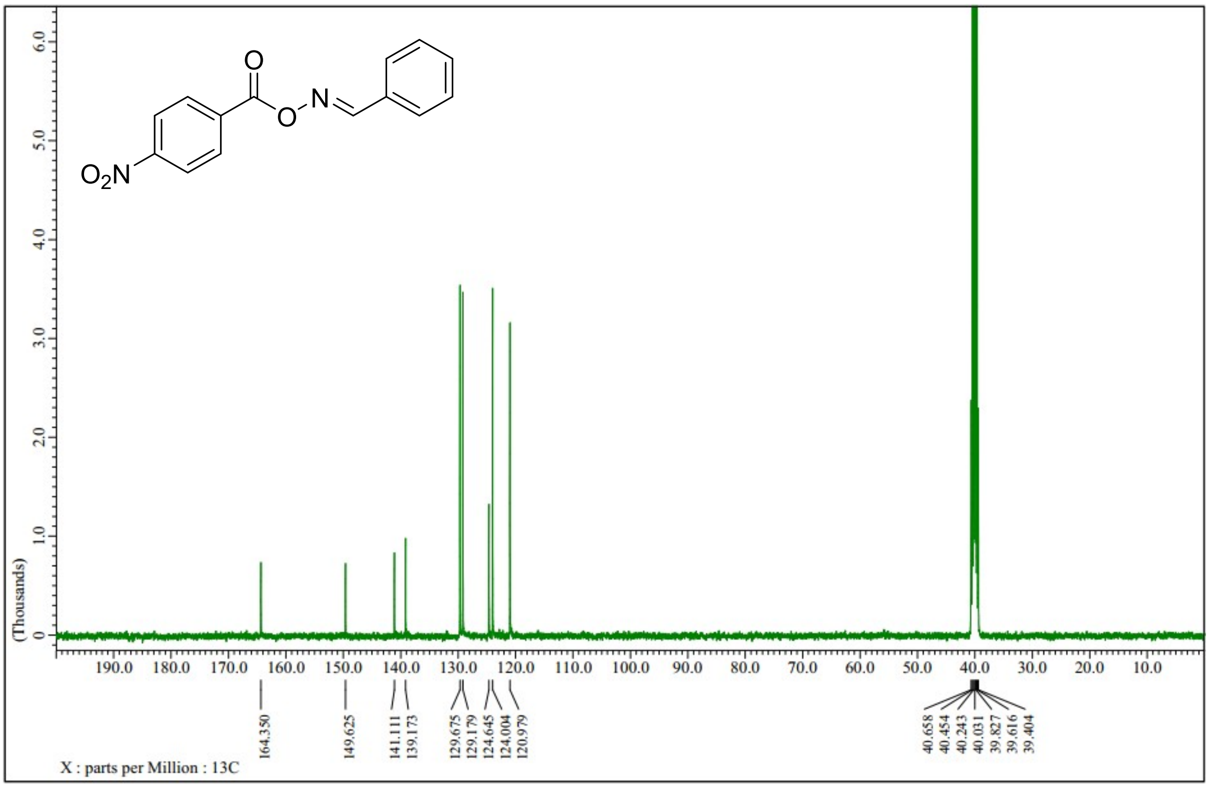

^{13}C NMR spectrum of (*E*)-benzaldehyde O-(4-nitrobenzoyl) oxime (**5g**)

(*E*)-Benzaldehyde O-(3-bromobenzoyl) oxime (5h)

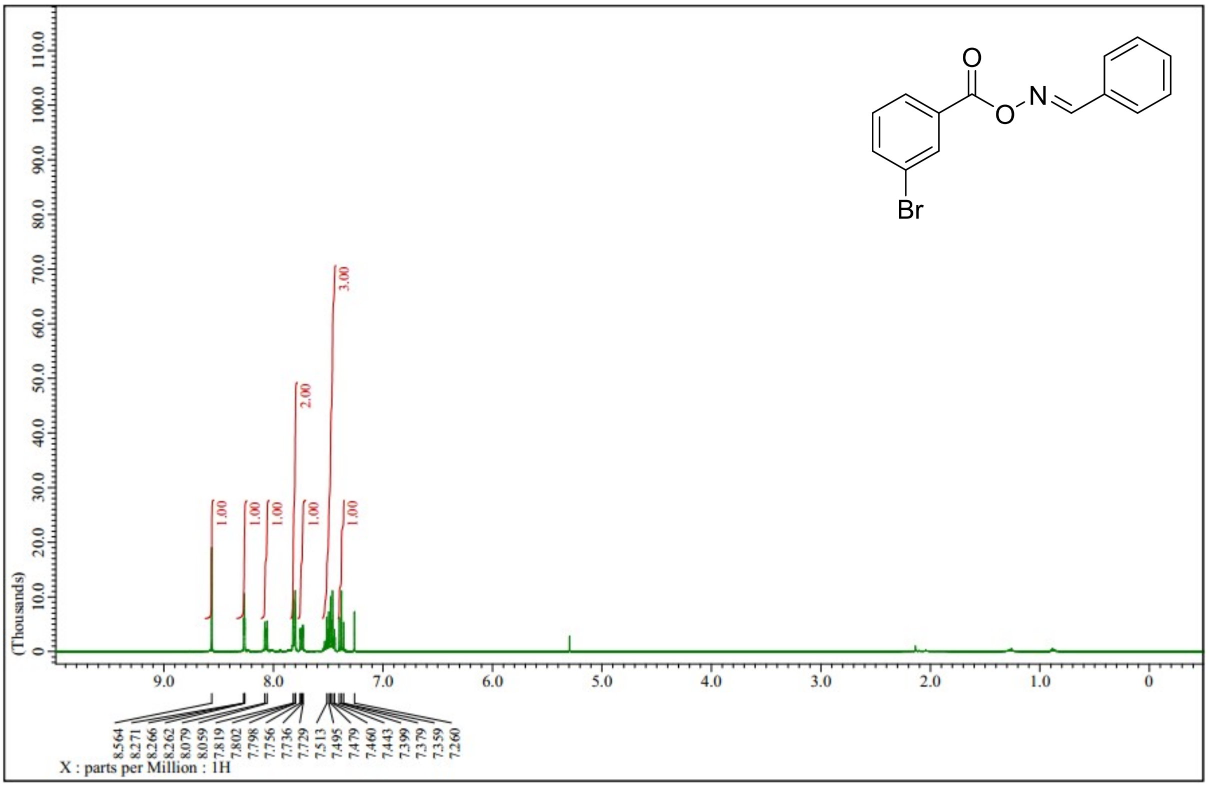

^{1}H NMR spectrum of (*E*)-benzaldehyde O-(3-bromobenzoyl) oxime (**5h**)

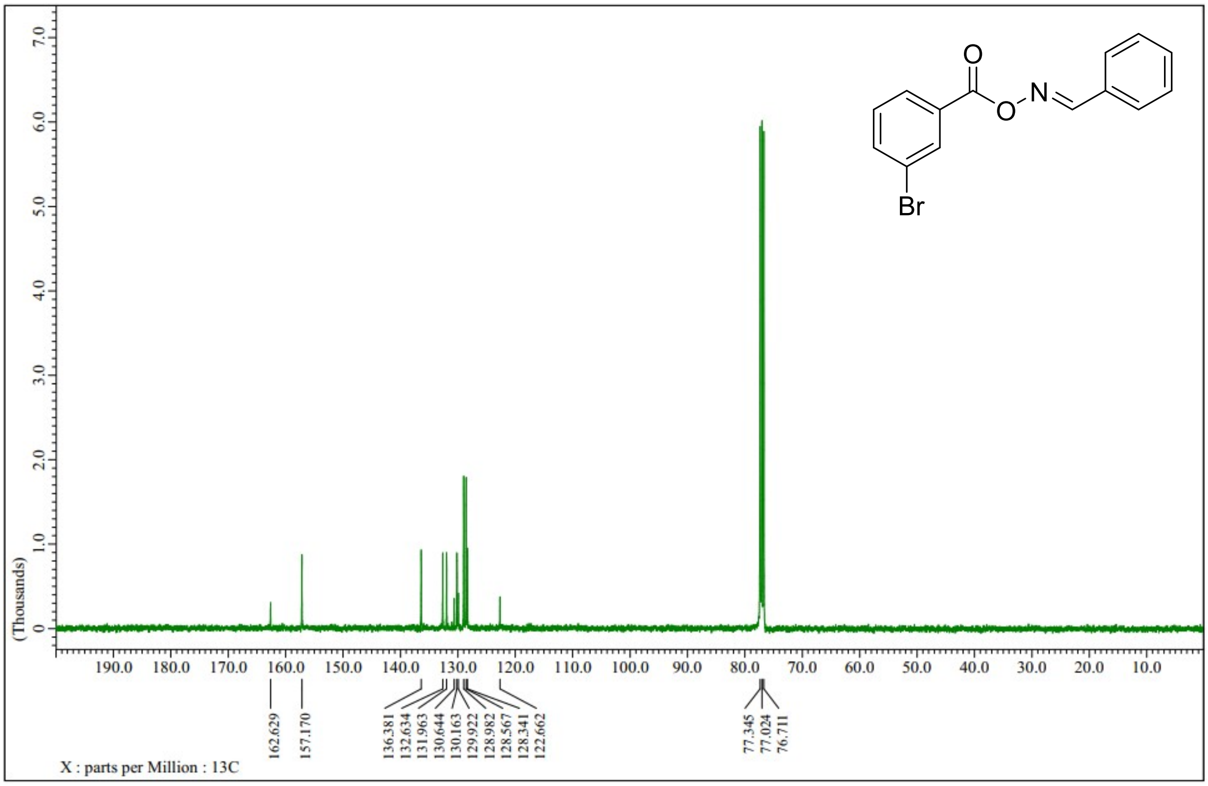

^{13}C NMR spectrum of (*E*)-benzaldehyde O-(3-bromobenzoyl) oxime (**5h**)

(*E*)-Benzaldehyde O-(1-naphthoyl) oxime (5i)

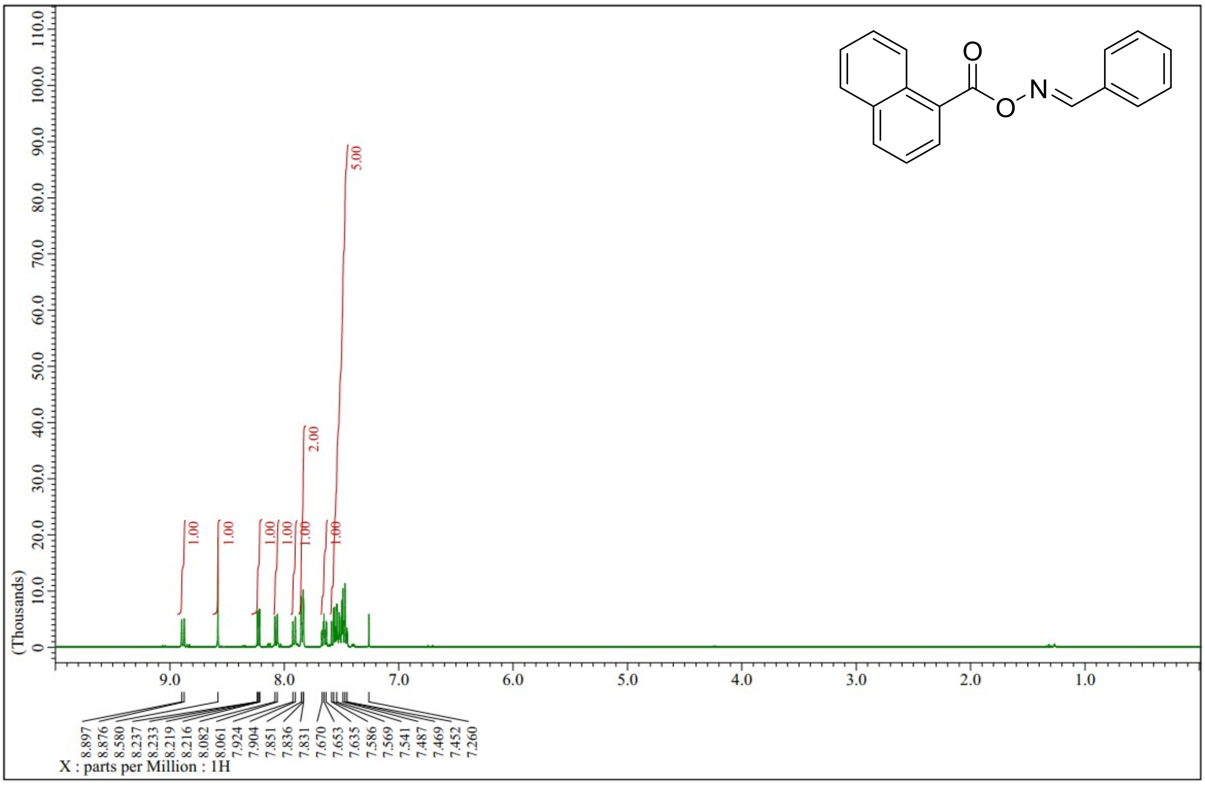

^{1}H NMR spectrum of (*E*)-benzaldehyde O-(1-naphthoyl) oxime (**5i**)

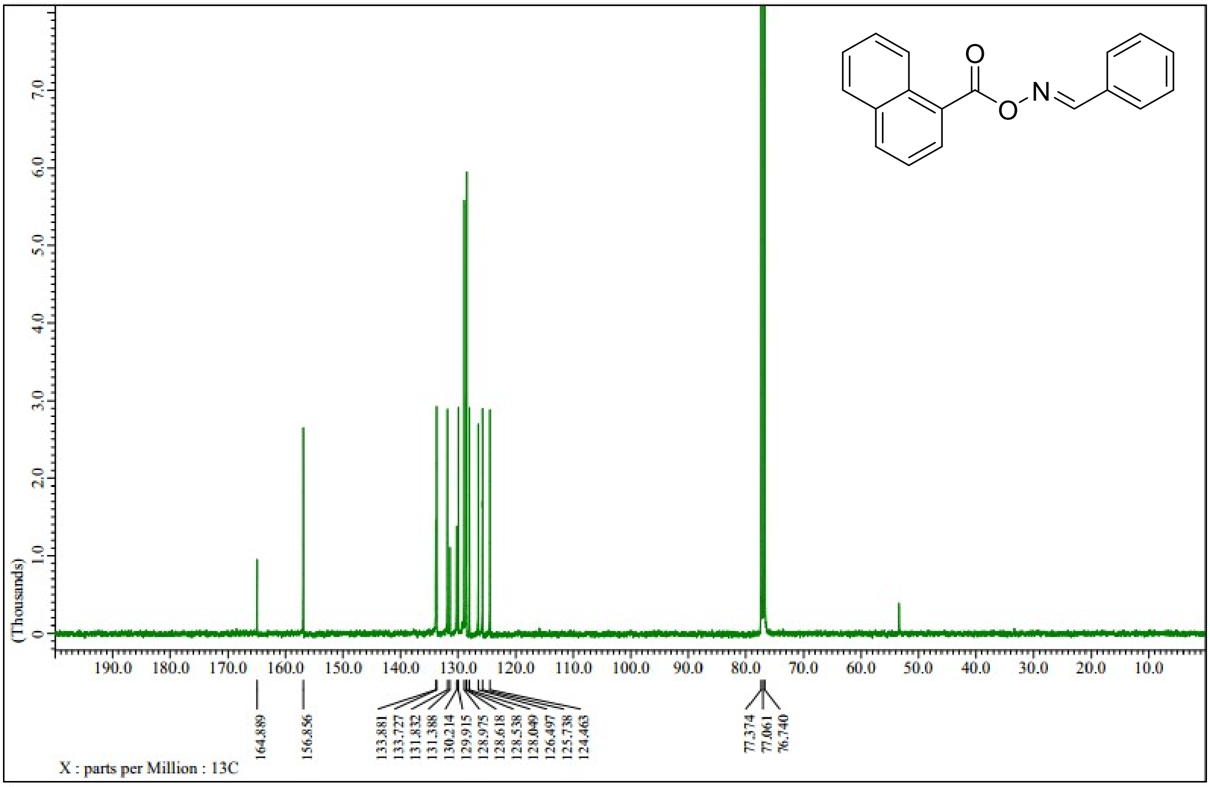

^{13}C NMR spectrum of (*E*)-benzaldehyde O-(1-naphthoyl) oxime (**5i**)

(*E*)-Benzaldehyde O-([1,1'-biphenyl]-4-carbonyl) oxime (5j)

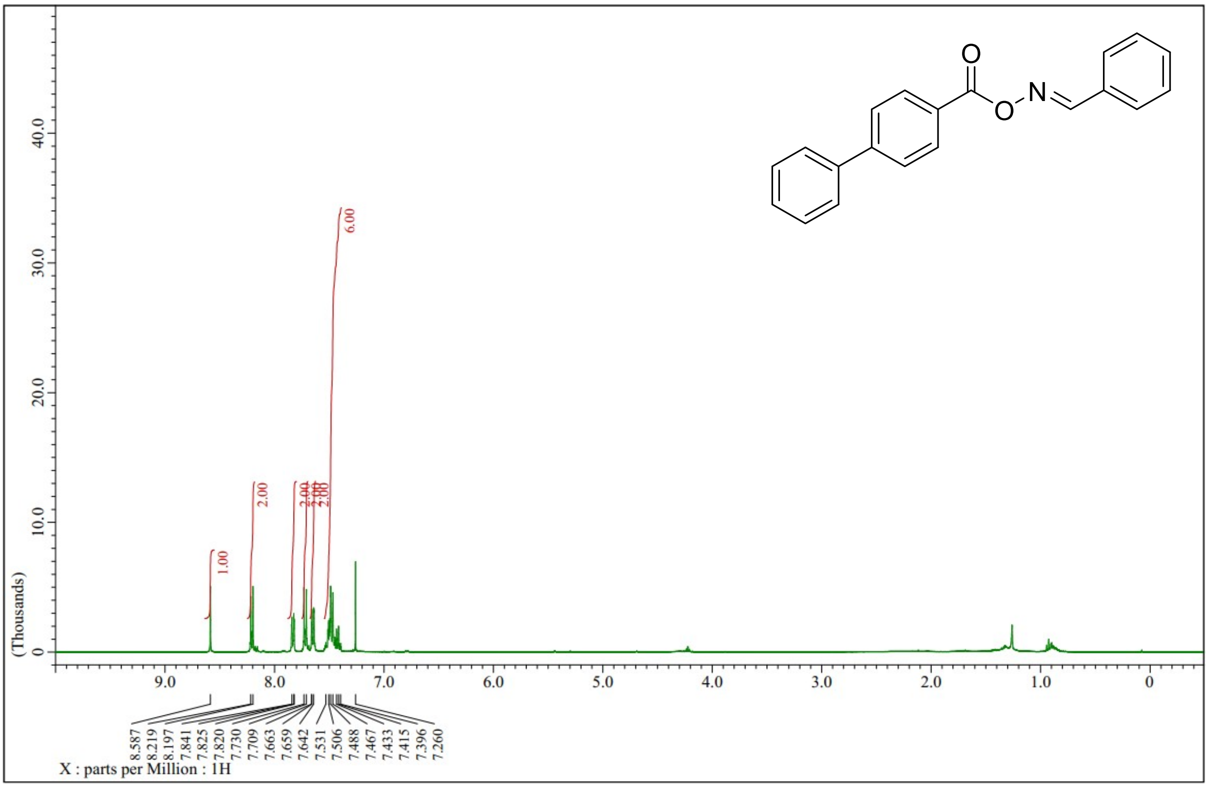

^{1}H NMR spectrum of (*E*)-benzaldehyde O-([1,1'-biphenyl]-4-carbonyl) oxime (**5j**)

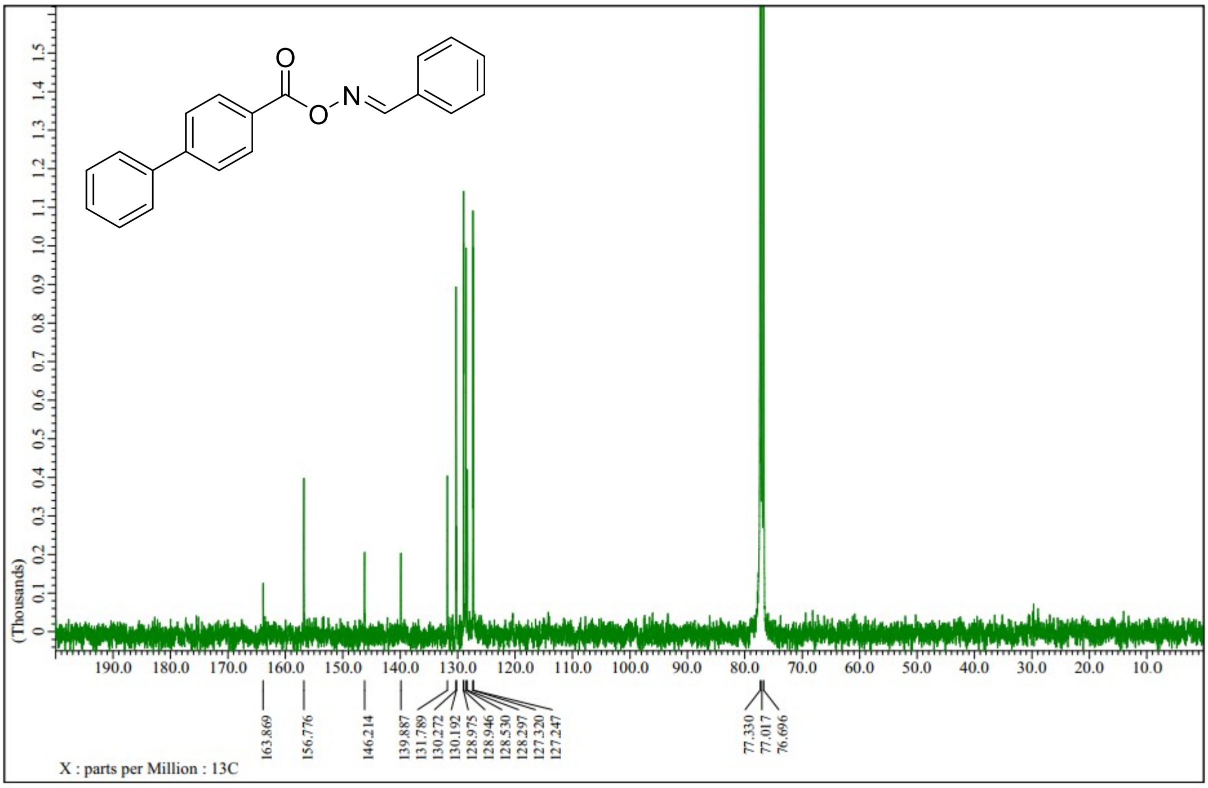

^{13}C NMR spectrum of (*E*)-benzaldehyde O-([1,1'-biphenyl]-4-carbonyl) oxime (**5j**)

(*E*)-Benzaldehyde O-(3,3-dimethylbutanoyl) oxime (5k)

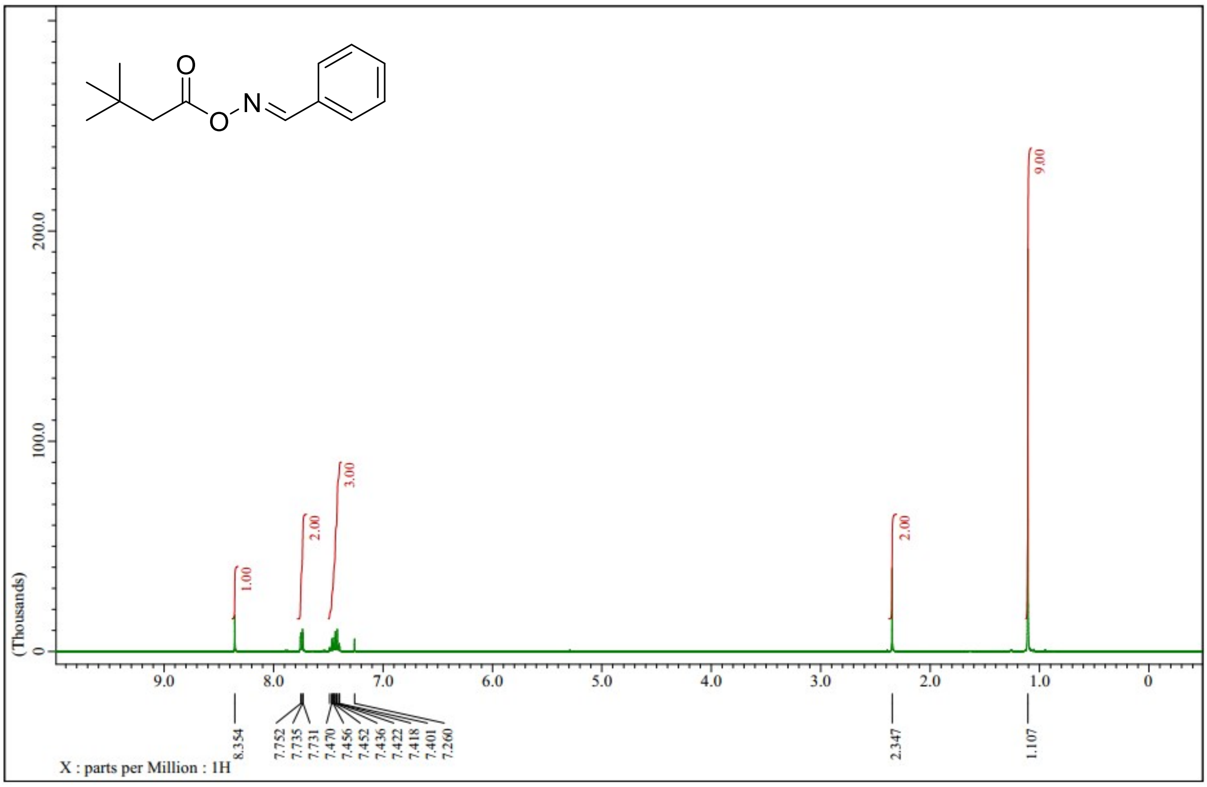

^{1}H NMR spectrum of (*E*)-benzaldehyde O-(3,3-dimethylbutanoyl) oxime (**5k**)

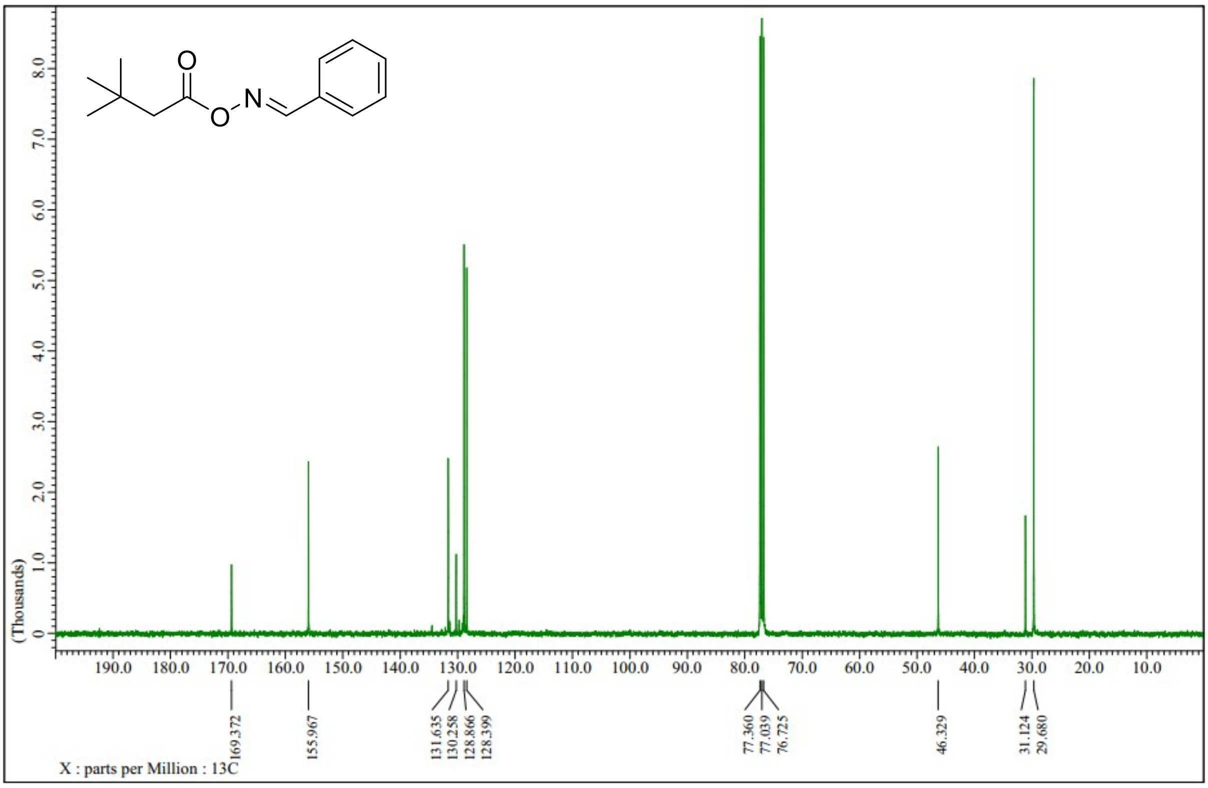

^{13}C NMR spectrum of (*E*)-benzaldehyde O-(3,3-dimethylbutanoyl) oxime (**5k**)

(*E*)-Benzaldehyde O-(2-ethylhexanoyl) oxime (5l)

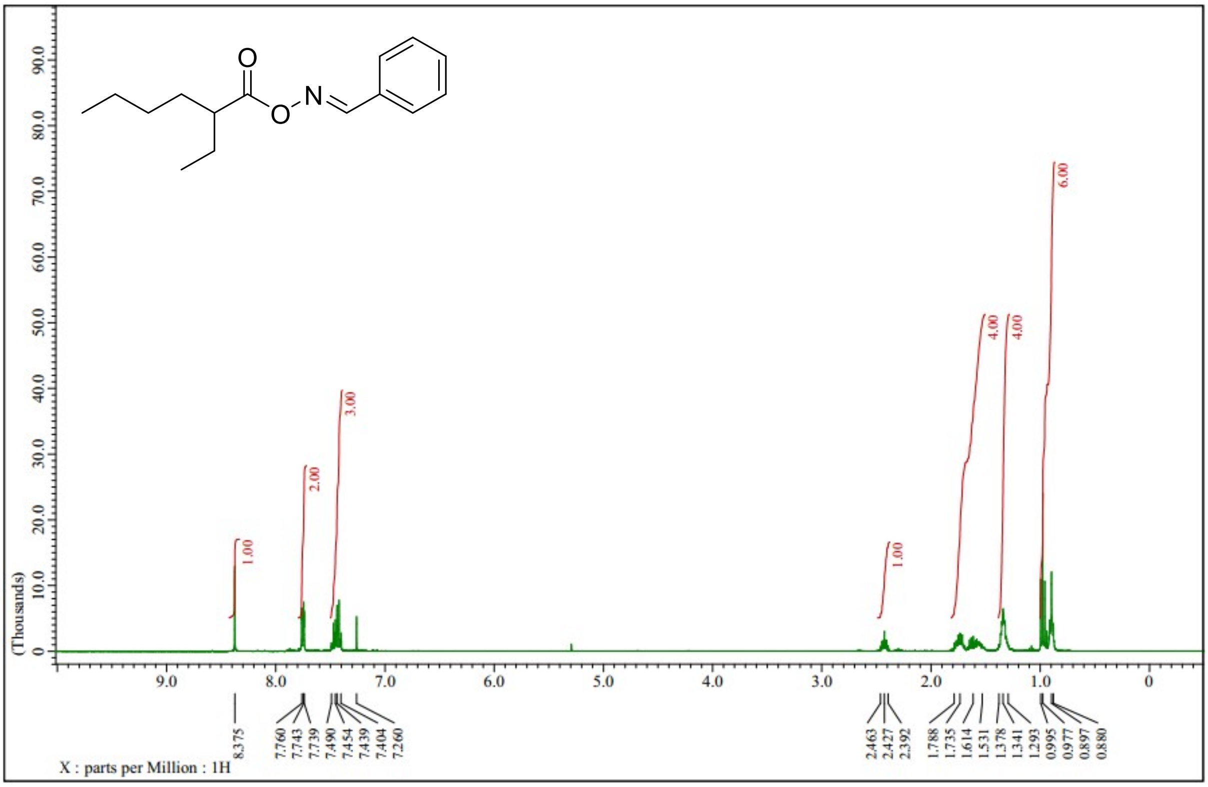

^{1}H NMR spectrum of (*E*)-benzaldehyde O-(2-ethylhexanoyl) oxime (**5l**)

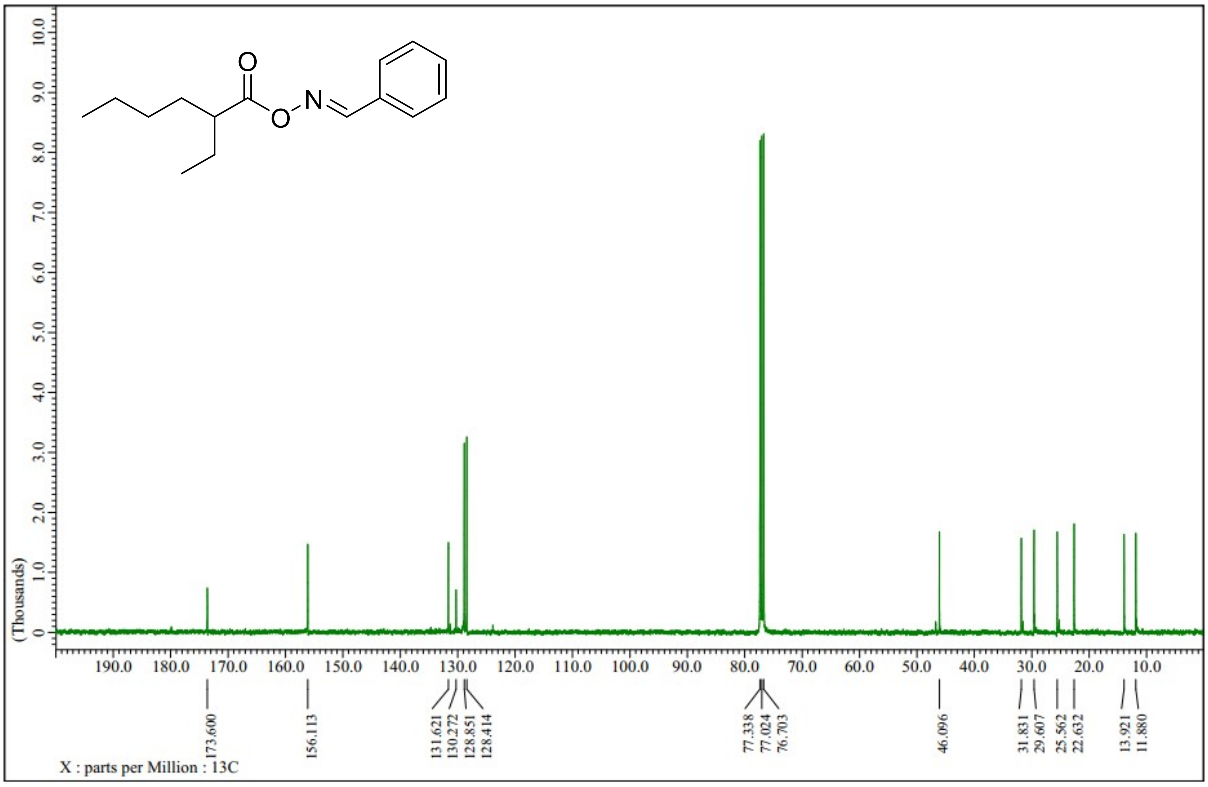

^{13}C NMR spectrum of (*E*)-benzaldehyde O-(2-ethylhexanoyl) oxime (**5l**)

(*E*)-Benzaldehyde O-undecanoyl oxime (5m)

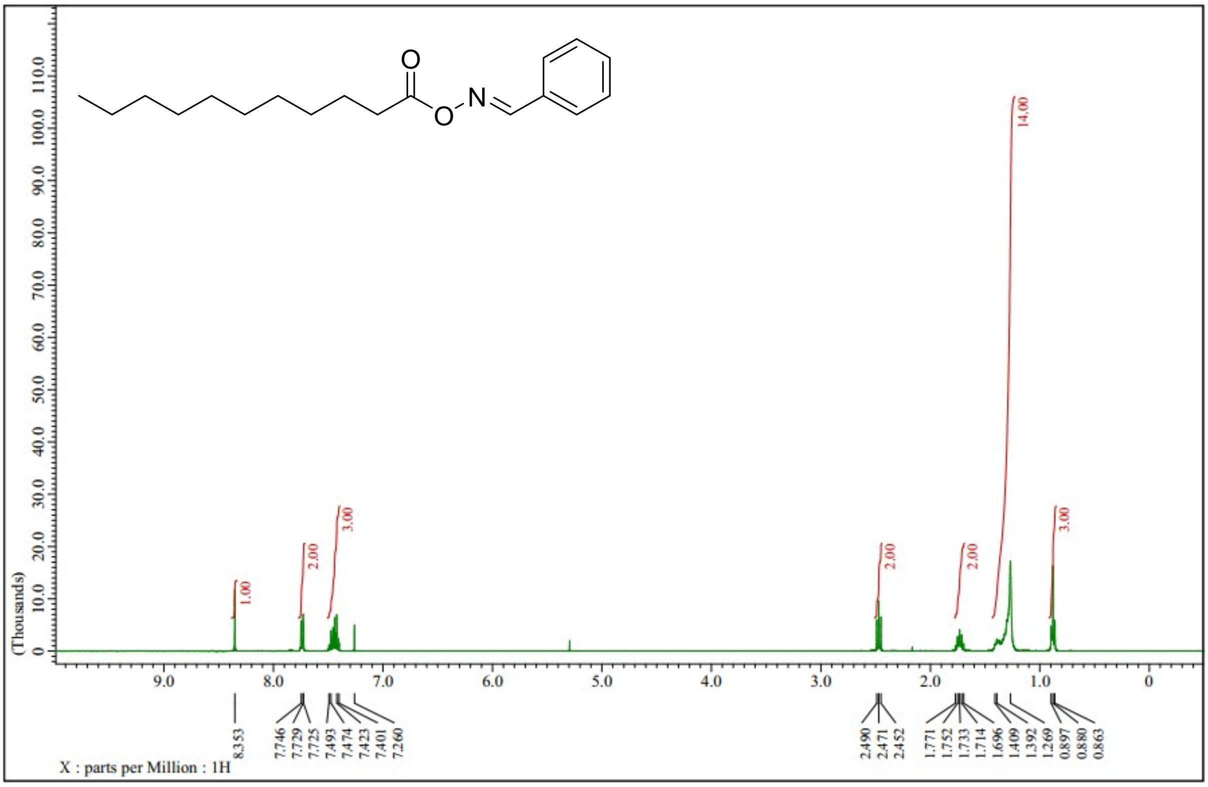

^{1}H NMR spectrum of (*E*)-benzaldehyde O-undecanoyl oxime (**5m**)

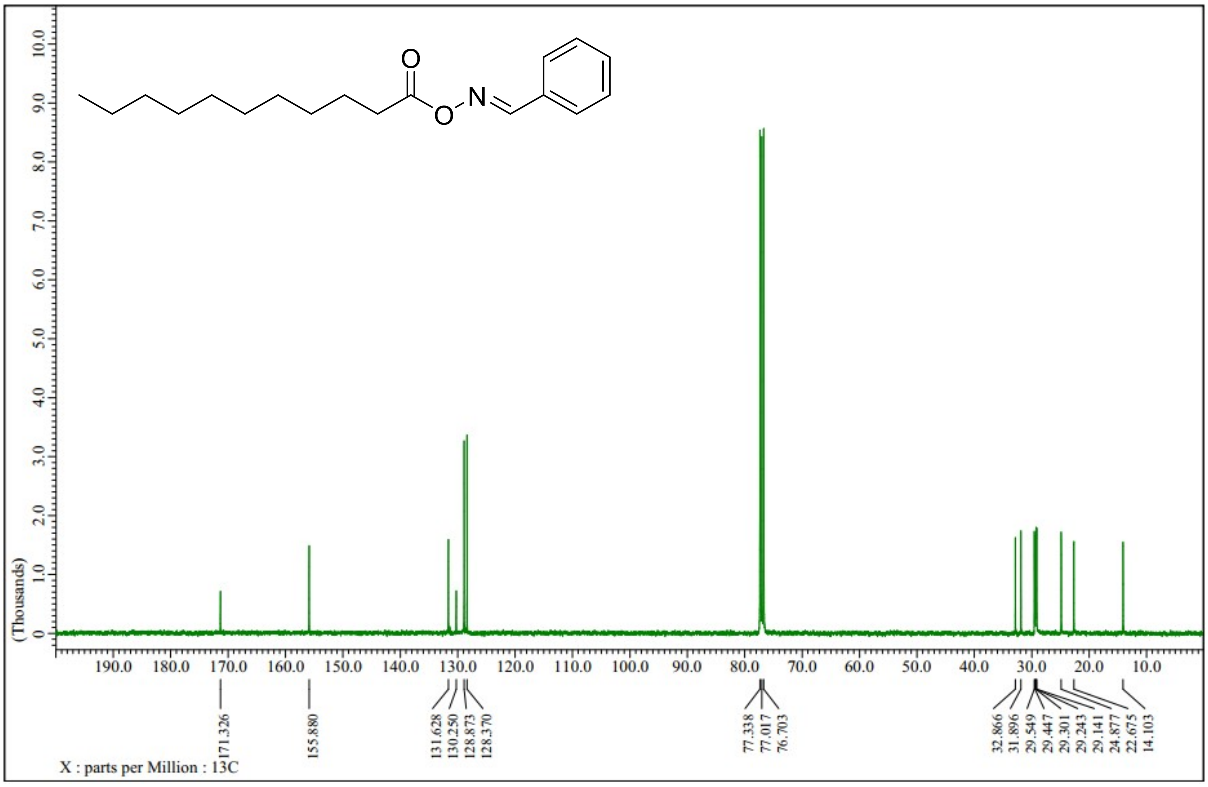

^{13}C NMR spectrum of (*E*)-benzaldehyde O-undecanoyl oxime (**5m**)

(*E*)-Benzaldehyde O-cyclohexanecarbonyl oxime (5n)

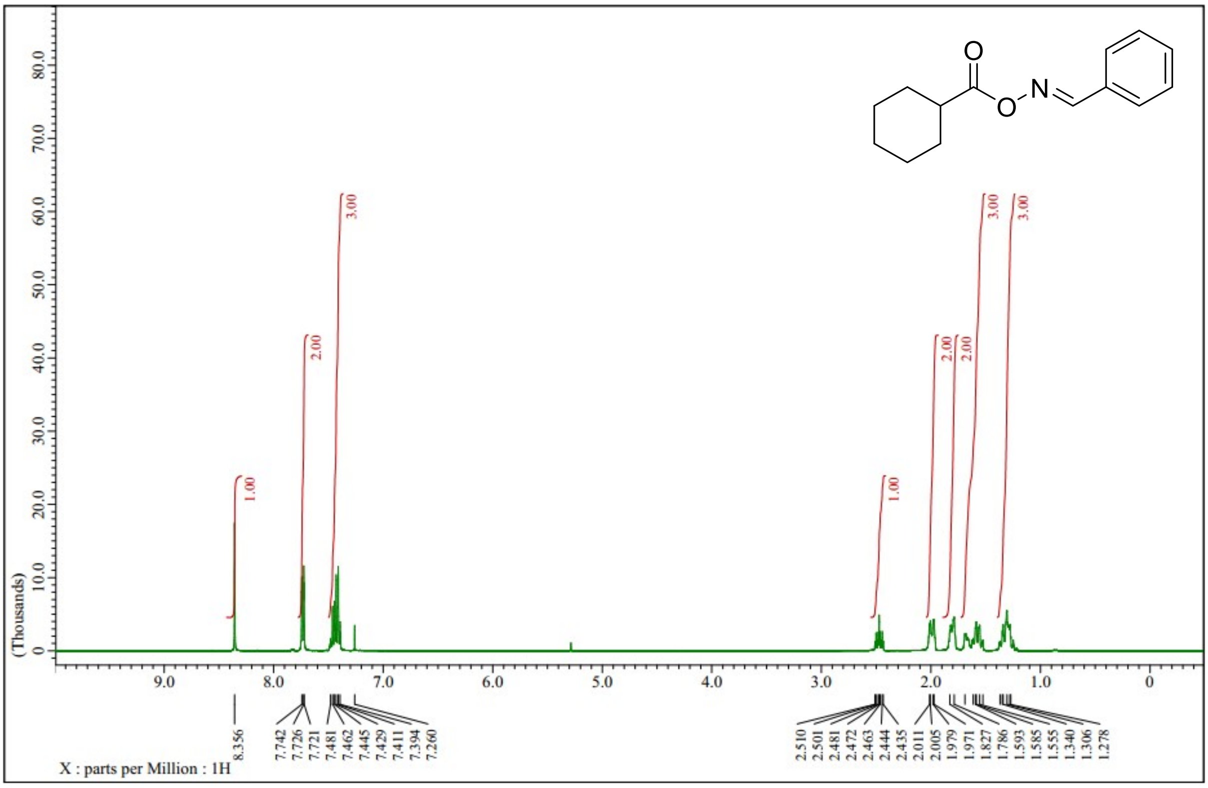

^{1}H NMR spectrum of (*E*)-benzaldehyde O-cyclohexanecarbonyl oxime (**5n**)

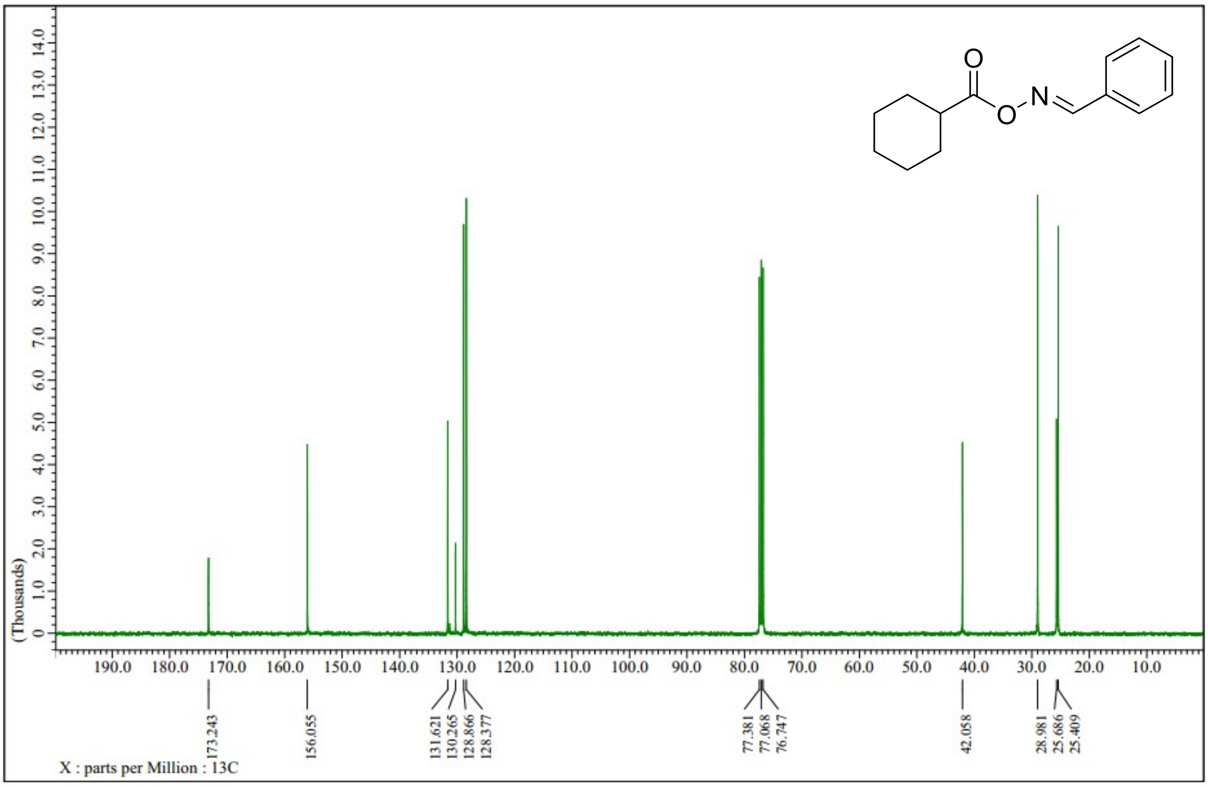

^{13}C NMR spectrum of (*E*)-benzaldehyde O-cyclohexanecarbonyl oxime (**5n**)

(*E*)-N,1-Diphenylmethanimine (6a)

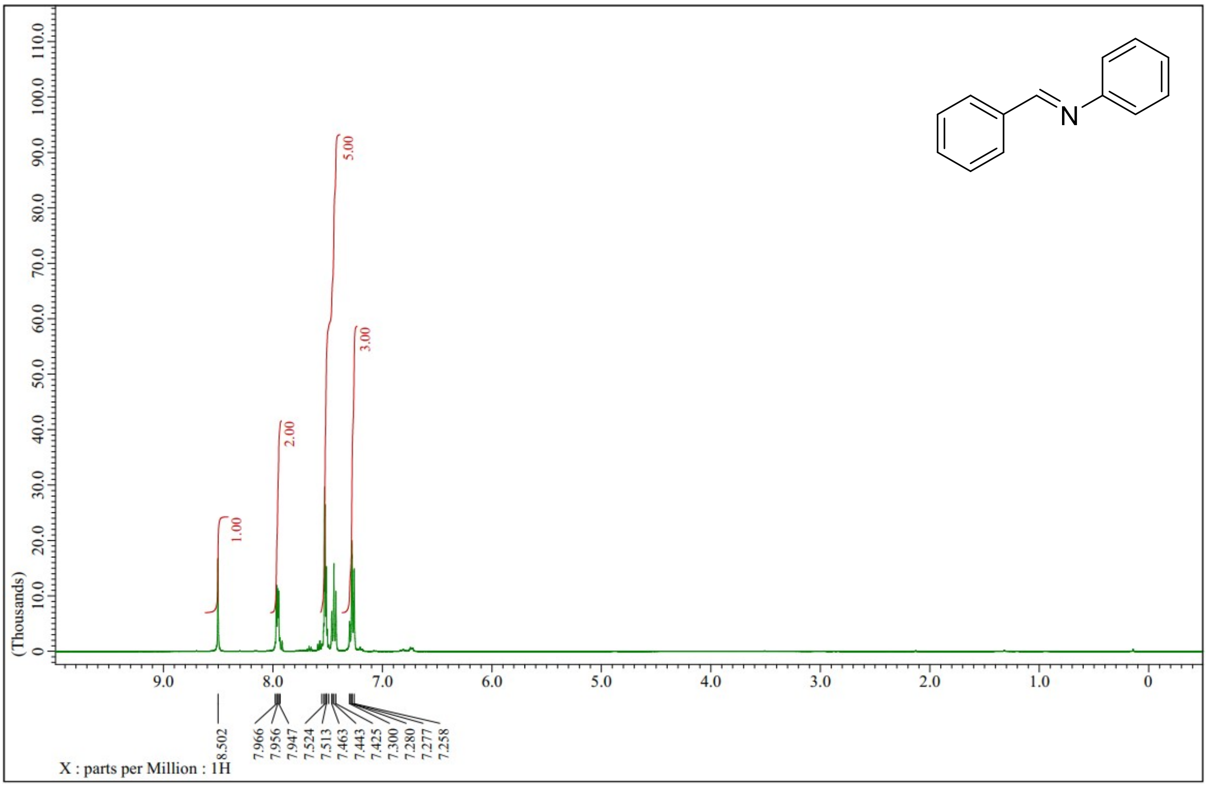

^{1}H NMR spectrum of (*E*)-N,1-diphenylmethanimine (**6a**)

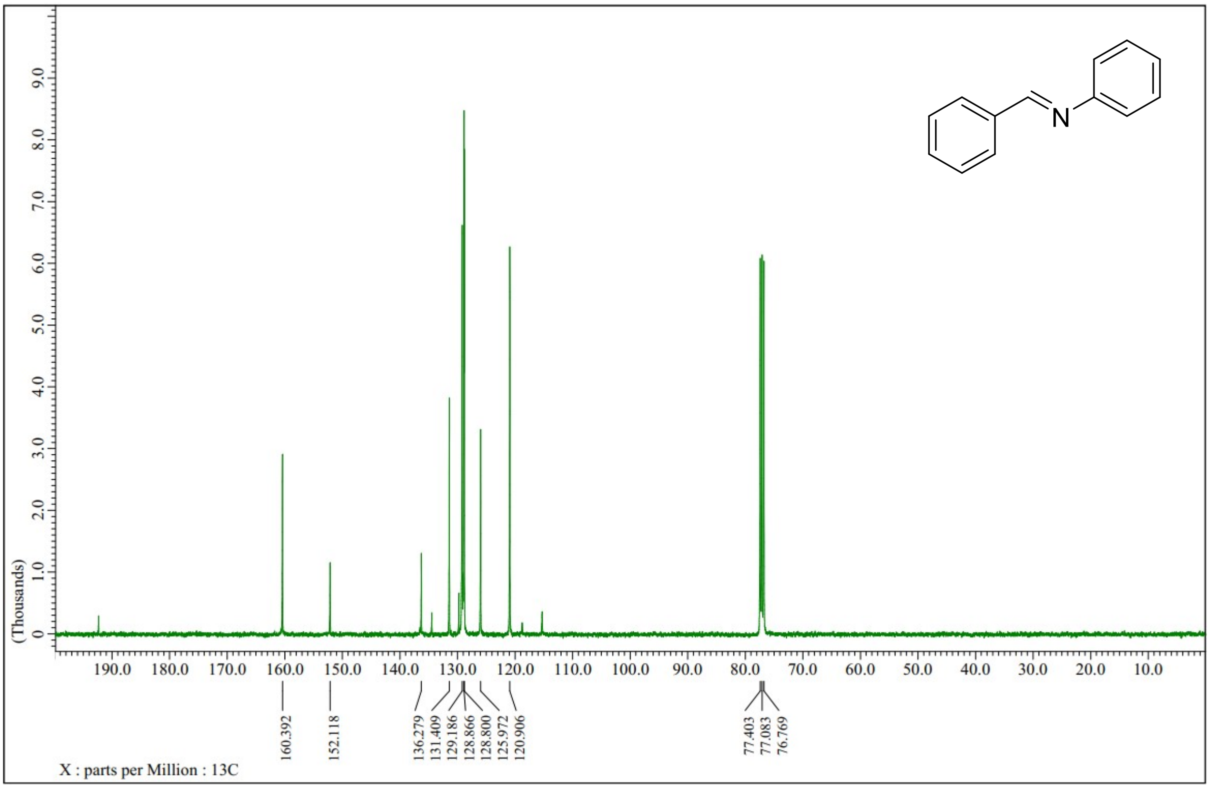

^{13}C NMR spectrum of (*E*)-N,1-diphenylmethanimine (**6a**)
